# Retinoic Acid Induces Embryonic Stem Cell Differentiation by Altering Both Encoding RNA and microRNA Expression

**DOI:** 10.1371/journal.pone.0132566

**Published:** 2015-07-10

**Authors:** Jingcheng Zhang, Yang Gao, Mengying Yu, Haibo Wu, Zhiying Ai, Yongyan Wu, Hongliang Liu, Juan Du, Zekun Guo, Yong Zhang

**Affiliations:** 1 College of Veterinary Medicine, Northwest A&F University, Yangling, 712100, Shaanxi, China; 2 Key Laboratory of Animal Biotechnology, Ministry of Agriculture, Northwest A&F University, Yangling, 712100, Shaanxi, China; 3 College of Life Sciences, Northwest A&F University, Yangling, 712100, Shaanxi, China; 4 College of Veterinary Medicine, China Agricultural University, Beijing, 100094, China; Goethe University, GERMANY

## Abstract

Retinoic acid (RA) is a vitamin A metabolite that is essential for early embryonic development and promotes stem cell neural lineage specification; however, little is known regarding the impact of RA on mRNA transcription and microRNA levels on embryonic stem cell differentiation. Here, we present mRNA microarray and microRNA high-output sequencing to clarify how RA regulates gene expression. Using mRNA microarray analysis, we showed that RA repressed pluripotency-associated genes while activating ectoderm markers in mouse embryonic stem cells (mESCs). Moreover, RA modulated the DNA methylation of mESCs by altering the expression of epigenetic-associated genes such as Dnmt3b and Dnmt3l. Furthermore, H3K4me2, a pluripotent histone modification, was repressed by RA stimulation. From microRNA sequence data, we identified two downregulated microRNAs, namely, miR-200b and miR-200c, which regulated the pluripotency of stem cells. We found that miR-200b or miR-200c deficiency suppressed the expression of pluripotent genes, including Oct4 and Nanog, and activated the expression of the ectodermal marker gene Nestin. These results demonstrate that retinoid induces mESCs to differentiate by regulating miR-200b/200c. Our findings provide the landscapes of mRNA and microRNA gene networks and indicate the crucial role of miR-200b/200c in the RA-induced differentiation of mESCs.

## Introduction

Mouse embryonic stem cells (mESCs) are derived from the inner cell mass of the embryo and can differentiate into precursors of all the three primary germ layers: ectoderm, endoderm, and mesoderm [1, 2]. In light of this pluripotency, ESC therapies have been developed for regenerative medicine and cell replacement. In mESCs, genes such as Oct4 and Nanog maintain pluripotency and prevent differentiation [3, 4], while signaling pathways involving Wnt, TGF-beta, BMP, and MEK/ERK guide mESCs toward cell fate [5–8]. Several epigenetic-associated genes, including the DNA methyltransferase (DNMTs) family, histone methylation, and histone deacetylation (HDACs), alter the genome epigenetics to influence stem cell differentiation [9, 10]. Recently, several studies have reported that microRNAs (miRNAs), small non-coding RNA molecules containing approximately 22–25 nucleotides [11, 12], also play crucial roles in embryonic development, suggesting that ESC differentiation requires the coordinated regulation of miRNA networks.

Retinoic acid (RA) is a metabolite of vitamin A, involved in inflammation, cell differentiation, and embryonic development [13, 14]. In early embryonic development, RA guides the development of the posterior portion of the embryo through the regulation of Hox family genes [15], which control anterior and posterior patterning in early embryonic development [16, 17]. Moreover, RA promotes stem cell neural lineage specification and neuron differentiation [18–20]. However, the regulation of mRNAs and miRNAs by RA in mESCs is largely unexplored.

In this study, we performed mRNA microarray and small RNA (sRNA) high-throughput sequencing to identify genes and miRNAs that are differentially expressed by J1 mESCs in the presence of RA. Our data demonstrated that RA modifies pluripotency genes via miR-200b/200c. Thus, miR-200b and miR-200c are RA-modulated miRNAs that control changes in downstream gene expression patterns required for RA-induced differentiation.

## Results

### Microarray profiling demonstrates that RA induces ectodermal marker expression in ES cells

**Fig 1:**
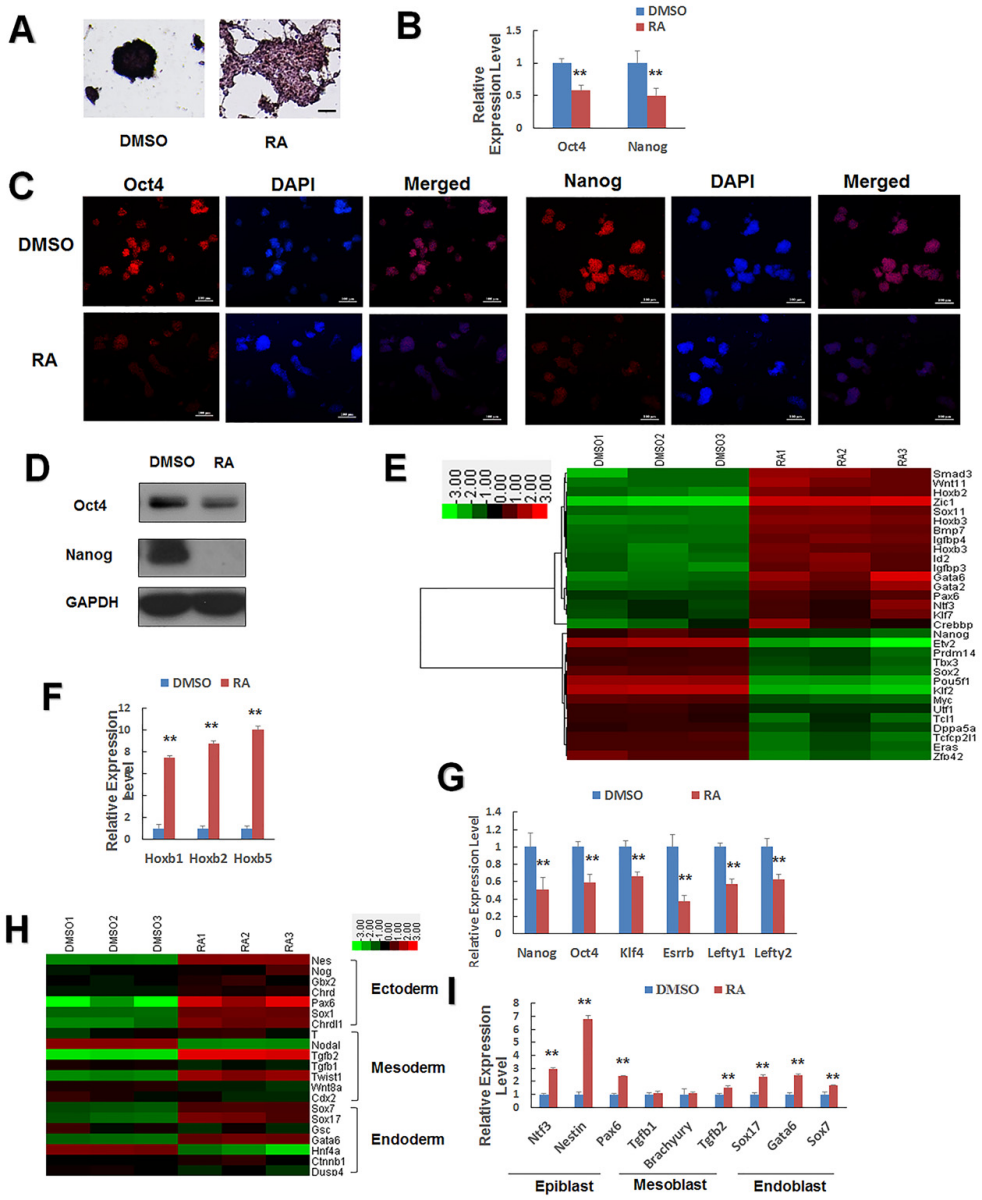
Changes in the expression of genes involved in ESC self-renewal and differentiation, following retinoic acid (RA) treatment. (A) Alkaline phosphatase staining of J1 mESCs cultured on gelatin-coated plates without LIF and treated with 1 μM RA or DMSO for 24 h. Alkaline phosphatase staining is an indicator of pluripotency. Scale bars = 100 μm. (B, C, D) qPCR, immunofluorescence, and western blot analysis of Oct4 and Nanog expression in J1 mESCs. DAPI was used for nuclear staining. Scale bars represent 100 μm. (E) Expression pattern of the representative upregulated differentiation-associated genes and downregulated pluripotency-related genes. Low intensities are shown in green and high intensities in red. mESCs were treated with DMSO or RA. (F) Representative differentiation-associated genes. The Hoxb gene was upregulated significantly by RA treatment. (G) Representative pluripotency-related genes downregulated in the presence of RA were validated by qPCR. (H) Heatmap diagram of three germ cell marker genes treated with or without RA. Epiblast marker expression was significantly altered by RA. (I) qPCR analysis validated these trends for the altered expression of genes related to growth and development of mESCs. J1 mESCs cultured with DMSO (vehicle) or 1 μM RA for 24 h. Data are presented as the mean ± SEM of three independent experiments (*p < 0.05; **p < 0.01).

To assess the function of RA in mESC differentiation, mESCs were cultured with or without RA for 24 h. We found that mESCs showed a low alkaline phosphatase activity (AP) and lost colonies after RA treatment (Fig 1A). To confirm that RA regulated the pluripotency of mESC, we performed qPCR and western blotting to measure the mRNA and protein levels of the pluripotency-associated genes Oct4 and Nanog [21]. Both Oct4 and Nanog were suppressed by RA (Fig 1B–1D).

To gain a global view of the impact of RA, we performed gene expression microarray analysis. From the expression data, we identified 1132 genes that were significantly downregulated [Fold-change (FC) ≤ 0.5, p value ≤ 0.01, S1 Table] and 1093 genes that were significantly upregulated (FC ≥ 2, p value ≤ 0.01, S2 Table) by RA treatment. We detected differentiation-associated genes Hoxb1, Hoxb2, and Hoxb3 [22, 23], while the pluripotency-associated genes Oct4, Nanog, Klf4, Esrrb, Lefty1, and Letfy2 were downregulated by RA treatment (Fig 1E) [24–26]. These changes were confirmed by qPCR (Fig 1F and 1G). We analyzed the regulation of lineage-specific genes and constructed a heatmap of the germ-line marked genes (Fig 1H). This heatmap expression level of the ectodermal markers Nestin, Noggin, Gbx2, Chordin, Chrdl1, and Pax6 were increased by RA treatment, whereas the expression levels of the mesodermal and endoderm markers Brachyury (T), Tgfb1, Wnt8a, Cdx2, Dusp4, Ctnnb1, and Goosecoid were not changed significantly by RA. However, other mesodermal and endodermal markers, including Tgfb2, Twist1, Sox7, Sox17, and Gata6, were upregulated and Hnf4a and Nodal were downregulated [27–31]. The relative changes in the expression of the ectodermal markers were more pronounced than the changes in other blastodermal markers (Fig 1H), indicating that RA differentiation favors the ectodermal lineage. We chose three marker genes from each germ-line and validated the microarray assay result by qPCR (Fig 1I). Taken together, we demonstrated that mESCs were induced to differentiate by RA treatment through the repression of pluripotency genes and induction of genes related to differentiation, particularly genes associated with the ectodermal lineage.

### RA regulates epigenetic modifications in embryonic stem cells

**Fig 2:**
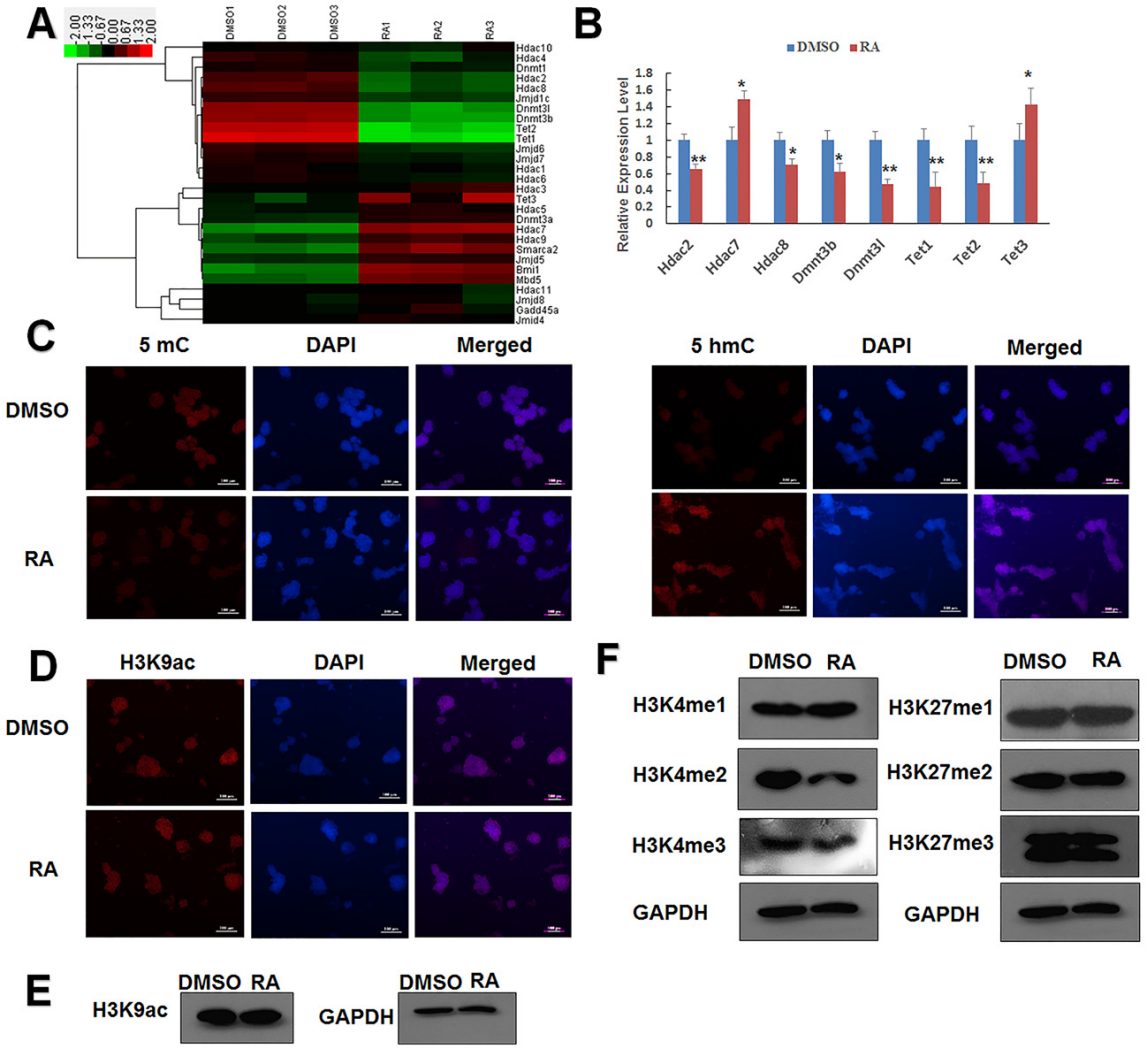
Regulation of epigenetic regulatory gene expression by RA treatment. (A) Expression pattern of representative epigenetic-associated genes identified by microarray data in J1 mESCs. High expression levels are shown in green and low in red. The control group was treated with DMSO. (B) Representative epigenetic-related genes differently expressed in the presence of 1 μM RA were validated by qPCR. (C) Immunostaining assay of global DNA methylation by measurement of levels of 5-mC and 5-hmC in J1 mESCs. DAPI was used for nuclear staining. Scale bars represent 100 μm. (D) Immunostaining assay of H3K9Ac in J1 mESCs. DAPI was used for nuclear staining. Scale bars represent 100 μm. J1 mESCs were cultured in the presence or absence of 1 μM RA for 24 h. Data are presented as the mean ± SEM of three independent experiments (*p < 0.05; **p < 0.01). (E) Western blot detected the H3K9ac level. J1 mESCs were cultured in the presence or absence of 1 μM RA for 24 h. (F) Western blot detected the H3K4 mono-, di-, tri-methylation and H3K27 mono-, di-, tri-methylation levels. J1 mESCs were cultured in the presence or absence of 1 μM RA for 24 h.

Epigenetics is thought to control several biological processes by epigenetic phenomena such as DNA methylation and histone modification but not by gene mutations [32, 33]. Epigenetic changes are critical during early embryonic development and stem cell differentiation [34–37]. To explore the contribution of epigenetic modifications to RA-induced differentiation of mESCs, we analyzed the microarray data and found that the DNA-methylation-related genes Dnmt3b, Dnmt3l, Tet1, and Tet2 were downregulated by RA treatment, whereas Tet3 was upregulated (Fig 2A). We also found that the expression levels of the histone-acetylation-related genes Hdac2, Hdac7, and Hdac8 were regulated by RA treatment (Fig 2A). These microarray results were confirmed by qPCR (Fig 2B), which showed qualitatively similar changes in these genes.

Immunofluorescence staining was used to investigate global epigenetic modifications in mESCs. Immunostaining with 5-methylcytosine (5-mC) indicated that the global 5-mC level was repressed after RA treatment, while 5-hydroxymethylcytosine (5-hmC) staining revealed that the global 5-hmC level was promoted by RA treatment (Fig 2C). We also examined histone H3 lysine 9 acetylation (H3K9ac) and found that the H3k9ac level was not remarkably changed (Fig 2D and 2E), although the expression levels of Hdac2 and Hdac8 were downregulated. In addition, we detected that Hdac7, one of the class II HDACs, was upgraded after RA treatment. These results suggest that RA treatment induces epigenetic modification, which is the most changed in DNA methylation.

Although RA does not regulate many histone modifiers at the mRNA level, it may indirectly affect their activity. To evaluate whether RA induces epigenetic modifications other than DNA methylation, we detected other modifications in histone methylation. Western blot showed that H3K4me2 was repressed with RA treatment (Fig 2F). These results demonstrate that epigenetic modifications such as DNA methylation, histone methylation, and histone acetylation were altered in RA-induced differentiated mESCs.

### GO annotation and KEGG Pathway analysis of RA-treated mESCs transcripts

**Fig 3:**
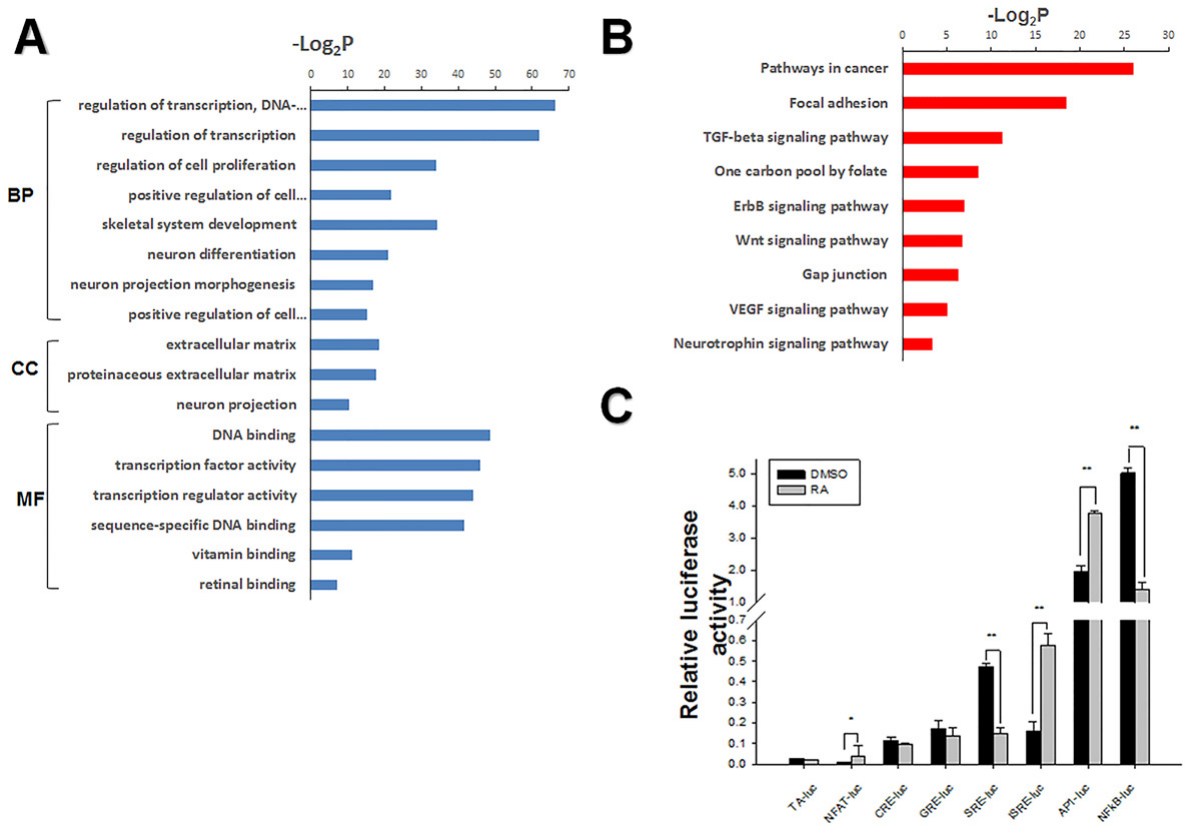
GO annotation and Pathway screening to identify signaling pathways involved in RA-induced differentiation of mESCs. (A) GO categories for comparison of differently expressed genes identified by microarray analysis. Using the DAVID online system, significantly regulated genes were grouped into three main GO categories: biological processes (BP), cellular components (CC), and molecular functions (MF) (shown as a—log2P value). Significantly regulated genes according to fold change (FC) in gene expression (FC ≥ 2 or FC ≤ 0.5). (B) KEGG pathway categories of significantly differentially expressed genes (2225) in mESCs after RA stimulation. (C) Signaling pathway profiling of mESCs in response to RA stimulation for 24 h. Cells co-transfected with reporter plasmids and internal control pRL-SV40. Dual luciferase assays of pathway reporters showed that NF-kB, NFAT, SRE, and AP1 signaling were significantly activated by RA. Data represented as mean ± SEM of triplicate experiments. Pathway reporters used for screening and their represented pathways. Reporter plasmids for signaling pathways involving PKC and Ca2+/calcineurin (pNFAT-luc), PKA (pCRE-TA-luc), glucocorticoid/HSP90 (pGRE-TA-luc), MAPK/JNK (pSRE-TA-luc), proliferation/inflammation (pISRE-TA-luc), NFκB (pNFκB-TA-luc), and JNK/p38 (pAP1-TA-luc). RA (1 μM) or equal volume DMSO was added to the mESCs for 24 h, followed by cell lysis. Luciferase activity is expressed relative to that of pTA-luc. The values for relative luciferase activities are shown in the bar graphs (*p < 0.05; **p < 0.01).

To better understand the specific mechanism of RA-induced differentiation, we performed GO annotation and KEGG pathway analysis. We observed an enrichment of the neuroectodermal differentiation and skeletal system development markers Sox1, Tgfb2, Ntf3, Pax6, Id2, Fgf18, and Thra by GO-term of analysis of genes differentially expressed between RA-treated and untreated mESCs (Fig 3A), consistent with RA-induced activation of ectodermal gene expression (Fig 1H and 1I). In addition, the GO-term results showed that RA altered the expression of genes involved in the regulation of transcription, cell proliferation, cell differentiation, DNA binding, and transcription factor activity. Thus, these results indicate that RA induces ESC differentiation by altering the expression of an extensive network of genes.

To identify the signaling pathways associated with RA-induced differentiation of ESCs, we analyzed differentially regulated genes using the DAVID online program. The result showed that the most regulated genes are associated with pathways related to cancer, focal adhesion, one carbon pool by folate, and TGF-beta signaling pathway, neurotrophin signaling pathway (Fig 3B), consistent with RA-regulated cell proliferation and differentiation, DNA methylation, and neurodevelopment. To confirm these predictions, we use luciferase reporter assays to directly examine the activation of these signaling pathways. RA significantly regulated PKC and Ca2+/calcineurin, MAPK/JNK, JNK/p38, and NFκB, pathways known to be associated with proliferation and neurodevelopment (Fig 3C). GO annotation and KEGG pathway analysis of differentially expressed genes indicated that RA activated or repressed multiple signal pathway to regulate cell proliferation and differentiation.

### Multiple microRNA are suppressed in mESCs by RA treatment

**Fig 4:**
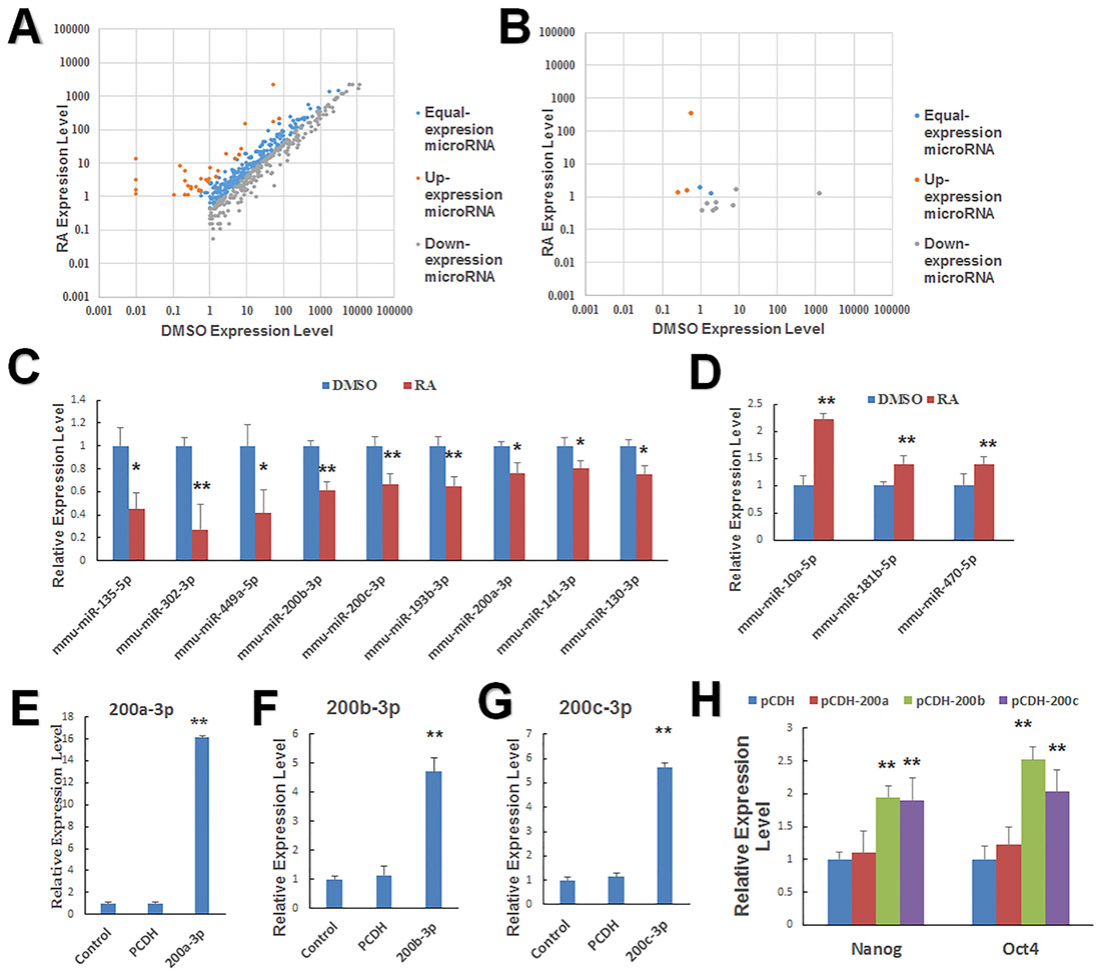
Altered expression of miRNAs in mESCs by RA treatment. (A) Scatter plot of the miRNA expression differences between DMSO- and RA-treated samples. (B) Scatter plot of the predicted novel miRNA expression between DMSO- and RA-treated cells showing differentially expressed miRNAs in the two samples. (C) RA downregulated the expression of miRNAs associated with pluripotency of mESCs, a finding validated by qPCR. mESCs were treated with 1 μM RA or DMSO for 24 h. (D) qPCR determined that RA upregulated the expression of differentiation-associated miRNAs. Representative miRNAs that were downregulated after treatment with 1 μM RA for 24 h. (E, F, and G) MiR-200a, miR-200b, and miR-200c relative expression levels after transfection with the expression vector PCDH-miR-200b or PCDH-miR-200c for 48 h. (H) qPCR revealed enhanced Oct4 and Nanog expression levels after transfection with the expression vectors PCDH-miR200a, PCDH-miR200b, or PCDH-miR200c for 48 h (without RA). Data are presented as the mean ± SEM of three independent experiments (*p < 0.05; **p < 0.01).

MiRNAs also play important roles in early embryonic development [38]. Although we have demonstrated that RA could stimulate mESC proliferation and differentiation by regulating gene expression, it is not clear if RA modulates miRNA expression to control the fate of stem cells. We performed sRNA sequencing and identified miRNAs that are differentially expressed by RA-treated and untreated mESCs. From the sequencing data, we identified 31 miRNAs that were significantly upregulated (FC ≥ 2.5, p ≤ 0.01) and 175 miRNAs that were significantly downregulated by RA treatment (FC ≤ 0.4, p ≤ 0.01) (S2 and S3 Tables). These differentially expressed miRNAs are displayed on the scatter plot (Fig 4A). We also used the prediction software Mireap to find potential novel miRNAs by exploring the secondary structure and found several differentially expressed novel miRNAs (Fig 4B). Furthermore, qPCR was performed again to validate the downregulated and upregulated expression of selected miRNAs that may be relevant to development and confirmed that miR-135, miR-302, miR-449a, miR-200b, miR-200c, miR-193b, miR-130, and miR-141 were downregulated, whereas miR-10a, miR-181, and miR-470 were upregulated by RA treatment (Fig 4C and 4D).

miR-200s family is a key determining factor of the epithelial phenotype [39]. Thus, we investigated the role of miR-200a, miR-200b, and 200c in the determination of the RA-induced epithelial phenotype. We constructed expression vectors for miR-200a,miR-200b, and miR-200c (Fig 4E, 4F and 4G). We found that Oct4 and Nanog were significantly increased after the overexpression of miR-200b/c and miR-200a had no effect on Oct4 and Nanog (Fig 4H). Therefore, we only focussed on miR-200b/c in the following research.

Overall, the data showed that RA downregulated multiple miRNAs. In addition, we found that miR-200b and 200c, two of the downregulated miRNAs, significantly increased the expression of two pluripotent genes Oct4 and Nanog in mESCs.

### Retinoic acid inhibited the pluripotency of stem cells by supressing the expression of miR-200b and miR-200c

**Fig 5:**
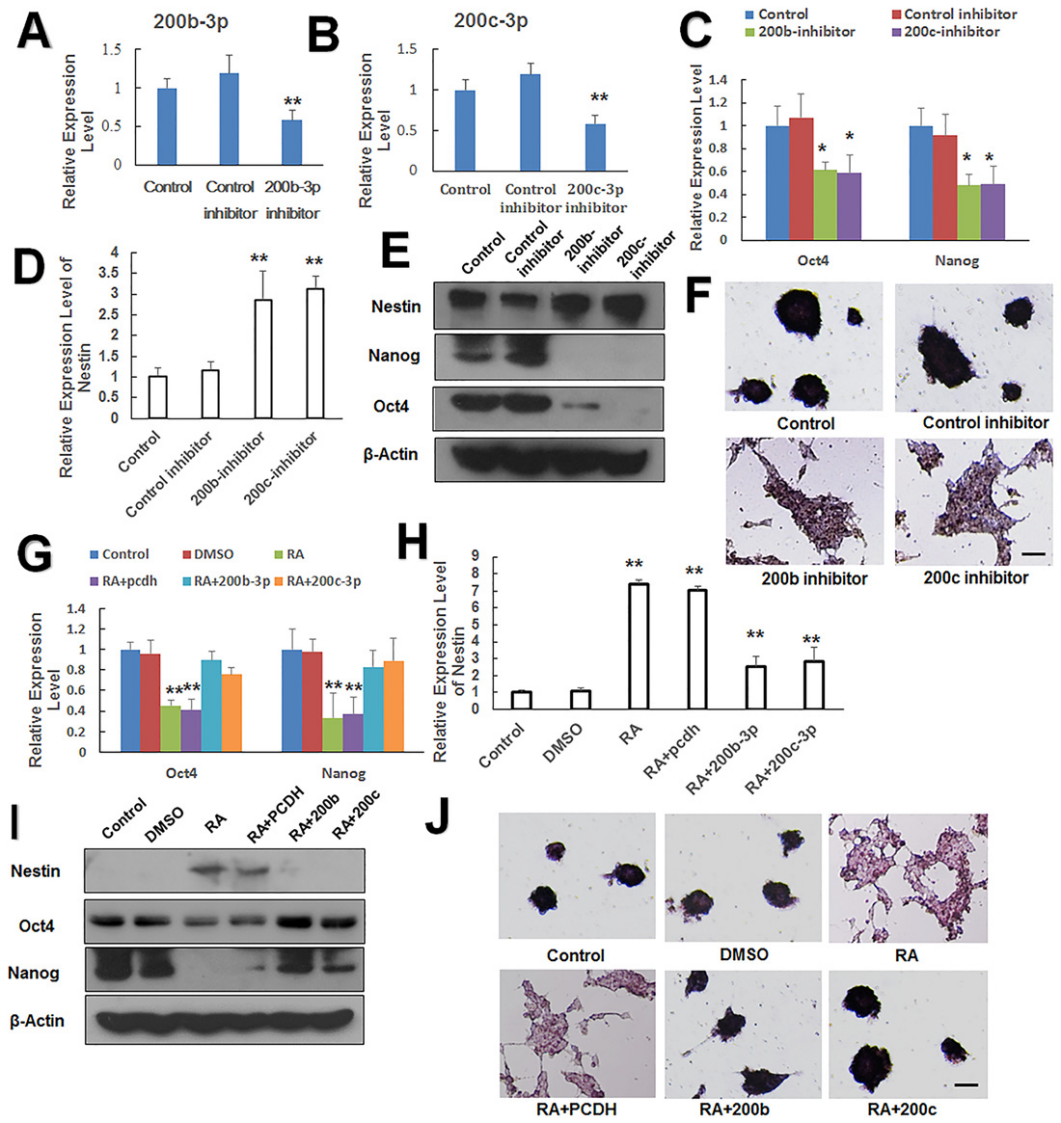
Retinoic acid-induced differentiation is mediated by the suppression of miR-200b and miR-200c. (A and B) MiR-200b/c inhibitor and their negative controls were transfected into J1 ES cells, and the expression of miR-200b or 200c was detected by qPCR. (C) qPCR analysis of Oct4 and Nanog following miR-200b and miR-200c inhibitor transfection into J1 ES cells for 48 h. (D) qPCR analysis of Nestin following miR-200b and miR-200c inhibitor transfection into J1 ES cells for 48 h. (E) The relative levels of Oct4, Nanog, and Nestin detected by western blot after miR-200b and miR-200c inhibitors were pre-transfected into J1 ES cells for 48 h. (F) Alkaline phosphatase staining to determine pluripotency following transfection of miR-200b and miR-200c inhibitors into J1 ES cells for 48 h. Scale bars = 100 μm. (G) qPCR analysis of Oct4 and Nanog expression following transfection with miR-200b and miR-200c expression vectors into J1 ES cells for 24 h and treatment with RA for an additional 24 h. (H) Nestin expression following transfection with miR-200b and miR-200c expression vectors into J1 ES cells for 24 h and treatment with RA for an additional 24 h. (I) Western blot analysis of Oct4 Nanog, and Nestin expression following transfection with miR-200b and miR-200c expression vectors into J1 ES cells for 24 h and treatment with RA for an additional 24 h. (J) Alkaline phosphatase staining for pluripotency in J1 ES cells transfected with miR-200b and miR-200c expression vectors for 24 h and then treated with RA for 24 h. Scale bars = 100 μm. Data are presented as the mean ±SEM of three independent experiments. (*p < 0.05; **p < 0.01).

We confirmed that RA inhibited miR-200b and 200c (Fig 4C), factors that serve to increase the expression of the pluripotent genes Oct4 and Nanog (Fig 4G). We speculated that RA represses these two miRNAs, thereby reducing Oct4 and Nanog expression, leading to mESC differentiation. To test this idea, we transfected mESCs with the miR-200b inhibitor or miR-200c inhibitor and verified the effect of the inhibitor by q-PCR (Fig 5A and 5B). We then performed qPCR and western blotting 48 h after transfection to identify changes in the expression levels of pluripotent genes and ectodermal markers. As predicted, the pluripotent genes Oct4 and Nanog were downregulated (Fig 5C and 5E), whereas the ectoderm marker gene Nestin was upregulated (Fig 5D and 5E). Loss of AP activity confirmed the differentiation of mESCs and loss of pluripotency after transfection of miR-200b and miR-200c inhibitors (Fig 5F). Thus, downregulation of the expression of miR-200b or miR-200c promoted the differentiation of mESCs.

To provide further confirmation that deficiency of miR-200b or miR-200c caused mESCs differentiation, mESCs were transfected with the expression vector for miR-200b or miR-200c and 24 h later treated with RA for an additional 24 h. Both qPCR and western blotting indicated that miR-200b/200c antagonized RA-induced Nestin upregulation (Fig 5H and 5I) as well as Oct4 and Nanog downregulation in mESCs (Fig 5G and 5I). Moreover, AP activity was maintained upon RA-treated mESCs transfected with miR-200b or miR-200c (Fig 5J). Our data demonstrated that the expression of miR-200b and miR-200c antagonized RA-induced differentiation in mESCs, consistent with the observation that miR-200b/200c deficiency in mESCs promoted differentiation in the absence of RA. We conclude that RA induced mESC differentiation by repressing miR-200b and miR-200c factors is essential for maintaining the pluripotency of mESCs.

## Discussion

RA is involved in a variety of processes during early embryonic development, including proliferation, differentiation, and organogenesis [40–42]. However, little is known regarding the downstream changes in mRNA and miRNA levels induced in the early stage of RA stimulation. In this study, we revealed that RA regulates mRNA levels through changes in miRNA expression in mESCs. Using microarray analysis, we showed that RA reduced the pluripotency of mESCs by repressing pluripotency genes and induced their differentiation to ectodermal cells. RA-dependent differentiation may also require epigenetic modifications because RA altered the expression of genes associated with DNA methylation, histone acetylation, and histone methylation. Pathway analysis indicated that RA regulated several pathways related to proliferation. We performed sRNA high-output sequencing analysis to generate sRNA expression profiles and identified several miRNAs differentially expressed between RA-treated and untreated mESCs. From the significantly altered miRNAs, we examined the roles of two pluripotency-associated miRNAs, miR-200b and miR-200c [43], and further confirmed that RA partially induced mESC differentiation by inhibiting miR-200b and miR-200c expression, which in turn led to the downregulation of pluripotency genes that normally serve to retard differentiation into ectoderm germ cells.

Firstly, from the expression profiling, we detected that RA activated many markers of all three germlines. However, the ectoderm markers Nestin, Noggin, Pax6, Chrdl1, and Sox1 were upregulated more strongly than those related to other lineages. In addition, multiple genes related to neurodifferentiation were upregulated.

Furthermore, we found that RA altered the expression of genes associated with DNA methylation and histone acetylation (Fig 2A). Upon RA stimulation, two DNA methylases Dnmt3b and Dnmt3l were repressed. The global change of DNA methylases may be associated with these two de novo DNA methylases [44]. The expression of Dnmt3b decreased because the differentiation of mESCs and Dnmt3l knock down induced neuroectodermal markers for gene upregulation [45, 46]. The global change of 5-hmc may be because of repressed DNMTs and increased Tet3 [44]. RA upregulated Tet3 while repressing both Tet1 and Tet2 to skew the differentiation of mESCs into the ectoderm [47, 48]. In addition, we detected histone-methylated modifications. The H3K4me2 level decreased with RA treatment. A recent study demonstrated that heavy downstream H3K4me2 levels were obtained during different stages that decreased with respect to MEF and EMT genes and increased in proliferation, metabolism, and pluripotency with respect to MET genes [49, 50]. Therefore, we hypothesized that RA could decrease H3K4me2 levels to activate subsequent multilineage differentiation.

The absence of Hdac2 induced the expression of a differentiation gene Sox10 [51]. In addition, Hdac8 is reported to link with smooth muscle mesoderm tissues [52]. We examined that the expression levels of Hdac2 and Hdac8 were downregulated, indicating that RA could induce the differentiation of mESCs by altering the expression levels of Hdac2 and Hdac8 (Fig 2B). HDAC3, an important deacetylase in neurodevelopment [53, 54], was not significantly altered (FC = 0.798291746, p = 0.144619062). HDACs are DNA-binding proteins; therefore, we presumed that RA recruited other DNA-binding proteins to occupy the Hdac3-binding regions; this reduced Hdac3 enrichment of neuroectoderm-differentiated genes. In agreement with this scenario, recent evidence indicates that RA regulates class I Hdac3 differential binding to change gene expression [55]. From microarray analysis data and q-PCR results, we found that Hdac7 expression was upregulated after RA treatment (Fig 2A and 2B). This upregulation may activate some lineage-specific genes to induce the differentiation of mESCs with RA stimulation. Hdac7 genes are highly expressed in hippocampal progenitor cells and bind to 14-3-3 proteins, which are strongly expressed in the neural tissue. In addition, Hdac7 processes the expression of the mesodermal-specific proteins Desmin and Flk-1 [56–58]. These results suggest that Hdac7 promotes stem cell differentiation and that RA induces the differentiation by increasing Hdac7 expression.

Moreover, the luciferase pathway analysis revealed that the Ca2+/calcineurin-dependent nuclear factor of activated T cells (NFAT) pathway was regulated by RA, a signaling pathway known to be involved in neurodevelopment [59]. These results indicated that RA induced the differentiation of mESCs into ectoderm cells by altering the transcriptional levels of genes associated with this phenotype and are consistent with previous studies conducted on the RA effects at the level of cells and embryos. In early embryonic development, we found that RA treatment may have altered DNA methylation by regulating genes related to DNA methylation, such as Dnmt3b and Dnmt3l. KEGG analysis indicated that RA regulated the one carbon pool, which is also critical for DNA methylation [60, 61].

In embryonic stem cells, miRNAs regulate the self-renewal of mESCs and maintain pluripotency [12, 62]. The miR-200 family is considered essential for tumor development by regulating epithelial transition [63]. Recently, *Wang et al*. found that miR-200s induced somatic cell reprogramming to pluripotency by activating the pluripotent markers Oct4 and Sox2 [64]. In this model, RA treatment inhibited miR-200 family expression. We chose members of the miR-200 family, miR-200a, miR-200b, and miR-200c because these are abundantly expressed in mESCs and demonstrated that upregulation of miR-200b or miR-200c increased the expression of two key embryonic stemness genes Oct4 and Nanog, thereby promoting pluripotency [65]. A previous study detected that miR-200a could increase Oct4 expression slightly in ES-E14TG2a cells and had no effect on Nanog [66]. In our research, both Oct4 and Nanog were not significantly effected by miR-200a in J1 cells (Fig 4H), which may be attributed to different cell types. Thus, we did not conduct further research on miR-200a. To further clarify the miR-200b/c roles in RA-induced differentiation, we used an inhibitor of miR-200b and miR-200c to substitute the RA-induced deficiency. We found that repressed miR-200b and miR-200c could reduce the pluripotency of mESCs in the absence of RA. Moreover, miR-200b and miR-200c expression antagonized RA-induced differentiation, whereas inhibition of miR-200b and miR-200c expression enhanced RA-induced suppression of the pluripotency genes Oct4 and Nanog and promoted RA-induced Nestin upregulation. Therefore, we concluded that RA induced the differentiation of mESCs by repressing miR-200b and miR-200c factors essential for maintaining the pluripotency of mESCs. Some recent research found that the endoderm lineage markers Gata4, transcription factor Tcfap2a, and neural development markers Ntf3 were the targets of miR-200b/c [65, 67, 68]. Ntf3 could induce Nestin expression [69]. Tcfap2a binds to the Oct4 promoter to repress Oct4 [70]. Gata4 enabled the direct repression of Nanog [65]. Therefore, RA could inhibit miR-200b/c to activate Gata4 and Ntf3 and could repress Nanog plus Oct4 and upgrade Nestin expression. The overexpression of miR-200b/c could block the stemness markers Klf4 and Sox2 (S1 Fig) [71]; however, Sox2 is also required for the maintenance of self-renewal of neural stem/progenitor cells. In addition, Klf4 is highly expressed in both ESCs and NSCs [72, 73]. Golebiewska’s research also proves that the expression of the pluripotency marker Sox2 in H9-derived NSCs is slightly higher than H9-hESCs, although Oct4 and Nanog are decreased in NSCs [74]. Therefore, inhibiting some stemness markers could partially help in maintaining the pluripotency. In ESCs, Oct4 is the sole stemness marker, while Nanog is the gatekeeper of pluripotency [75, 76]. Thus, the phenomena of Sox2 and Klf4 does not conflict our results. These observations strongly suggest that the two important stemness markers, Nanog and Oct4, were regulated by RA through its repression of miR-200b/c expression, and miR-200b and miR-200c play vital roles in RA-induced differentiation of mESCs.

In summary, RA strongly influences gene expression, epigenetic modification, signal pathways, and miRNA expression in mESCs, ultimately resulting in differentiation. In particular, miR-200b and miR-200c play vital roles in RA-induced differentiation of mESCs by suppressing genes associated with the maintenance of pluripotency. This foundational description of an RA-miR-200b/200c-gene regulation network will aid in the further study of retinoid functions in embryonic development.

## Materials and Methods

### Reagents

Primary mouse anti-Oct4, anti-Sox2, and anti-Klf4 were obtained from Santa Cruz Biotechnology (Santa Cruz, CA, USA); rabbit anti-mono-H3K27 from Abcam; rabbit anti-mono-,di-,tri-H3K4, rabbit anti-di-,tri-H3K27, and rabbit anti-Nanog from Cell Signaling Technology (Danvers, MA, USA); rabbit anti-Nestin from Sigma (Sigma, St. Louis, MO, USA); and anti-β-tubulin and anti-β-actin from Beyotime Institute of Biotechnology (Jiangsu, China). miRNA inhibitors were purchased from Biomics Biotechnologies (Jiangsu, China).

### Cell culture and treatment

J1 mouse ESCs were purchased from ATCC (Manassas, VA, www.atcc.org) and were maintained at 37°C, with 5% CO_2_ humidified air on 0.2% gelatin-coated plates with ESC medium consisting of Knockout DMEM supplemented with 15% (v/v) Knockout Serum Replacement, nonessential amino acids, 100 μM β-mercaptoethanol, 2 mM glutamine (Life Technologies Inc., NY, USA), and 1000 U/mL LIF (ESGRO, Millipore,110 USA).

Retinoic acid was purchased from Sigma (St. Louis, MO) and was dissolved in dimethyl sulfoxide (DMSO) at 1 μM (working concentration).

### Construction of plasmids

The pri-miRNA miR-200a, miR-200b and miR-200c were amplified from the J1 genome and were inserted into the multiple cloning site (MCS) of the expression vector pCDH-CMV-MCS-EF1-copGFP (System Biosciences, CA, USA). The primer sequences used for plasmid construction are shown in S7 Table.

### Pathway reporter assays

Pathway reporter vectors pAP1-TA-luc, pISRE-TA-luc, and pNFκB-TA-luc were bought from Beyotime. The control vector pTA-luc and pathway reporter plasmid pNFAT-TA-luc were bought from Clonetech (Mountain View, CA, USA). Vectors pCRE-TA-luc, pHSE-TA-luc, and pGRE-TA-luc were constructed in our laboratory. Reporter vectors and negative control vectors (pTA-luc) were co-transfected with pathway reporter plasmids and the Renilla luciferase vector pRL-SV40 (Promega, normalized to Renilla luciferase). Different reporter vectors contain corresponding specific cis-acting DNA sequences (enhancer elements) and a sensitive reporter gene. Thus, we could screen the binding of transcription factors to their corresponding enhancer elements and detect the key signaling pathways. Mouse ESCs were transfected using the Lipofectamine 2000 Reagent (Invitrogen) for 24 h, followed by treatment with either DMSO (vehicle) or RA for an additional 24 h. Luciferase activity was detected by a luciferase reporter assay system (Promega) according to the manufacturer’s instructions on a VICTOR X5 Multilabel Plate Reader (PerkinElmer, Cetus, Norwalk, USA).

### RNA isolation, microarrays and sRNA high-throughput sequencing

RA (1 μM) or an equal volume of DMSO was added to mESCs. After 24 h, total RNA was isolated using TRIZOL reagent (LifeTechnologies, Carlsbad, CA, USA). The RNeasy mini kit (Qiagen, GmBH, Germany) was used to further purify total RNA. Total RNA samples were analyzed using the Agilent SurePrint G3 Mouse GE8*60K Microarray (Agilent Technologies) by the Shanghai Biochip Company (Shanghai, China). Microarray data were deposited in the NCBI GEO database (http://www.ncbi.nlm.nih.gov/geo/query/acc.cgi?acc=GSE43405).

miRNA samples were isolated using the mirVana miRNA Isolation Kit (Ambion, Austin, TX, USA). The obtained 50 nt transcripts were subjected to high-throughput sequencing. Clean sequences were obtained after the removal of joints, lower-quality readings, and contamination and then analyzed as described. Small-RNA sequencing data were deposited in the NCBI database (http://www.ncbi.nlm.nih.gov/geo/query/acc.cgi?acc=GSE54145).

### GO annotation and KEGG analysis

GO annotation and KEGG analysis were performed using the Database for Annotation, Visualization and Integrated Discovery Bioinformatics Resources (DAVID) (http://david.abcc.ncifcrf.gov/). We selected GO terms using an FDR threshold of ≤0.05 to reduce the expected proportion of false positives for all terms. The p value represents the significance of genes in a particular GO term. Assuming that the total number of genes and differentially expressed genes are consistent, the more a gene fell in a particular GO term, the smaller the p value and the more significant the event. KEGG analysis was conducted with a count threshold ≥4 and EASE <0.05. A p value of ≤0.05 indicated strong enrichment in the annotation categories.

### Reverse transcription PCR (RT-PCR) and quantitative real-time PCR (qPCR)

Total RNA was isolated from J1 mESCs using Trizol and reverse transcribed using a SYBR PrimeScript RT-PCR Kit (TaKaRa, Dalian, China). miRNA samples were isolated using the mirVana miRNA Isolation Kit and reverse transcribed using a miScript II RT Kit (Qiagen, Hilden, Germany). RNA was reverse transcribed using a SYBR PrimeScript RT-PCR Kit. Quantitative PCR was performed on an ABI StepOnePlus PCR system (Applied Biosystems, CA, USA), and the results were normalized to GAPDH mRNA levels. Data are expressed as FC = 2 − ΔΔCt. The primer sequences used for qPCR are shown in S5 and S6 Tables.

### Western blot analysis

For western blot analysis, mESCs were lysed with RIPA buffer. The protein concentration was measured by the bicinchoninic acid (BCA) method and proteins were separated on 8%–12% polyacrylamide gels. Immunoblotted proteins were visualized by autography using SuperSignal West Pico Chemiluminescent substrate (Thermo Scientific, IL, USA). Western blotting was performed using the listed primary antibodies.

### Alkaline phosphatase staining

Mouse ESCs were treated with RA or DMSO for 24 h and fixed with 4% paraformaldehyde in PBS for 15 min. The alkaline phosphatase activity of cells was detected with the BCIP/NBT alkaline phosphatase color development kit (Beyotime Institute of Biotechnology).

### Statistical analysis

Data are reported as the mean ± standard error of the mean (SEM) and were analyzed by two-tailed Student’s t-test for pair-wise comparisons or analysis of variance (ANOVA) for multiple comparisons. A p value of <0.05 was considered significant.

## Supporting Information

S1 Fig Western blot analysis of Sox2 and Klf4 with miR-200b/c transfected and RA treatment. The relative levels of Sox2 (A) and Klf4 (B) detected by western blot after miR-200b and miR-200c expression vectors into J1 ES cells for 24 h and treatment with RA for an additional 24 h.
(TIF)

S1 Table Significantly downregulated gene expression in RA-treated J1 mESCs (FC > 2, p < 0.01). Fold change values were provided in comparison with J1 mESCs treated by DMSO.
(DOC)

S2 Table Significantly upregulated gene expression in RA-treated J1 mESCs (FC > 2, p < 0.01). Fold change values were provided in comparison with J1 mESCs treated by DMSO.
(DOC)

S3 Table Significantly downregulated miRNA expression in RA-treated J1 mESCs (FC < 0.4, p < 0.01). Fold change values were provided in comparison with J1 mESCs treated by DMSO standard.
(DOC)

S4 Table Significantly upregulated miRNA expression in RA-treated J1 mESCs (FC > 1.5, p < 0.01). Fold change values were provided in comparison with J1 mESCs treated by DMSO-std.
(DOC)

S5 Table Real-time PCR primers. All primers used for detecting mRNA expression levels by real-time PCR.
(DOC)

S6 Table miRNA real-time PCR primers. List of all primers used for detecting miRNA expression levels by real-time PCR.
(DOC)

S7 Table Sequences of miR-200b and miR-200c primers. All primers used for plasmid construction.
(DOC)

S8 Table HDACs expression level in J1 mESCs during RA treatment for 24 h. Fold change values were provided in comparison with J1 mESCs treated by DMSO. A p value was calculated by three individual samples.
(DOC)
