# Supplementary figures and images for: Retinoic Acid Induces Embryonic Stem Cell Differentiation by Altering Both Encoding RNA and microRNA Expression
Source: PLoS One. 2015 Jul 10;10(7):e0132566. doi: 10.1371/journal.pone.0132566 (PMC4498831; doi:10.1371/journal.pone.0132566)

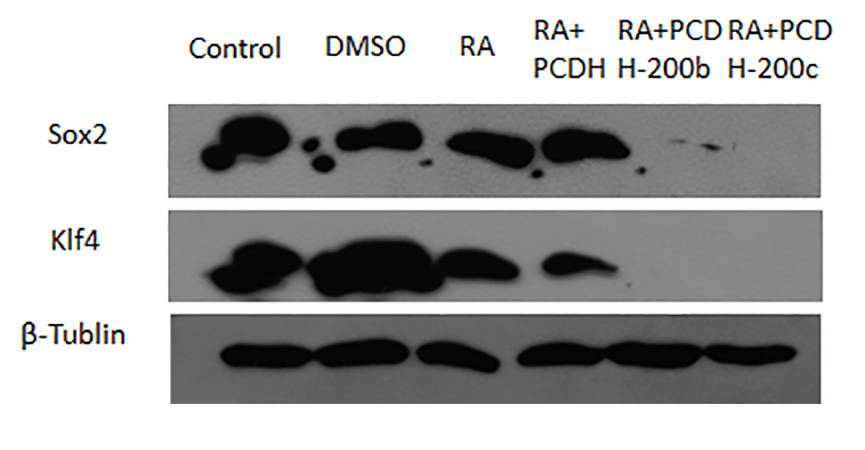

Supplement: S1 Fig — The relative levels of Sox2 (A) and Klf4 (B) detected by western blot after miR-200b and miR-200c expression vectors into J1 ES cells for 24 h and treatment with RA for an additional 24 h. (TIF) [file pone.0132566.s001.tif]
