# Supplementary material for: Retinoic Acid Induces Embryonic Stem Cell Differentiation by Altering Both Encoding RNA and microRNA Expression
Source: PLoS One. 2015 Jul 10;10(7):e0132566. doi: 10.1371/journal.pone.0132566 (PMC4498831; doi:10.1371/journal.pone.0132566)
Supplement: S1 Table — Fold change values were provided in comparison with J1 mESCs treated by DMSO. (DOC) [file pone.0132566.s002.doc]

**Table S1 Significantly down-regulated gene in RA treated J1 mESCs. (Fold Change>2, P-value<0.01)**

Fold change values were provided in comparison with J1 mESCs treated by DMSO.

| Symbol | pvalues | foldchange |
| --- | --- | --- |
| 1110002N22Rik | 0.0074 | 0.3492 |
| 1110008J03Rik | 0.0012 | 0.3452 |
| 1110032A13Rik | 0.0058 | 0.3944 |
| 1110035H17Rik | 0.0036 | 0.3594 |
| 1190002A17Rik | 0.0048 | 0.398 |
| 1190002H23Rik | 0.0021 | 0.0987 |
| 1190005I06Rik | 0.0024 | 0.2815 |
| 1300017J02Rik | 0.0001 | 0.2549 |
| 1500041N16Rik | 0.0026 | 0.4075 |
| 1600029D21Rik | 0.0060 0.0035 | 0.3061 0.2204 |
| 1700001L05Rik | 0 | 0.1192 |
| 1700001P01Rik | 0.0036 | 0.0845 |
| 1700012A16Rik | 0.0016 | 0.4816 |
| 1700013B16Rik | 0 | 0.4511 |
| 1700016D06Rik | 0.0001 | 0.0577 |
| 1700016M24Rik | 0.0004 | 0.0776 |
| 1700019A02Rik | 0.0018 | 0.1465 |
| 1700019D03Rik | 0.0008 | 0.0286 |
| 1700021C14Rik | 0.0069 | 0.3261 |
| 1700025K23Rik | 0.0005 | 0.2961 |
| 1700026D08Rik | 0.0055 | 0.0954 |
| 1700029P11Rik | 0.0009 | 0.3402 |
| 1700030C10Rik | 0.001 | 0.3583 |
| 1700049L16Rik | 0.0001 | 0.479 |
| 1700063H04Rik | 0.002 | 0.0741 |
| 1700067C01Rik | 0.0001 | 0.2297 |
| 1700102P08Rik | 0 | 0.1167 |
| 1700112C13Rik | 0.0077 | 0.1682 |
| 1700123K08Rik | 0.0025 | 0.2227 |
| 1810005K13Rik | 0 | 0.3022 |
| 1810014B01Rik | 0.0074 0.0029 | 0.4375 0.4571 |
| 1810014F10Rik | 0.0006 | 0.235 |
| 2010001M09Rik | 0.0033 | 0.2519 |
| 2010011I20Rik | 0.0096 | 0.4709 |
| 2010107G23Rik | 0.0001 | 0.4616 |
| 2010111I01Rik | 0 | 0.3664 |
| 2010305A19Rik | 0.0016 | 0.4718 |
| 2200001I15Rik | 0.0007 | 0.1132 |
| 2310005G13Rik | 0.0029 | 0.2231 |
| 2310010J17Rik | 0.0014 | 0.4256 |
| 2310044G17Rik | 0.0072 | 0.325 |
| 2310079F23Rik | 0.0002 | 0.4002 |
| 2310079N02Rik | 0 | 0.32 |
| 2410004A20Rik | 0.0001 | 0.0985 |
| 2410076I21Rik | 0.0003 | 0.3234 |
| 2410124H12Rik | 0.004 | 0.3197 |
| 2410137M14Rik | 0.01 | 0.4543 |
| 2610002D18Rik | 0.0025 | 0.445 |
| 2610019F03Rik | 0.005 | 0.253 |
| 2610036L11Rik | 0.0019 | 0.4013 |
| 2610101N10Rik | 0.0049 | 0.2753 |
| 2610301F02Rik | 0.0037 0.0010 | 0.3861 0.179 |
| 2700049P18Rik | 0 | 0.3304 |
| 2810429I04Rik | 0.0003 | 0.333 |
| 2810433K01Rik | 0.0001 | 0.456 |
| 2810453I06Rik | 0.0001 | 0.388 |
| 2900011O08Rik | 0.0025 0.0028 | 0.1074 0.0673 |
| 3110001I22Rik | 0.0001 | 0.3254 |
| 3830431G21Rik | 0 | 0.39 |
| 4732444A12Rik | 0 | 0.0724 |
| 4930420K17Rik | 0.0099 | 0.4884 |
| 4930434E21Rik | 0.0061 | 0.1254 |
| 4930486L24Rik | 0.0033 | 0.1987 |
| 4930539E08Rik | 0.0038 | 0.2239 |
| 4930572J05Rik | 0.0021 | 0.4281 |
| 4931407G18Rik | 0.0011 | 0.0356 |
| 4931417G12Rik | 0.0003 | 0.0892 |
| 4931440B09Rik | 0.0009 | 0.3823 |
| 4931440L10Rik | 0.0091 | 0.2822 |
| 4932417I16Rik | 0 | 0.1998 |
| 4932425I24Rik | 0.004 | 0.1405 |
| 4932441K18Rik | 0.0001 | 0.1827 |
| 4933402E13Rik | 0.0019 | 0.2976 |
| 4933437F05Rik | 0.001 | 0.4256 |
| 5730416F02Rik | 0.0077 | 0.3173 |
| 5830410O09Rik | 0.001 | 0.1573 |
| 5830416P10Rik | 0.0015 | 0.2549 |
| 6030422M02Rik | 0.0002 | 0.1733 |
| 6330407J23Rik | 0.0029 | 0.4267 |
| 6330578E17Rik | 0.0039 | 0.395 |
| 6720457D02Rik | 0.0036 | 0.2904 |
| 8030462N17Rik | 0.0033 | 0.4245 |
| 9030409G11Rik | 4.0E-4 0.0014 | 0.2231 0.4828 |
| 9030425E11Rik | 0.0021 | 0.4595 |
| 9030607L17Rik | 0.0002 | 0.4188 |
| 9030624J02Rik | 0 | 0.4632 |
| 9130017N09Rik | 0.0026 | 0.4834 |
| 9130221D24Rik | 0.0065 | 0.3746 |
| 9330129D05Rik | 0.0002 | 0.4636 |
| 9530058B02Rik | 0.0046 | 0.4839 |
| 9630025I21Rik | 0.0027 | 0.2928 |
| 9930012K11Rik | 0.0028 | 0.2954 |
| A230050P20Rik | 0.0003 | 0.2523 |
| A330084C13Rik | 0.0029 | 0.2919 |
| A530054K11Rik | 0.0031 | 0.3099 |
| A630055G03Rik | 0.0001 | 0.3544 |
| A830018L16Rik | 0 | 0.0745 |
| AA987161 | 0.0016 | 0.3107 |
| Aasdhppt | 0 | 0.4883 |
| Aass | 0.0004 | 0.0474 |
| Abcb1b | 0.0098 | 0.4579 |
| Abcb4 | 0.0047 | 0.4255 |
| Abcc2 | 0.0034 | 0.1906 |
| Abcc4 | 0.0001 | 0.2318 |
| Abcg1 | 0.003 | 0.4087 |
| Abhd14a | 0.0084 | 0.3565 |
| Abi3 | 0.0002 | 0.082 |
| Abp1 | 0.007 | 0.2 |
| Acadl | 0.0059 | 0.4933 |
| Acadm | 0 | 0.3417 |
| Acer1 | 0 | 0.1347 |
| Acot10 | 0.0078 | 0.1651 |
| Acot9 | 0.0015 | 0.2918 |
| Acp6 | 0 | 0.135 |
| Acrbp | 0.0064 | 0.423 |
| Acsl1 | 0.0001 | 0.4921 |
| Acss1 | 0.0001 | 0.0517 |
| Actn3 | 0 | 0.1791 |
| Acy1 | 0.0029 | 0.2831 |
| Adam23 | 0.0004 | 0.1937 |
| Adhfe1 | 0 | 0.0597 |
| Adm | 0.0012 | 0.2126 |
| Agmat | 0 | 0.4271 |
| Agtrap | 0.003 | 0.29 |
| Ahsg | 0.0001 | 0.1535 |
| AI467606 | 0.0008 | 0.1157 |
| AI847670 | 0.0048 | 0.2301 |
| AI854517 | 0.004 | 0.4196 |
| Ak7 | 0.0039 | 0.0195 |
| Akr1b8 | 0.0015 | 0.3499 |
| Aldh1b1 | 0.0003 | 0.2605 |
| Aldh1l2 | 0.0068 | 0.1481 |
| Aldh2 | 0.0064 | 0.2033 |
| Alox12 | 0.0055 | 0.3723 |
| Alpk3 | 0 | 0.1128 |
| Alpl | 0.0006 | 0.2041 |
| Amhr2 | 0.0015 | 0.2693 |
| Ampd3 | 0.0014 | 0.059 |
| Amz1 | 0.0004 | 0.3449 |
| Anapc7 | 0.0095 | 0.2626 |
| Angel1 | 0 | 0.4732 |
| Angptl4 | 0.0001 | 0.2436 |
| Ank3 | 0.003 | 0.4318 |
| Ankmy2 | 0.0 2.0E-4 | 0.4947 0.3134 |
| Ankrd27 | 0.0002 | 0.3305 |
| Ankrd35 | 0.0003 | 0.0958 |
| Ankrd37 | 0.0048 | 0.1687 |
| Anxa10 | 0.0042 | 0.3032 |
| Anxa7 | 0.0084 | 0.4203 |
| Anxa8 | 0.0032 | 0.1485 |
| Aoc3 | 0.002 | 0.2213 |
| Ap1m2 | 0.0004 | 0.2127 |
| Apobec3 | 0.0001 | 0.1877 |
| Apoc2 | 0 | 0.1588 |
| Apoe | 0.0016 | 0.4714 |
| Apof | 0.0003 | 0.2993 |
| Aqp3 | 0.0001 | 0.196 |
| Arfgef2 | 0.0053 | 0.4188 |
| Arhgap4 | 0.0008 | 0.3857 |
| Arhgef18 | 0.0033 | 0.3947 |
| Arid5b | 0.0003 | 0.3842 |
| Arl15 | 0.0001 | 0.4866 |
| Arl5c | 0.0015 | 0.4855 |
| Armc9 | 0.0002 | 0.4361 |
| Asb1 | 0.0039 | 0.4218 |
| Asl | 0.0004 | 0.4665 |
| Asna1 | 0.0047 | 0.3367 |
| Atad4 | 0.0009 | 0.2362 |
| Atf1 | 0.0038 | 0.43 |
| Atf7ip2 | 0.0027 | 0.1968 |
| Atg2a | 0.0001 | 0.3889 |
| Atl3 | 0.0007 | 0.4927 |
| Atp10d | 0.0001 | 0.3416 |
| Atp1b1 | 0.0003 | 0.4526 |
| Atp5s | 0 | 0.4407 |
| Atp6v1b2 | 0.0081 | 0.4737 |
| Atp6v1h | 0.0086 | 0.4714 |
| Atxn3 | 0.0001 | 0.3627 |
| AU018091 | 0.0096 | 0.1142 |
| AW549877 | 0.0037 | 0.4455 |
| Azin1 | 0 | 0.3037 |
| B230311B06Rik | 0 | 0.1233 |
| B230315N10Rik | 0.0028 | 0.4496 |
| B3gnt7 | 0.0085 | 0.3239 |
| Bard1 | 0 | 0.2411 |
| Baz2a | 0.0048 | 0.4559 |
| Bbs5 | 0.0085 | 0.4202 |
| BC002059 | 0.0029 | 0.3605 |
| BC016495 | 0.007 | 0.3465 |
| BC021614 | 0.0032 | 0.144 |
| BC024814 | 0 | 0.4248 |
| BC026762 | 0.002 | 0.4451 |
| BC031781 | 0.0069 | 0.4904 |
| BC032203 | 2.0E-4 0.0095 | 0.1091 0.0315 |
| BC048403 | 0.0003 | 0.468 |
| BC050777 | 0.0028 | 0.3129 |
| BC057170 | 0.0002 | 0.3956 |
| BC063263 | 0.0002 | 0.2566 |
| BC088983 | 0.0068 | 0.3469 |
| Bccip | 0 | 0.4934 |
| Bcl11b | 0.0084 0.0058 | 0.0374 0.151 |
| Bcl2l10 | 0.0004 | 0.0875 |
| Bdh1 | 0.0001 | 0.1678 |
| Bhlha15 | 0.0003 | 0.3242 |
| Bhlhe40 | 0.0001 | 0.1328 |
| Bik | 0.0001 | 0.4411 |
| Bst1 | 0.0002 | 0.196 |
| Bxdc1 | 0.0005 | 0.4474 |
| Bzw2 | 0 | 0.4039 |
| C030048B08Rik | 0.0002 | 0.3765 |
| C230055K05Rik | 0.0001 | 0.4785 |
| C330006D17Rik | 0.0001 | 0.4188 |
| C330011K17Rik | 0.0054 | 0.3398 |
| C79127 | 0 | 0.2169 |
| C79407 | 0.0087 | 0.4939 |
| C8a | 0 | 0.2008 |
| Cabc1 | 0.0059 | 0.3901 |
| Calca | 0.0013 | 0.0459 |
| Calcr | 0.0013 | 0.4037 |
| Calhm2 | 0.008 | 0.4384 |
| Calml4 | 0.0008 | 0.2816 |
| Camk1d | 0.0091 | 0.0548 |
| Capn1 | 0.0034 | 0.3995 |
| Capsl | 0.0001 | 0.0532 |
| Car2 | 0.0011 | 0.4684 |
| Card11 | 0.002 | 0.3086 |
| Cars2 | 2.0E-4 4.0E-4 | 0.4543 0.4461 |
| Casq2 | 0.0008 | 0.1176 |
| Cblc | 0.0074 | 0.3408 |
| Cbr3 | 0.0005 | 0.3937 |
| Cby1 | 0.0085 | 0.4702 |
| Ccbe1 | 0.0088 | 0.2053 |
| Ccdc116 | 0 | 0.2307 |
| Ccdc13 | 0.0012 | 0.4563 |
| Ccdc43 | 0.0042 | 0.4094 |
| Ccdc44 | 0.0045 | 0.4856 |
| Ccdc79 | 0.0023 | 0.2783 |
| Ccdc90a | 0.0001 | 0.3202 |
| Ccno | 0.0005 | 0.388 |
| Ccrl1 | 0.0068 | 0.203 |
| Ccs | 0 | 0.453 |
| Cd3d | 0.0045 | 0.1507 |
| Cd79b | 0.0024 | 0.2534 |
| Cd9 | 0.0007 | 0.4796 |
| Cd96 | 0.0006 | 0.0833 |
| Cdc37l1 | 0.0015 | 0.4057 |
| Cdc42bpg | 0.0074 | 0.3599 |
| Cdc42ep2 | 0.0001 | 0.3153 |
| Cdc42ep3 | 0.0004 | 0.3656 |
| Cdh1 | 0.0002 | 0.2472 |
| Cdh4 | 0.0001 | 0.2276 |
| Cdkl2 | 0 | 0.326 |
| Cdyl | 0.0045 | 0.2525 |
| Ceacam10 | 0.0059 | 0.2255 |
| Ceacam18 | 0.0033 | 0.2279 |
| Cenpm | 0 | 0.3295 |
| Cep110 | 0.0009 | 0.4681 |
| Chac1 | 0.0027 | 0.1422 |
| Chchd10 | 0 | 0.0758 |
| Chchd4 | 0.0003 | 0.3957 |
| Chd2 | 0.0013 | 0.4814 |
| Chek2 | 0.0071 | 0.3321 |
| Chga | 0 | 0.3023 |
| Chmp4c | 0.0017 | 0.0913 |
| Chn1 | 0.0002 | 0.4185 |
| Chrna9 | 0.0004 | 0.0068 |
| Chtf18 | 0.0061 | 0.4608 |
| Cidea | 0 | 0.2275 |
| Cited4 | 0.0001 | 0.4416 |
| Ckb | 0.0014 | 0.2955 |
| Clca2 | 0.0046 | 0.1449 |
| Cldn5 | 0 | 0.1345 |
| Clec2i | 0.0057 | 0.198 |
| Clnk | 0.0035 | 0.1805 |
| Clybl | 0.0022 | 0.374 |
| Cndp2 | 0 | 0.3894 |
| Cnnm1 | 0.0019 | 0.279 |
| Col29a1 | 0.0054 | 0.0226 |
| Col5a3 | 0.0032 | 0.2227 |
| Col9a2 | 0 | 0.2466 |
| Comtd1 | 0.0036 | 0.412 |
| Cox6b2 | 0 | 0.3177 |
| Cox7b2 | 0 | 0.2405 |
| Cplx1 | 0.0003 | 0.1738 |
| Cpn1 | 0.0061 | 0.3334 |
| Cpne5 | 0 | 0.1937 |
| Cpne9 | 0 | 0.0468 |
| Cpt1a | 0.0009 | 0.0448 |
| Creld1 | 0.0041 | 0.3204 |
| Crisp1 | 0 | 0.0137 |
| Cry1 | 0.0018 | 0.1733 |
| Cryba1 | 0 | 0.3346 |
| Csad | 0.0015 | 0.3013 |
| Csrp2 | 0 | 0.2044 |
| Ctbp2 | 0.0002 | 0.3566 |
| Cth | 0.0012 | 0.0701 |
| Ctxn3 | 0.0002 | 0.1914 |
| Cxcl12 | 0.0007 | 0.4687 |
| Cyb5b | 0.001 | 0.2558 |
| Cyb5r1 | 0.0 0.0013 | 0.3529 0.1853 |
| Cyp11a1 | 0 | 0.0291 |
| Cyp1a1 | 0.0007 | 0.2076 |
| Cyp2c29 | 0.0046 | 0.2121 |
| Cyp2c44 | 0.0097 | 0.1751 |
| Cyp4f14 | 0.0003 | 0.0544 |
| Cysltr2 | 0.0074 | 0.2246 |
| D10Ertd322e | 0.0019 | 0.4539 |
| D14Ertd668e | 0.0063 | 0.3379 |
| D16Ertd472e | 0.0022 0.0056 | 0.3488 0.2335 |
| D19Ertd652e | 0.0023 0.0049 | 0.0951 0.24 |
| D230025D16Rik | 0.0006 | 0.441 |
| D3Ertd300e | 0.0055 5.0E-4 | 0.4573 0.4282 |
| D4Bwg0951e | 0.0034 | 0.4531 |
| D630032N06Rik | 0.0003 | 0.3403 |
| D6Wsu163e | 0 | 0.4402 |
| D930028M14Rik | 0.0002 | 0.3685 |
| Dab1 | 9.0E-4 0.0080 | 0.109 0.3522 |
| Dapk2 | 0.0048 | 0.3145 |
| Dbf4 | 0.007 | 0.4413 |
| Dbil5 | 0.0082 | 0.4645 |
| Dbt | 0.0094 | 0.4025 |
| Dcdc2a | 0.001 | 0.0654 |
| Dci | 0 | 0.3474 |
| Dclre1c | 0.0079 | 0.4715 |
| Dctd | 5.0E-4 0.0056 | 0.2637 0.18 |
| Ddx10 | 0.0001 | 0.4801 |
| Ddx11 | 0.0004 | 0.4858 |
| Dedd | 0.0046 | 0.481 |
| Defa23 | 0 | 0.2086 |
| Dennd1c | 0.0004 | 0.2984 |
| Dennd2c | 0.0011 | 0.0883 |
| Dennd4a | 0 | 0.3152 |
| Depdc6 | 0.0057 | 0.2041 |
| Depdc7 | 0.008 | 0.4925 |
| Derl3 | 0.0013 | 0.2587 |
| Dgka | 0.0034 | 0.2682 |
| Dgke | 0.0008 | 0.3061 |
| Dhrs11 | 0.0028 | 0.3521 |
| Dhrs4 | 0.0025 | 0.442 |
| Dhrs7 | 0 | 0.4765 |
| Dlgap3 | 0 | 0.1752 |
| Dmc1 | 0.0031 | 0.1797 |
| Dmrtc2 | 0.0024 | 0.1081 |
| Dnajb6 | 3.0E-4 0.0042 | 0.4024 0.47 |
| Dnajc21 | 0 | 0.351 |
| Dnmt3l | 0.001 | 0.2106 |
| Dock2 | 0.0005 | 0.1645 |
| Dock5 | 0.0002 | 0.4188 |
| Dok2 | 0 | 0.2258 |
| Dpf1 | 0.0001 | 0.1872 |
| Dph1 | 0.0092 | 0.2737 |
| Dpp4 | 0 | 0.1827 |
| Dpy30 | 0.0027 | 0.3905 |
| Dpys | 0.0027 | 0.0323 |
| Dscaml1 | 0.0048 | 0.4681 |
| Dtx1 | 0.0004 | 0.2583 |
| Dusp27 | 0 | 0.0946 |
| E030010N08Rik | 0.0006 | 0.0333 |
| E130016E03Rik | 0 | 0.4989 |
| E2f2 | 0.0003 | 0.4234 |
| Ebi3 | 0.0002 | 0.2374 |
| Ecsit | 0.0029 | 0.3625 |
| Eef2k | 0.0004 | 0.4215 |
| Efcab10 | 0.0049 | 0.077 |
| Efcab2 | 0.0005 | 0.3633 |
| Efhb | 0.0039 | 0.0498 |
| Efhc2 | 0.0067 | 0.3812 |
| EG333452 | 0.0005 | 0.1754 |
| EG333669 | 0.0 0.0038 | 0.3871 0.0804 |
| EG380907 | 0.0021 | 0.2296 |
| EG382161 | 0.0004 | 0.2534 |
| EG433225 | 0.0014 | 0.4041 |
| EG433326 | 0.003 | 0.3236 |
| EG434121 | 0.003 | 0.2945 |
| EG627821 | 0 | 0.0268 |
| EG633250 | 0 | 0.4038 |
| EG668831 | 0.0002 | 0.4593 |
| Egln3 | 0.0016 | 0.1298 |
| Egr4 | 0.0009 | 0.1001 |
| Eif2s2 | 0.0071 | 0.473 |
| Eif4a2 | 0.0024 | 0.3573 |
| Elavl3 | 0.0002 | 0.1541 |
| En2 | 0.0033 | 0.4209 |
| Enc1 | 0.0004 | 0.1683 |
| Enpp3 | 0.0001 | 0.1503 |
| ENSMUSG00000052976 | 0.002 | 0.4513 |
| ENSMUSG00000053338 | 0.0001 | 0.1128 |
| ENSMUSG00000057802 | 0.0013 | 0.0838 |
| ENSMUSG00000074303 | 0.0032 | 0.2784 |
| Ep400 | 0.0029 | 0.3562 |
| Epas1 | 0.0018 | 0.1448 |
| Epb4.1l5 | 0.0014 | 0.2548 |
| Epcam | 0.001 | 0.1587 |
| Epha1 | 0.0005 | 0.171 |
| Epha2 | 0.0008 | 0.0609 |
| Eras | 0.0021 | 0.0419 |
| Ercc8 | 0.0054 | 0.4093 |
| Esco2 | 0.0031 | 0.208 |
| Esrrb | 0.0053 | 0.0204 |
| Etv2 | 0.0014 | 0.2346 |
| Exosc5 | 0 | 0.4584 |
| Exph5 | 0.0055 | 0.1949 |
| Extl1 | 0.0075 | 0.1762 |
| Faah | 0.0001 | 0.3553 |
| Fah | 0 | 0.1463 |
| Fam110b | 0.0004 | 0.3973 |
| Fam169a | 0.0005 | 0.2935 |
| Fam175a | 0.0005 | 0.4512 |
| Fam176a | 0.0046 | 0.2809 |
| Fam19a4 | 0.0019 | 0.0524 |
| Fam20a | 0.0079 | 0.4256 |
| Fam26f | 0.0012 | 0.282 |
| Fam49a | 0.0039 | 0.4377 |
| Fam62c | 0.0084 | 0.3431 |
| Fam78a | 0 | 0.1721 |
| Fank1 | 0 | 0.2512 |
| Farsa | 0.0008 | 0.3546 |
| Fbxo15 | 0.0001 | 0.2426 |
| Fbxo5 | 0 | 0.4465 |
| Fcgr2b | 0 | 0.0222 |
| Fcgr3 | 0 | 0.1468 |
| Fdxr | 0.0001 | 0.4915 |
| Fgd1 | 0.0068 | 0.2737 |
| Fgf15 | 0.0001 | 0.1388 |
| Fgf17 | 0 | 0.0474 |
| Fgr | 0.0051 | 0.1123 |
| Fhad1 | 0.001 | 0.4584 |
| Fig4 | 0.0054 | 0.3101 |
| Fitm2 | 0.0005 | 0.3235 |
| Fiz1 | 0.0012 | 0.4888 |
| Fkbp11 | 0.0003 | 0.3085 |
| Fntb | 0.0002 | 0.3938 |
| Foxo1 | 0.0048 1.0E-4 | 0.4174 0.4213 |
| Foxred1 | 0.0024 | 0.4869 |
| Ftsj1 | 0.0026 | 0.4195 |
| Ftsj2 | 0.0045 | 0.3127 |
| Ftsj3 | 0.0008 | 0.4352 |
| Fxyd4 | 0 | 0.0103 |
| Fzd5 | 0.0027 | 0.4023 |
| Fzd9 | 0.0024 | 0.3886 |
| G3bp2 | 0.0063 0.0038 | 0.4272 0.3523 |
| Gabbr1 | 0.0034 | 0.4441 |
| Gabpa | 0.0021 | 0.3586 |
| Gabpb1 | 0.0004 | 0.453 |
| Galk1 | 0.0028 | 0.4462 |
| Galnt9 | 0.0046 | 0.1039 |
| Galr2 | 0.0052 | 0.1535 |
| Gart | 0.0016 0.0 | 0.3746 0.2159 |
| Gas5 | 0.0001 | 0.3948 |
| Gatsl3 | 0.0078 | 0.1882 |
| Gca | 0 | 0.2116 |
| Gcnt2 | 0.01 | 0.2912 |
| Gdpd3 | 0.0005 | 0.3656 |
| Gfod1 | 0.0012 | 0.1621 |
| Ggt6 | 0.007 | 0.293 |
| Gipc2 | 0.0004 | 0.0904 |
| Gipr | 0 | 0.2209 |
| Glb1 | 0 | 0.3239 |
| Glod5 | 0.0001 | 0.0248 |
| Gls2 | 0.0001 | 0.3679 |
| Glyctk | 0.0067 | 0.3141 |
| Gm1060 | 0.0011 | 0.3964 |
| Gm129 | 0.0023 | 0.1211 |
| Gm1631 | 0 | 0.0272 |
| Gm2a | 0.0038 | 0.3832 |
| Gnl3 | 0.0054 | 0.4701 |
| Gpa33 | 0 | 0.0406 |
| Gpd1l | 0.0001 | 0.4174 |
| Gpd2 | 0.009 | 0.4968 |
| Gpr133 | 0 | 0.0686 |
| Gpr135 | 0.0002 | 0.3135 |
| Gpr182 | 0.0018 | 0.361 |
| Gpr4 | 0.0014 | 0.4108 |
| Gpt2 | 0.0008 | 0.4551 |
| Gpx4 | 0.0059 | 0.4528 |
| Gpx6 | 0.0005 | 0.3449 |
| Grap2 | 0.0017 | 0.3341 |
| Grb7 | 0.0007 | 0.4736 |
| Grhl2 | 0.004 | 0.122 |
| Grpel2 | 0.007 | 0.4661 |
| Grrp1 | 0.0063 | 0.3559 |
| Grtp1 | 0 | 0.3698 |
| Gsta3 | 0 | 0.1739 |
| Gsta4 | 0 | 0.2802 |
| Gstm7 | 0.0035 | 0.336 |
| Gstt4 | 0.0009 | 0.3398 |
| Gtsf1 | 0 | 0.1833 |
| Gtsf1l | 0.0003 | 0.0205 |
| H2-DMa | 0.0032 | 0.2656 |
| H2-DMb1 | 0.0002 | 0.3238 |
| Hal | 0.0001 | 0.0453 |
| Harbi1 | 0.003 | 0.4336 |
| Haus2 | 0 | 0.2307 |
| Hbp1 | 0.0011 | 0.4861 |
| Hbq1 | 0.0003 | 0.1946 |
| Hdac2 | 0.0011 | 0.445 |
| Hdac8 | 0.0010 2.0E-4 | 0.4084 0.463 |
| Heatr2 | 0 | 0.4347 |
| Helb | 0 | 0.2866 |
| Hells | 0.0004 | 0.4542 |
| Hes2 | 0.0073 | 0.4749 |
| Hexb | 0.0006 | 0.3728 |
| Hirip3 | 0 | 0.4897 |
| Hk2 | 0.0002 | 0.2328 |
| Hltf | 0.0023 0.0024 | 0.2207 0.2544 |
| Hopx | 0.0002 | 0.4306 |
| Hormad1 | 0.0019 | 0.0566 |
| Hpdl | 0.0009 | 0.1526 |
| Hprt1 | 0.0001 | 0.3794 |
| Hps3 | 0.005 | 0.3407 |
| Hrh3 | 0.0013 | 0.1855 |
| Hs1bp3 | 0.0037 | 0.3967 |
| Hsd17b1 | 0.0004 | 0.2946 |
| Hsd17b11 | 0.0 0.0063 | 0.1103 0.2172 |
| Htatip2 | 0.0043 | 0.3877 |
| Htr5b | 0.0001 | 0.2421 |
| Hus1b | 0 | 0.1928 |
| Icam1 | 0.0044 | 0.139 |
| Icam4 | 0.0011 | 0.3617 |
| Idh3a | 0.0052 | 0.3396 |
| Ier3 | 0.0081 | 0.1489 |
| Ifrd2 | 0.0001 | 0.4881 |
| Igfals | 0.008 | 0.18 |
| Igsf21 | 0.0016 | 0.0858 |
| Il17b | 0.0012 | 0.1831 |
| Il27ra | 0.0041 | 0.1866 |
| Impa2 | 0 | 0.1995 |
| Ing3 | 0.0011 | 0.4623 |
| Ing5 | 0.0001 | 0.4762 |
| Inhbc | 0.0002 | 0.0713 |
| Ino80 | 0.0003 | 0.4322 |
| Ino80c | 0.0001 | 0.3305 |
| Inpp5d | 0 | 0.0866 |
| Insig1 | 0.0012 | 0.3525 |
| Ints12 | 0.006 | 0.491 |
| Intu | 0.0016 | 0.3195 |
| Ipmk | 0.0057 0.0096 | 0.2503 0.3409 |
| Ipo7 | 0.0003 | 0.4566 |
| Iqsec3 | 0.0077 | 0.2841 |
| Irak3 | 0.0005 | 0.106 |
| Irx6 | 0.0003 | 0.2168 |
| Isoc2a | 0.0017 | 0.2412 |
| Isyna1 | 0 | 0.4757 |
| Itgb7 | 0.0003 | 0.0473 |
| Itpk1 | 0.0036 | 0.2355 |
| Itpka | 0 | 0.0526 |
| Itpr3 | 0 | 0.3115 |
| Jag1 | 0.002 | 0.2585 |
| Jak3 | 0.0026 | 0.1416 |
| Jarid2 | 0.0022 | 0.3213 |
| Jph4 | 0.0061 | 0.3338 |
| Kcnb1 | 0.0009 | 0.2038 |
| Kcnip2 | 0.0025 | 0.304 |
| Kcnk5 | 0.0009 | 0.2049 |
| Kcnn2 | 0.007 | 0.4738 |
| Kcns3 | 0.0033 | 0.1983 |
| Kctd3 | 0.0007 | 0.3914 |
| Kctd4 | 0.0046 | 0.3382 |
| Kctd6 | 0.0092 | 0.4696 |
| Kdm5b | 0 | 0.4358 |
| Kif9 | 0.0004 | 0.3912 |
| Kirrel2 | 0.0015 | 0.0059 |
| Klf1 | 0.0006 | 0.2605 |
| Klf12 | 0.0094 | 0.1463 |
| Klf16 | 0.0001 | 0.3989 |
| Klf17 | 0.0078 | 0.3508 |
| Klf2 | 0 | 0.0583 |
| Klf5 | 0 | 0.2345 |
| Klf8 | 0 | 0.1707 |
| Klk1 | 0.0007 | 0.0584 |
| Klk1b3 | 0.0003 | 0.3494 |
| Klk8 | 0 | 0.4033 |
| Kndc1 | 0 | 0.0816 |
| Kntc1 | 0.0033 | 0.4987 |
| L2hgdh | 0.0008 | 0.4242 |
| L3mbtl2 | 0.0041 | 0.3108 |
| l7Rn6 | 0.0003 | 0.4525 |
| Lace1 | 0.0006 | 0.4733 |
| Lap3 | 0.0018 | 0.3266 |
| Laptm5 | 0.0023 4.0E-4 | 0.1085 0.0939 |
| Lars | 0.0001 | 0.4741 |
| Lass4 | 0 | 0.3756 |
| Ldhal6b | 0.002 | 0.2833 |
| Lef1 | 0.0001 | 0.0834 |
| Lefty1 | 0 | 0.0452 |
| Leng9 | 0.0017 | 0.3815 |
| Lgtn | 0 | 0.4758 |
| Lipe | 0.0093 | 0.4391 |
| Liph | 0 | 0.0259 |
| Lipt1 | 0.0023 | 0.3563 |
| Lmcd1 | 0.0033 | 0.2319 |
| Lmln | 0.0017 | 0.4375 |
| LOC100045796 | 0.0017 | 0.2785 |
| LOC100047943 | 0.005 | 0.3108 |
| LOC100048500 | 0.0068 | 0.261 |
| LOC677447 | 0 | 0.2423 |
| Lrch4 | 0.0086 | 0.1931 |
| Lrmp | 0 | 0.071 |
| Lrp11 | 0.0096 | 0.3163 |
| Lrrc15 | 0.002 | 0.2513 |
| Lrrc27 | 0.0021 | 0.2806 |
| Lrrc34 | 0.001 | 0.0349 |
| Lrrn2 | 0.0001 | 0.4051 |
| Lsm11 | 0 | 0.3854 |
| Lsm12 | 0.0006 | 0.4828 |
| Lss | 0.0002 | 0.4041 |
| Ly6g6e | 0.0005 | 0.0181 |
| Lyl1 | 0.0047 | 0.2972 |
| Lypla1 | 0.0002 | 0.3164 |
| Maff | 0.0003 | 0.2903 |
| Mak | 0.0034 | 0.2788 |
| Man2b1 | 0.0001 | 0.4779 |
| Manba | 0 | 0.0642 |
| Map4k1 | 0.0004 | 0.3778 |
| Mapk8ip2 | 0.0004 | 0.3363 |
| Mars2 | 0.006 | 0.2342 |
| Marveld3 | 0.0062 | 0.4832 |
| Mbl2 | 0.0004 | 0.4788 |
| Mcm3ap | 0.0048 | 0.2952 |
| Mdm4 | 0.0003 | 0.4211 |
| Med21 | 0.0013 | 0.4494 |
| Med7 | 0.0092 | 0.4102 |
| Mertk | 0.0088 | 0.3165 |
| Metapl1 | 0.0043 | 0.4575 |
| Mfi2 | 0.0021 | 0.2004 |
| Mgat4a | 0.0078 | 0.4859 |
| Mgst3 | 0.0038 | 0.4521 |
| Mip | 0.0035 | 0.1275 |
| Mkrn1 | 0 | 0.098 |
| Mlf1ip | 0.0037 | 0.3885 |
| Mlh3 | 0 | 0.3498 |
| Mme | 0.0024 | 0.2927 |
| Mmp19 | 0.0013 | 0.32 |
| Mmrn2 | 0.0007 | 0.2216 |
| Moap1 | 0 | 0.4324 |
| Mobp | 0.004 | 0.3582 |
| Mocos | 0 | 0.1684 |
| Morc1 | 0.0004 | 0.0728 |
| Morn1 | 0.0042 | 0.2083 |
| Mospd2 | 0.0058 | 0.4864 |
| Mpv17l | 0.0004 | 0.1352 |
| Mpzl2 | 0.0008 | 0.0638 |
| Mri1 | 0.0015 | 0.4642 |
| Mrpl1 | 0.0001 | 0.3989 |
| Mrpl35 | 0 | 0.2544 |
| Mrpl50 | 0 | 0.4441 |
| Mrps18b | 0.0031 | 0.3711 |
| Mrps31 | 0.0007 | 0.3606 |
| Mrps5 | 0.0095 | 0.3085 |
| Mrs2 | 0.0001 | 0.4475 |
| Msc | 0 | 0.0149 |
| Msh2 | 0.0004 | 0.4883 |
| Msh3 | 0.0038 | 0.4602 |
| Msh6 | 0 | 0.2828 |
| Msrb2 | 0.0001 | 0.2289 |
| Mtap7 | 0.0055 | 0.1453 |
| Mtf2 | 0.0031 | 0.4675 |
| Mtfr1 | 0.0006 | 0.4299 |
| Mthfd1 | 0 | 0.3671 |
| Mthfd2 | 0.002 | 0.3238 |
| Mtl5 | 0.0001 | 0.2028 |
| Mtrf1 | 0.0001 | 0.3663 |
| Mtss1 | 0.0001 | 0.322 |
| Myc | 0.0026 | 0.324 |
| Myh4 | 0.0016 | 0.3487 |
| Myog | 0.0002 | 0.3893 |
| Myom2 | 0.0015 | 0.1682 |
| N4bp1 | 0.0037 | 0.489 |
| Napb | 0.0002 | 0.4104 |
| Naprt1 | 0.0004 | 0.4437 |
| Napsa | 0.0032 | 0.0504 |
| Nars | 0.0051 | 0.3026 |
| Nars2 | 0.0025 | 0.3131 |
| Ncl | 0.0057 6.0E-4 | 0.394 0.499 |
| Ndrg1 | 0.0008 | 0.1075 |
| Ndrg2 | 0 | 0.0483 |
| Necab2 | 0.0001 | 0.4954 |
| Nefl | 0 | 0.178 |
| Neurod1 | 0.0003 | 0.0689 |
| Nf2 | 0.0004 | 0.2679 |
| Nfatc2 | 0.0064 | 0.4372 |
| Nfatc2ip | 0 | 0.1888 |
| Nfrkb | 0.0038 | 0.4818 |
| Nfyb | 0.0063 | 0.4396 |
| Ng23 | 0.0056 | 0.3563 |
| Ngfr | 0.0012 | 0.4022 |
| Nhedc1 | 0.0066 | 0.4152 |
| Nhedc2 | 0.0001 | 0.0938 |
| Nhlrc1 | 0.0001 | 0.2401 |
| Nit2 | 0.0013 | 0.4263 |
| Nkx6-3 | 0.0003 | 0.0229 |
| Nle1 | 0 | 0.46 |
| Nlrx1 | 0.0093 | 0.4164 |
| Nmnat2 | 0.0094 | 0.1421 |
| Nob1 | 0.0002 | 0.3999 |
| Nodal | 0 | 0.1097 |
| Nol6 | 0.0001 | 0.4365 |
| Nop10 | 0 | 0.4593 |
| Nos3 | 0.0007 | 0.176 |
| Notch4 | 0 | 0.0958 |
| Nqo1 | 0.0005 | 0.4977 |
| Nr0b1 | 0.0001 | 0.0085 |
| Nr4a1 | 0.0055 | 0.2776 |
| Nr5a2 | 0 | 0.0267 |
| Nrf1 | 0.0015 | 0.3516 |
| Nrsn1 | 0.0006 | 0.264 |
| Nsun2 | 0.0016 | 0.4972 |
| Nsun5 | 0.0008 | 0.4967 |
| Nthl1 | 0.0008 | 0.4641 |
| Ntrk2 | 0 | 0.2752 |
| Nubp2 | 0.0006 | 0.4736 |
| Nubpl | 0.0009 | 0.4979 |
| Nudcd1 | 0.0037 | 0.4659 |
| Nudt13 | 0.0044 | 0.447 |
| Nudt19 | 0.0003 | 0.4586 |
| Nufip2 | 0.0019 | 0.3127 |
| Nupr1 | 0.0044 | 0.1588 |
| Nvl | 0.0006 | 0.3582 |
| Oas1f | 0.0056 | 0.4183 |
| Olig1 | 0.0002 | 0.0045 |
| Olig2 | 0.0007 | 0.0934 |
| Orc1l | 0.0059 | 0.3417 |
| Orc5l | 0.008 | 0.3626 |
| Orm2 | 0.0056 | 0.3518 |
| Osbpl10 | 0 | 0.3462 |
| Osgep | 0.0012 | 0.4695 |
| Osta | 0.0047 0.0 | 0.3136 0.3177 |
| OTTMUSG00000000712 | 0.0005 | 0.4132 |
| OTTMUSG00000003456 | 0.0093 | 0.4074 |
| OTTMUSG00000005148 | 0.0012 | 0.0783 |
| P2ry6 | 0.0002 | 0.3615 |
| Paqr3 | 0.0051 | 0.4851 |
| Parp1 | 0.0092 | 0.3136 |
| Parp12 | 0.0017 | 0.321 |
| Parp14 | 0.0012 | 0.3496 |
| Parvb | 1.0E-4 3.0E-4 | 0.26 0.316 |
| Patl2 | 0.0076 | 0.2199 |
| Pax4 | 0.0004 | 0.1592 |
| Pcf11 | 0.0079 | 0.3978 |
| Pcgf2 | 0.0002 | 0.497 |
| Pcid2 | 0.0029 | 0.4731 |
| Pcolce2 | 0.0005 | 0.1042 |
| Pdcd11 | 0.0057 | 0.4169 |
| Pdcd2l | 0.0008 | 0.4795 |
| Pde9a | 0.0001 | 0.1201 |
| Pdgfc | 0.0077 | 0.3624 |
| Pdk1 | 0 | 0.0779 |
| Pdk4 | 0.0039 | 0.3456 |
| Pdss1 | 0 | 0.483 |
| Pdyn | 0.0017 | 0.07 |
| Pecr | 0 | 0.3114 |
| Pfas | 0.0002 | 0.3562 |
| Pfkp | 0.0001 | 0.0476 |
| Pfn4 | 0.0059 | 0.4716 |
| Phb | 0.001 | 0.4792 |
| Phc1 | 0 | 0.1133 |
| Phf19 | 0.0055 | 0.4177 |
| Phlda2 | 0.0008 | 0.3724 |
| Phyh | 0.0023 | 0.3511 |
| Pigl | 0.0016 | 0.1883 |
| Pikfyve | 0.0081 | 0.4114 |
| Pim2 | 0.0046 | 0.1271 |
| Pipox | 0.0002 | 0.0668 |
| Pir | 0 | 0.4161 |
| Pitpnc1 | 0.001 | 0.3005 |
| Pitx2 | 0.0001 | 0.4846 |
| Piwil1 | 0 | 0.1045 |
| Pla1a | 0.0007 | 0.2236 |
| Pla2g1b | 0 | 0.1241 |
| Pla2g4e | 0.0042 | 0.3188 |
| Plcg2 | 0.0036 | 0.3495 |
| Pld6 | 0 | 0.4796 |
| Plek2 | 0.0055 | 0.2538 |
| Plekhf2 | 0.0038 | 0.2364 |
| Plod2 | 0.0082 | 0.4604 |
| Pmaip1 | 0.0081 | 0.4473 |
| Pmfbp1 | 0.002 | 0.2379 |
| Pms1 | 0 | 0.4889 |
| Pnma2 | 0.0038 | 0.4776 |
| Pola2 | 0.004 | 0.3713 |
| Polr3gl | 0.0028 | 0.3949 |
| Pop1 | 0.0001 | 0.4979 |
| Pot1b | 0.0031 | 0.4149 |
| Pou2f2 | 0 | 0.1403 |
| Pou5f1 | 0.0059 | 0.0321 |
| Ppat | 0.0 0.0021 | 0.4206 0.3883 |
| Ppef2 | 0.0091 | 0.1565 |
| Ppfibp2 | 0.0006 | 0.1529 |
| Ppic | 0.0006 | 0.422 |
| Ppif | 0.0091 | 0.4088 |
| Ppm1j | 0.0004 | 0.0582 |
| Ppp1r1a | 0.0095 | 0.4761 |
| Ppp2r2d | 0.0034 | 0.4915 |
| Prdm15 | 0.0037 | 0.454 |
| Prelid2 | 0.0009 | 0.339 |
| Prickle3 | 0.0006 | 0.3582 |
| Prkcz | 0 | 0.4323 |
| Prmt8 | 0 | 0.0492 |
| Proc | 0.0016 | 0.1277 |
| Prodh | 0.0001 | 0.4249 |
| Prok2 | 0.006 | 0.1572 |
| Prps2 | 0.0034 | 0.3007 |
| Prrg4 | 0.0003 | 0.4155 |
| Ptch2 | 0.0062 | 0.0886 |
| Ptk2b | 0 | 0.4246 |
| Ptpn6 | 0.0093 | 0.4385 |
| Ptpn7 | 0.002 | 0.3145 |
| Ptprv | 7.0E-4 0.0022 | 0.1848 0.2312 |
| Ptrf | 0.0011 | 0.4662 |
| Ptrh1 | 0.0023 | 0.4651 |
| Pusl1 | 0.0005 | 0.4797 |
| Pwp1 | 0.0042 | 0.3867 |
| Pycard | 0.0004 | 0.3824 |
| Pycr1 | 0.0051 | 0.165 |
| Pycr2 | 0.0009 | 0.3813 |
| Qprt | 0.0012 | 0.3643 |
| Qrsl1 | 0.0001 | 0.461 |
| Qtrt1 | 0.0001 | 0.336 |
| Rab19 | 0.0016 | 0.2783 |
| Rab25 | 0.0003 | 0.1654 |
| Rab27a | 0.0075 | 0.1246 |
| Rab33a | 0.0003 | 0.2864 |
| Rab39 | 0.0054 | 0.3388 |
| Rab4b | 0.0068 | 0.4809 |
| Rac2 | 0 | 0.4525 |
| Rad51l1 | 0.0011 | 0.403 |
| Rad9b | 0.0081 | 0.2476 |
| Radil | 0.0012 | 0.4683 |
| Ranbp17 | 0.0045 | 0.4313 |
| Rapgef3 | 0.0001 | 0.225 |
| Rars | 0.0044 | 0.4418 |
| Rasa4 | 0.0001 | 0.1119 |
| Rasal1 | 0.0013 | 0.3592 |
| Rasgef1a | 0.0003 | 0.1394 |
| Rasgrp4 | 0.0013 0.0048 | 0.2141 0.3264 |
| Rasl10a | 0.0035 | 0.3601 |
| Rbp7 | 0 | 0.3101 |
| Rcbtb1 | 0.0089 | 0.451 |
| Rdm1 | 0.0012 | 0.4701 |
| Reg1 | 0 | 0.1132 |
| Rest | 0.0012 | 0.2097 |
| Rffl | 0.0014 | 0.4553 |
| Rfx2 | 0.0003 | 0.1544 |
| Rgs9bp | 0.0075 | 0.2101 |
| Rhbdl2 | 0.006 | 0.3169 |
| Rhebl1 | 0.0024 | 0.3602 |
| Rhot1 | 0.0087 | 0.4791 |
| Rhov | 0.0026 | 0.243 |
| Rinl | 0 | 0.409 |
| Rlbp1 | 0.0023 | 0.446 |
| Rln3 | 0.0005 | 0.2437 |
| Rmnd5b | 0.0022 | 0.3364 |
| Rnaseh1 | 0.0001 | 0.469 |
| Rnf113a1 | 0.0001 | 0.4877 |
| Rnf125 | 0.0023 | 0.0379 |
| Rnf138 | 0.0099 | 0.4408 |
| Rnmt | 0.0001 | 0.4073 |
| Rnmtl1 | 0 | 0.4897 |
| Rpp25 | 0.0005 | 0.128 |
| Rpp40 | 0.0024 | 0.2338 |
| Rragb | 0.0067 | 0.4746 |
| Rragc | 0.0076 | 0.4891 |
| Rrn3 | 0.0032 | 0.4004 |
| Rrp1b | 0.0074 | 0.3763 |
| Rrp9 | 0.0002 | 0.3157 |
| Rtn2 | 0.0004 | 0.3535 |
| Rundc3a | 0.0002 | 0.2366 |
| Rundc3b | 0.0096 | 0.3378 |
| Ryr1 | 0.0008 | 0.1962 |
| Ryr2 | 0 | 0.1037 |
| S1pr2 | 0.0039 | 0.3317 |
| Sap25 | 0.0019 | 0.1364 |
| Sap30 | 0 | 0.3728 |
| Sbf2 | 0.0051 | 0.4723 |
| Scand3 | 0 | 0.1716 |
| Scgb3a1 | 0 | 0.0455 |
| Scn2b | 0.0007 | 0.3369 |
| Sdcbp2 | 0.0003 | 0.4259 |
| Sec14l4 | 0.0001 | 0.1633 |
| Secisbp2 | 0.0038 | 0.4875 |
| Sema7a | 0.0041 | 0.3118 |
| Sepsecs | 0.0005 | 0.4278 |
| Serpina3c | 0.0008 | 0.0498 |
| Serpinb5 | 0.0005 | 0.0939 |
| Setx | 0.0056 | 0.3753 |
| Sf1 | 0 | 0.4066 |
| Sfrp4 | 0.0048 | 0.0266 |
| Sfrs17b | 0 | 0.3298 |
| Sgol2 | 0.0006 | 0.4141 |
| Sh2d5 | 0.0024 | 0.1941 |
| Sh3gl2 | 0.0052 | 0.1563 |
| Shisa2 | 0.0016 | 0.3502 |
| Shmt1 | 0 | 0.2689 |
| Shmt2 | 0.0021 | 0.4666 |
| Shq1 | 0.0066 | 0.3387 |
| Siae | 0.0045 | 0.4423 |
| Sirt5 | 0 | 0.267 |
| Slc16a10 | 0.0004 | 0.3495 |
| Slc16a6 | 0.0004 | 0.3757 |
| Slc1a5 | 0.0001 | 0.4077 |
| Slc22a14 | 0.0024 | 0.3114 |
| Slc22a7 | 0.0022 | 0.1829 |
| Slc25a13 | 0.0006 | 0.3192 |
| Slc25a15 | 0.0025 | 0.2754 |
| Slc25a20 | 0.0026 | 0.3255 |
| Slc25a26 | 0.0024 | 0.4576 |
| Slc25a32 | 0.0009 | 0.3357 |
| Slc25a33 | 0.0022 0.0011 | 0.2965 0.4359 |
| Slc25a35 | 0.0002 | 0.2271 |
| Slc25a40 | 0.0029 | 0.2671 |
| Slc27a5 | 0.0047 | 0.2093 |
| Slc28a3 | 0.0016 | 0.4636 |
| Slc2a3 | 0.004 | 0.3197 |
| Slc34a3 | 0.0001 | 0.1802 |
| Slc35f3 | 0.0001 | 0.3328 |
| Slc37a1 | 0.0001 | 0.2143 |
| Slc3a2 | 0.0005 | 0.4058 |
| Slc44a4 | 0 | 0.1434 |
| Slc4a1ap | 0.0046 | 0.4857 |
| Slc4a5 | 0.0009 | 0.2986 |
| Slc5a4b | 0.0028 | 0.1842 |
| Slc6a1 | 0.001 | 0.0302 |
| Slc7a7 | 0 | 0.0756 |
| Slco1b2 | 0.0018 | 0.447 |
| Smad7 | 0.0009 | 0.2573 |
| Smarcad1 | 0.0015 | 0.3992 |
| Smarcc1 | 0.0028 | 0.4744 |
| Smc1b | 0.0003 | 0.1972 |
| Smcr7l | 0.0016 | 0.3958 |
| Smyd2 | 0.0004 | 0.3136 |
| Smyd3 | 0.0043 | 0.3193 |
| Smyd5 | 0.0003 | 0.3515 |
| Sned1 | 0.0031 | 0.2619 |
| Snhg3 | 0.0003 | 0.4384 |
| Snrpn | 0.0008 | 0.2475 |
| Snx10 | 0.0086 | 0.4277 |
| Snx15 | 0.0001 | 0.3748 |
| Socs2 | 0.0039 | 0.3007 |
| Socs3 | 0.0016 | 0.3005 |
| Sod2 | 0.0001 | 0.2495 |
| Sox2 | 0 | 0.261 |
| Spag6 | 0.0007 | 0.1478 |
| Spic | 0.0007 | 0.0947 |
| Spn | 0.0008 | 0.187 |
| Spry4 | 0.0098 | 0.0161 |
| Srm | 0 | 0.3918 |
| Ssbp4 | 0.0001 | 0.3927 |
| Sssca1 | 0 | 0.4354 |
| St14 | 0.0001 | 0.3087 |
| Stag3 | 0 | 0.3477 |
| Stat4 | 0.0002 | 0.0964 |
| Stat6 | 0.0006 | 0.3394 |
| Stc2 | 0.0001 | 0.1003 |
| Stmn3 | 0.0051 | 0.3846 |
| Stoml1 | 0.0004 | 0.3814 |
| Sugt1 | 0 | 0.4099 |
| Sult4a1 | 0.0038 | 0.3127 |
| Sult6b1 | 0 | 0.0837 |
| Svip | 0.0007 | 0.3927 |
| Svs5 | 0.007 | 0.2199 |
| Syce2 | 0 | 0.4014 |
| Sycp3 | 0.0001 | 0.2161 |
| Syngr1 | 0.0059 0.0019 | 0.3851 0.1117 |
| Syngr4 | 0.003 | 0.3446 |
| Syt4 | 0.0003 | 0.2076 |
| Syt9 | 0.0049 | 0.2907 |
| Tada3l | 0.0003 | 0.3797 |
| Taf1b | 0.0073 | 0.4117 |
| Taf5 | 0.009 | 0.3503 |
| Tanc1 | 0.0035 | 0.4255 |
| Tas1r1 | 0.001 | 0.4374 |
| Tbc1d10c | 0.0001 | 0.2609 |
| Tbc1d24 | 0.0041 | 0.4925 |
| Tbl2 | 0.008 | 0.3808 |
| Tbrg4 | 0.0039 | 0.4337 |
| Tcf15 | 0 | 0.0333 |
| Tcfap2c | 0.0001 | 0.0702 |
| Tcfcp2l1 | 0.0002 | 0.0896 |
| Tdh | 0 | 0.0196 |
| Tdrd12 | 0.0006 | 0.0677 |
| Tet1 | 0.0008 | 0.1186 |
| Tfrc | 0.0004 | 0.195 |
| Tgm1 | 0.009 | 0.2642 |
| Thap1 | 0.0097 | 0.433 |
| Thsd1 | 0 | 0.2936 |
| Tiam2 | 0.0039 | 0.3828 |
| Timm8a2 | 0 | 0.0741 |
| Timm9 | 0.0056 | 0.4209 |
| Tktl2 | 0.0003 | 0.3569 |
| Tle6 | 0.0004 | 0.348 |
| Tlr2 | 0.0001 | 0.4231 |
| Tm7sf3 | 0.006 | 0.4439 |
| Tmc6 | 0.0001 | 0.4198 |
| Tmc7 | 0.0001 | 0.3899 |
| Tmco1 | 0.0062 | 0.2884 |
| Tmco6 | 0.0003 | 0.4248 |
| Tmem125 | 0 | 0.222 |
| Tmem126b | 0.0063 | 0.487 |
| Tmem138 | 0.0008 | 0.4674 |
| Tmem145 | 0.0026 | 0.2777 |
| Tmem177 | 0.0001 | 0.4795 |
| Tmem191c | 0.0 0.0074 | 0.1219 0.3232 |
| Tmem213 | 0.0057 | 0.0644 |
| Tmem38b | 0.0046 | 0.2061 |
| Tmem39a | 0.0001 | 0.4768 |
| Tmem45a | 0 | 0.1574 |
| Tmem45b | 0.0028 | 0.4535 |
| Tmem8 | 0.0006 | 0.2383 |
| Tmem81 | 0.0065 | 0.4152 |
| Tmprss5 | 0.0027 | 0.0763 |
| Tnik | 0.0091 | 0.4477 |
| Tnk1 | 0.0001 | 0.3601 |
| Tnni3 | 0.0001 | 0.2831 |
| Toe1 | 0.0014 | 0.4796 |
| Tpd52 | 0.0009 | 0.3313 |
| Tpd52l1 | 0.0054 | 0.3675 |
| Trak1 | 0.002 | 0.3577 |
| Trap1 | 0.0001 | 0.4582 |
| Trerf1 | 0.0063 | 0.4201 |
| Trim63 | 0.0008 | 0.0646 |
| Triml1 | 0 | 0.0395 |
| Trip4 | 0.0004 | 0.4369 |
| Trmt1 | 0.0001 | 0.3593 |
| Trp73 | 0.0006 | 0.0821 |
| Trps1 | 0 | 0.2226 |
| Trub1 | 0.0001 | 0.4787 |
| Tssc1 | 0.0086 | 0.4908 |
| Tst | 0 | 0.3181 |
| Ttc26 | 0.0051 | 0.2412 |
| Ttc27 | 0 | 0.4421 |
| Ttc38 | 0.0045 | 0.4891 |
| Ttc39b | 0.0021 | 0.391 |
| Ttll13 | 0 | 0.1858 |
| Ttll6 | 0.0059 | 0.2789 |
| Ttn | 0.0003 | 0.03 |
| Tulp3 | 0.0036 | 0.4229 |
| Txlnb | 0.0005 | 0.3006 |
| U2af1 | 0.0045 | 0.4348 |
| Ubash3a | 0 | 0.0875 |
| Ubqln4 | 0.0051 | 0.385 |
| Ubr3 | 0.0058 | 0.45 |
| Ubr7 | 0.0023 | 0.399 |
| Ubtd1 | 0.0001 | 0.4647 |
| Ubxn2a | 0.0024 | 0.3834 |
| Ucma | 0.0024 | 0.0571 |
| Ucp2 | 0.0045 | 0.4317 |
| Unc93a | 0.0006 | 0.073 |
| Upf2 | 0.0097 | 0.4634 |
| Upk2 | 0.0093 | 0.2022 |
| Upk3b | 0.0046 | 0.3569 |
| Upp1 | 0.0027 | 0.032 |
| Urb1 | 0.0009 | 0.3981 |
| Usp48 | 0.0008 | 0.4166 |
| Usp50 | 0.0002 | 0.0352 |
| Usp9x | 0.0082 | 0.4147 |
| Utp14a | 0.0009 | 0.3474 |
| V1rb3 | 0.0007 | 0.3476 |
| V1rb7 | 0.0074 | 0.401 |
| V1re2 | 0.0085 | 0.3746 |
| Vegfc | 0 | 0.2798 |
| Vprbp | 0.0067 | 0.324 |
| Vpreb1 | 0.0006 | 0.4216 |
| Vps13d | 0.0019 | 0.4672 |
| Vps24 | 0.0091 | 0.4676 |
| Vwc2 | 0.0068 | 0.3613 |
| Vwf | 0.0001 | 0.0695 |
| Was | 0.0004 | 0.3498 |
| Wasf2 | 0.0005 | 0.4322 |
| Wbp2nl | 0.0012 | 0.1428 |
| Wdr16 | 0.0081 | 0.2842 |
| Wdr18 | 7.0E-4 0.0013 | 0.4323 0.4674 |
| Wdr20b | 0.0017 | 0.2688 |
| Wdr21 | 0 | 0.3472 |
| Wdr35 | 0.0001 | 0.4598 |
| Wdr47 | 0.0016 | 0.3749 |
| Wdr59 | 0.0001 | 0.488 |
| Wdr74 | 0.0061 | 0.3817 |
| Wdr93 | 0.0088 | 0.4124 |
| Wdsub1 | 0.0064 | 0.3973 |
| Wfdc12 | 0.0002 | 0.3257 |
| Wfdc15a | 0.0004 | 0.1084 |
| Wfikkn2 | 0.0024 | 0.2338 |
| Wif1 | 0.007 | 0.4392 |
| X99384 | 0.005 | 0.3213 |
| Xkr5 | 0.0038 | 0.4819 |
| Xpo4 | 0.0007 | 0.3424 |
| Xrcc5 | 0.0002 | 0.4738 |
| Ydjc | 0.0001 | 0.1633 |
| Zbbx | 0.0015 | 0.4931 |
| Zbtb43 | 0.0045 | 0.4549 |
| Zbtb45 | 0.0001 | 0.4723 |
| Zbtb7a | 0.0014 | 0.3331 |
| Zbtb8a | 0 | 0.261 |
| Zcchc10 | 0.0019 | 0.33 |
| Zcwpw1 | 0 | 0.1828 |
| Zfp101 | 0.0022 | 0.4186 |
| Zfp119 | 0.0006 | 0.4493 |
| Zfp143 | 0.0015 | 0.4433 |
| Zfp229 | 0.0029 | 0.2359 |
| Zfp239 | 0.0012 | 0.4685 |
| Zfp27 | 0.0087 | 0.4745 |
| Zfp273 | 0.0043 | 0.411 |
| Zfp35 | 0.0008 | 0.3573 |
| Zfp418 | 0.0007 | 0.2233 |
| Zfp42 | 0.0004 | 0.0744 |
| Zfp420 | 0.0035 | 0.3752 |
| Zfp451 | 0.0093 | 0.4669 |
| Zfp459 | 0.0062 | 0.019 |
| Zfp518b | 0 | 0.4572 |
| Zfp532 | 0.001 | 0.1747 |
| Zfp654 | 0.0074 | 0.2532 |
| Zfp667 | 0.0008 | 0.2767 |
| Zfp750 | 0.0014 | 0.2669 |
| Zfp759 | 0.0078 | 0.2641 |
| Zfp78 | 0.0003 | 0.1729 |
| Zmat4 | 0.001 | 0.058 |
| Zmym3 | 0.0004 | 0.3013 |
| Zmynd8 | 0.0001 | 0.1489 |
| Znrd1 | 0 | 0.4915 |
| Zp3 | 0.0058 | 0.1682 |
| Zranb3 | 0.0008 | 0.3605 |
| Zswim1 | 0.0002 | 0.2219 |
| Zswim3 | 0.0019 | 0.4093 |
