# Supplementary material for: Retinoic Acid Induces Embryonic Stem Cell Differentiation by Altering Both Encoding RNA and microRNA Expression
Source: PLoS One. 2015 Jul 10;10(7):e0132566. doi: 10.1371/journal.pone.0132566 (PMC4498831; doi:10.1371/journal.pone.0132566)
Supplement: S2 Table — Fold change values were provided in comparison with J1 mESCs treated by DMSO. (DOC) [file pone.0132566.s003.doc]

**Table S2 Significantly up-regulated gene in RA treated J1 mESCs. (Fold Change>2, P-value<0.01)**

Fold change values were provided in comparison with J1 mESCs treated by DMSO.

| Symbol | pvalues | foldchange |
| --- | --- | --- |
| Hoxb3 | 9.0E-4 4.0E-4 | 96.7463 94.0149 |
| Nfatc1 | 7.0E-4 0.0023 | 8.0062 27.0823 |
| Klhl29 | 0.0016 0.0053 | 7.8184 3.6902 |
| Camk2d | 2.0E-4 0.0 | 7.1962 9.4556 |
| Fzd2 | 1.0E-4 0.0 | 6.6799 12.705 |
| Nrip1 | 0.0045 0.0078 | 6.3408 5.2116 |
| E130309F12Rik | 0.0098 0.0057 | 6.1062 5.6139 |
| Epha7 | 0.0096 0.0010 | 5.7274 3.4837 |
| Dsp | 7.0E-4 0.0072 | 5.3415 4.9839 |
| Ppp1r9a | 3.0E-4 2.0E-4 | 4.8332 2.7973 |
| Rcbtb2 | 4.0E-4 7.0E-4 | 3.9752 2.0715 |
| Gpr98 | 0.0095 0.0056 | 3.9602 2.0704 |
| Hip1 | 0.0036 0.0 | 3.9087 5.1263 |
| Zdhhc2 | 0.0013 0.0036 | 3.6957 3.3168 |
| Arsk | 2.0E-4 9.0E-4 | 3.5966 8.8282 |
| H2-Q2 | 2.0E-4 0.0049 | 3.525 2.2736 |
| Neto2 | 0.0059 0.0013 | 3.4956 3.8017 |
| Fgf12 | 0.0077 0.0040 | 3.1579 2.1187 |
| Spsb4 | 5.0E-4 1.0E-4 | 20.0932 25.4917 |
| Usp25 | 6.0E-4 1.0E-4 | 2.9232 2.5051 |
| Tcerg1l | 0.0095 7.0E-4 | 2.5033 2.7473 |
| AU040320 | 2.0E-4 7.0E-4 | 2.4684 2.9614 |
| Limd2 | 0.0011 0.0 | 2.3593 2.795 |
| Fam178a | 0.0026 0.0013 | 2.3586 3.0069 |
| St3gal2 | 0.0060 0.0015 | 2.2815 3.9545 |
| Col4a6 | 2.0E-4 0.0 | 2.1647 71.2402 |
| Uhrf2 | 0.0019 0.0 | 2.0808 2.2876 |
| Grk4 | 2.0E-4 0.0022 | 16.9521 4.2471 |
| Irs1 | 0.0024 0.0 | 16.5691 50.5008 |
| Mex3b | 0.0 0.0033 | 12.123 4.9838 |
| Rfx4 | 1.0E-4 0.0020 | 10.375 15.1605 |
| Ets1 | 0.0037 1.0E-4 | 10.0479 3.5155 |
| Cyp26b1 | 0 | 1131.2478 |
| Hoxc9 | 0 | 978.123 |
| Hoxb6 | 0 | 686.4512 |
| Arg1 | 0.0008 | 544.6851 |
| Hoxd4 | 0.0001 | 480.5408 |
| Cyp26a1 | 0 | 371.1203 |
| Lhx1 | 0.0002 | 267.5457 |
| Tshz1 | 0 | 253.6942 |
| Hoxb5 | 0 | 219.3496 |
| Zic1 | 0.0008 | 182.7573 |
| Dbx1 | 0.0001 | 152.2588 |
| Hoxb9 | 0 | 149.1322 |
| Maf | 0 | 128.6798 |
| Prss23 | 0.0012 | 123.848 |
| Zfp503 | 0 | 111.0671 |
| Plat | 0 | 109.2815 |
| Hoxb2 | 0 | 95.8088 |
| Stmn4 | 0.0005 | 90.4674 |
| Ednrb | 0.0004 | 89.2937 |
| Nedd9 | 0.001 | 86.9866 |
| Acta2 | 0.0001 | 83.4895 |
| Cxcl1 | 0.001 | 82.9613 |
| Foxc2 | 0.0012 | 81.9105 |
| Col1a2 | 0 | 81.2374 |
| Cyp1b1 | 0.0001 | 75.6002 |
| Igfbp5 | 0 | 68.1049 |
| Ecm1 | 0 | 66.0822 |
| Sema3c | 0.0002 | 63.9598 |
| Speer7-ps1 | 0.0001 | 61.4535 |
| Hoxa5 | 0 | 60.2393 |
| 5730446D14Rik | 0.0036 | 59.3417 |
| Lpar3 | 0 | 58.3029 |
| Acta1 | 0.0001 | 56.999 |
| 1810011O10Rik | 0 | 52.7715 |
| Gas1 | 0 | 49.9846 |
| Hoxb1 | 0 | 47.6174 |
| Fbn2 | 0 | 44.5621 |
| Plau | 0.0006 | 44.0624 |
| Foxd1 | 0 | 43.3984 |
| Tcstv1 | 0 | 42.5987 |
| Tcstv3 | 0 | 41.9 |
| Phlda1 | 0 | 41.7521 |
| Pax3 | 0.0001 | 41.723 |
| Sox9 | 0.0011 | 41.205 |
| Cxcl3 | 0.0007 | 41.196 |
| Ccl3 | 0 | 40.6647 |
| Lgals6 | 0 | 40.0278 |
| Aldh1a7 | 0.0005 | 39.5831 |
| Tshz2 | 0.0001 | 38.4959 |
| 1700031F05Rik | 0.0007 | 38.4202 |
| mCG_21548 | 0 | 37.8189 |
| Ankrd1 | 0.001 | 37.7157 |
| Arg2 | 0.0002 | 37.2461 |
| Tgfb3 | 0 | 36.7093 |
| Edn1 | 0 | 36.28 |
| Tgfb2 | 0.0042 | 36.2789 |
| Zfp608 | 0.0003 | 36.2089 |
| EG625530 | 0.0008 | 33.5781 |
| Csf1 | 0 | 32.9839 |
| Tagln | 0 | 32.4514 |
| Zscan4c | 0.0004 | 32.0954 |
| Hoxb7 | 0.0001 | 32.0396 |
| Hoxa2 | 0 | 31.5946 |
| 2810055G20Rik | 0 | 31.5943 |
| Grifin | 0.0073 | 30.7828 |
| Pax6 | 0.0017 | 29.6679 |
| Ankrd22 | 0.0037 | 29.5337 |
| Irx5 | 0 | 29.5283 |
| Zadh2 | 0.0001 | 29.0343 |
| Scara3 | 0 | 29.0238 |
| Ppbp | 0.0001 | 28.5924 |
| Cnrip1 | 0.0001 | 28.3231 |
| Thsd4 | 0.001 | 27.4951 |
| Ednra | 0.0006 | 27.1694 |
| Ebf3 | 0.0001 | 26.9786 |
| Cd36 | 0.0032 | 26.598 |
| Dchs1 | 0 | 26.566 |
| OTTMUSG00000008561 | 0.0009 | 26.5133 |
| A530040E14Rik | 0 | 26.0843 |
| Svep1 | 0 | 25.7449 |
| Hmx3 | 0.0001 | 24.6334 |
| Vat1l | 0.0001 | 24.235 |
| Nrp1 | 0.0031 | 24.0817 |
| Samd14 | 0 | 23.9631 |
| Sdpr | 0 | 22.8507 |
| Irf2 | 0.0001 | 22.6584 |
| Peg10 | 0.0023 | 22.6405 |
| Osmr | 0.0018 | 22.3252 |
| Spz1 | 0.0007 | 22.2079 |
| Krt18 | 0.0001 | 22.0572 |
| Ppargc1a | 0.0017 | 21.546 |
| Pitx1 | 0.0041 | 21.4824 |
| Fras1 | 0.0003 | 21.2769 |
| Crb2 | 0 | 21.1392 |
| Csn3 | 0 | 21.1294 |
| Lin7a | 0.0011 | 21.1254 |
| Ccnd2 | 0 | 21.0496 |
| Irx1 | 0 | 21.0177 |
| Olfml3 | 0 | 21.0158 |
| Shd | 0 | 20.8742 |
| Fndc5 | 0 | 20.5364 |
| Adamts1 | 0 | 20.5213 |
| Cacna1g | 0.0025 | 20.4231 |
| Osr2 | 0.0032 | 20.3252 |
| Slc7a10 | 0.0006 | 20.11 |
| 9030224M15Rik | 0.0003 | 20.1078 |
| Igg2a | 0.0009 | 20.0627 |
| Tmem176a | 0.0021 | 19.9839 |
| Gpr177 | 0.0001 | 19.9637 |
| Hoxd9 | 0.0017 | 19.8852 |
| Antxr2 | 0.0003 | 19.6085 |
| Olfr713 | 0 | 19.5121 |
| Gm428 | 0.0002 | 19.5047 |
| Lgals4 | 0 | 19.2839 |
| Cd248 | 0 | 18.4333 |
| Plxna2 | 0.0006 | 18.3915 |
| Asb4 | 0.0003 | 18.048 |
| Synpo | 0.0027 | 17.9219 |
| Dcx | 0.0016 | 17.6875 |
| Col3a1 | 0 | 17.6837 |
| Sncaip | 0.0016 | 17.5146 |
| Cdc42ep1 | 0 | 17.4335 |
| Hoxd1 | 0 | 16.6612 |
| Lbp | 0.0022 | 16.5347 |
| Agtr1a | 0.0006 | 16.5205 |
| Zfp521 | 0.0027 | 16.4582 |
| Dcn | 0.0017 | 16.4391 |
| Tmem56 | 0.0005 | 16.3398 |
| Naaladl2 | 0.0001 | 16.1611 |
| Cldn2 | 0.0009 | 15.9211 |
| Rab36 | 0.0025 | 15.6227 |
| Prkg1 | 0.0009 | 15.5993 |
| Id3 | 0.0001 | 15.5413 |
| Gria3 | 0.0001 | 15.5176 |
| Cnn1 | 0.0004 | 15.4898 |
| Areg | 0.0077 | 15.4188 |
| Tspan18 | 0 | 15.2314 |
| Dtx4 | 0.0001 | 15.2256 |
| Pdlim3 | 0 | 15.1274 |
| 1700011H14Rik | 0.0008 | 14.9855 |
| Lhx2 | 0 | 14.9783 |
| Pgm5 | 0 | 14.7119 |
| Fhl2 | 0 | 14.6138 |
| Thbs4 | 0.0013 | 14.6115 |
| Rtn4r | 0.0068 | 14.5911 |
| Nsg1 | 0 | 14.5591 |
| Sytl2 | 0.0003 | 14.4555 |
| Rec8 | 0 | 14.4093 |
| Atf3 | 0.0012 | 14.3926 |
| Cpm | 0 | 14.3623 |
| Dusp8 | 0.0016 | 14.3121 |
| 4921506M07Rik | 0.0006 | 14.2927 |
| Ppp2r2b | 0.0017 | 14.2703 |
| 0610040J01Rik | 0 | 14.1939 |
| Parva | 0.0001 | 14.1121 |
| Speg | 0 | 14.0952 |
| Islr | 0 | 14.0477 |
| Fads3 | 0.0009 | 13.9256 |
| Efna5 | 0.0001 | 13.7047 |
| Twist2 | 0.0022 | 13.7027 |
| Prss12 | 0.0008 | 13.6824 |
| Id2 | 0 | 13.6449 |
| Olfr881 | 0 | 13.4855 |
| Isx | 0.0001 | 13.4235 |
| Cpb2 | 0 | 13.217 |
| Bnc1 | 0.0098 | 13.1866 |
| Kynu | 0.0081 | 13.1487 |
| Plagl1 | 0.0002 | 13.1277 |
| Cdh11 | 0.0064 | 13.0321 |
| Fut4 | 0.0002 | 13.0313 |
| Rasgrp3 | 0 | 12.9618 |
| Serpinf1 | 0.0005 | 12.948 |
| Fam149a | 0 | 12.923 |
| Tpo | 0.0005 | 12.5219 |
| Grin3b | 0.0002 | 12.5176 |
| Cplx2 | 0 | 12.4445 |
| Slpi | 0.0003 | 12.397 |
| Cbx4 | 0.0003 | 12.3493 |
| Mest | 0.0005 | 12.349 |
| Frk | 0.0009 | 11.9816 |
| Hs6st2 | 0.0005 | 11.9743 |
| Gal3st4 | 0.0024 | 11.9292 |
| Pde10a | 0 | 11.8583 |
| Msx1 | 0.0011 | 11.7254 |
| Slc25a24 | 0 | 11.6704 |
| Vit | 0.001 | 11.5865 |
| Peg3 | 0.0012 | 11.53 |
| Prkd1 | 0.0024 | 11.4168 |
| OTTMUSG00000002043 | 0.0009 | 11.3607 |
| Tmprss2 | 0.0001 | 11.2501 |
| Trim9 | 0.0002 | 11.2099 |
| Sox6 | 0.0014 | 11.2092 |
| Lrrc17 | 0.0082 | 11.1646 |
| Cd24a | 0 | 11.163 |
| St6galnac5 | 0.0057 | 11.1115 |
| Mafb | 0.0007 | 10.9958 |
| 1700009N14Rik | 0.0004 | 10.8803 |
| Onecut3 | 0.0043 | 10.7719 |
| Lgals1 | 0 | 10.7575 |
| St3gal1 | 0 | 10.7403 |
| Mefv | 0.0018 | 10.7331 |
| Prtg | 0.0001 | 10.6913 |
| Speer4d | 0.0001 | 10.6305 |
| Olfml2b | 0.0008 | 10.6067 |
| Kcne1l | 0.0005 | 10.6058 |
| Glis3 | 0.0041 | 10.5983 |
| Slc2a10 | 0.007 | 10.537 |
| Parp8 | 0 | 10.5298 |
| Parvg | 0.0015 | 10.4661 |
| Kdr | 0.0003 | 10.4469 |
| Ajap1 | 0.0003 | 10.3412 |
| Gabra1 | 0.0004 | 10.1107 |
| Ptx3 | 0.0003 | 10.0524 |
| Nkx2-5 | 0 | 9.9198 |
| Itga11 | 0.0001 | 9.7906 |
| Hmga2 | 0.0007 | 9.7228 |
| Kcna6 | 0.0058 | 9.72 |
| EG266459 | 0 | 9.6842 |
| Twist1 | 0.0001 | 9.6198 |
| S1pr1 | 0.0011 | 9.5732 |
| Inpp4b | 0.0037 | 9.5331 |
| Gm1008 | 0.0051 | 9.5217 |
| Maob | 0.0017 | 9.5079 |
| Prkch | 0.004 | 9.4808 |
| Lama4 | 0.0001 | 9.438 |
| 1700010D01Rik | 0.0046 | 9.4294 |
| V1re6 | 0.0012 | 9.4276 |
| Adcy2 | 0.0048 | 9.3712 |
| Tas1r3 | 0.0043 | 9.359 |
| Cyr61 | 0.0024 | 9.3556 |
| Fam115a | 0 | 9.3479 |
| Rhbdl3 | 0.0004 | 9.3021 |
| Scd3 | 0.0009 | 9.2444 |
| Dll4 | 0.0005 | 9.1764 |
| Zbtb7c | 0.0002 | 9.1138 |
| Msrb3 | 0.0023 | 9.0942 |
| Rbm24 | 0 | 9.0879 |
| Nnat | 0.0003 | 9.0315 |
| Gpc3 | 0.0019 | 9.0197 |
| Fbxo32 | 0.0002 | 8.9986 |
| Pif1 | 0.0002 | 8.9811 |
| Hoxa7 | 0.0002 | 8.9793 |
| Pcdh17 | 0.0074 | 8.9574 |
| Cacna1s | 0.0002 | 8.9508 |
| Gprc5c | 0 | 8.9432 |
| Rarres2 | 0.0003 | 8.8639 |
| Adamts15 | 0 | 8.8238 |
| Efnb1 | 0 | 8.7817 |
| Unc5c | 0.0001 | 8.7624 |
| Cmtm3 | 0 | 8.7256 |
| Clstn2 | 0.004 | 8.6912 |
| Efemp2 | 0 | 8.6262 |
| Mfsd6l | 0.0066 | 8.6134 |
| Bmp7 | 0 | 8.6055 |
| Iqcf1 | 0 | 8.5513 |
| Rgs10 | 0 | 8.5349 |
| Fbxl7 | 0 | 8.5246 |
| Cmbl | 0.0002 | 8.5138 |
| Leprel1 | 0 | 8.4363 |
| Rhox6 | 0.0007 | 8.3668 |
| Crabp1 | 0 | 8.3584 |
| Tmigd1 | 0.0011 | 8.325 |
| Ripply3 | 0.0014 | 8.3101 |
| Tmem171 | 0.0028 | 8.3081 |
| Pear1 | 0.0031 | 8.3051 |
| Ramp1 | 0.0009 | 8.2564 |
| Fam162b | 0.0001 | 8.2446 |
| Tec | 0 | 8.2379 |
| 100039742 | 0.0002 | 8.1761 |
| Echdc3 | 0 | 8.161 |
| Hmgn3 | 0 | 8.1255 |
| Lbh | 0.0001 | 8.0955 |
| AK220484 | 0 | 8.0269 |
| Rspo2 | 0.0009 | 8.0029 |
| Mpped2 | 0.0002 | 7.9961 |
| Scx | 0 | 7.9261 |
| Ifng | 0.0053 | 7.9082 |
| Fgfr2 | 0.0023 | 7.88 |
| Nts | 0 | 7.8522 |
| Cbx8 | 0.0015 | 7.8167 |
| Plcl2 | 0.0003 | 7.8027 |
| Pon1 | 0.0001 | 7.7996 |
| F11r | 0.0025 | 7.7908 |
| Snx33 | 0.0001 | 7.7834 |
| Igdcc3 | 0.0005 | 7.7612 |
| Sp5 | 0.0002 | 7.7408 |
| Faim3 | 0.004 | 7.7089 |
| Serpinh1 | 0.0002 | 7.6862 |
| Pcdhb21 | 0.0083 | 7.6712 |
| Adamts19 | 0.0009 | 7.6633 |
| Adcy4 | 0.0089 | 7.5894 |
| Cpne2 | 0.0009 | 7.5751 |
| 1700030F18Rik | 0.0014 | 7.5423 |
| EG382639 | 0.0029 | 7.5177 |
| Cybrd1 | 0.0019 | 7.452 |
| Slc22a15 | 0.0015 | 7.4174 |
| Wnt4 | 0.0003 | 7.3635 |
| Ascl1 | 0.0069 | 7.3512 |
| Tshz3 | 0 | 7.3396 |
| Chrna4 | 0.0002 | 7.3291 |
| Mrc2 | 0.0013 | 7.327 |
| Krt20 | 0.0002 | 7.3095 |
| LOC675747 | 0.0002 | 7.304 |
| Mogat2 | 0 | 7.2625 |
| Rhox9 | 0.0002 | 7.2399 |
| Dnaja4 | 0.0008 | 7.2338 |
| Fzd1 | 0 | 7.1471 |
| Pxt1 | 0.0001 | 7.1198 |
| Ism1 | 0 | 7.047 |
| Lipg | 0.0022 | 6.9975 |
| Pcdhb22 | 0.0087 | 6.9557 |
| Nkg7 | 0.0037 | 6.9475 |
| Mfhas1 | 0.0013 | 6.926 |
| Hdc | 0.0005 | 6.9127 |
| Tspan6 | 0.0001 | 6.9105 |
| Fgfbp1 | 0.0046 | 6.9073 |
| Irx2 | 0.0026 | 6.9064 |
| Lmo1 | 0.0003 | 6.902 |
| Adamts16 | 0.0031 | 6.8764 |
| Fam102b | 0 | 6.8539 |
| Wisp1 | 0.0025 | 6.8495 |
| Frzb | 0.0008 | 6.8149 |
| Ntn1 | 0.0042 | 6.812 |
| Cald1 | 0.0002 | 6.8109 |
| Wdr72 | 0.0008 | 6.7834 |
| Syde1 | 0.0002 | 6.7786 |
| St8sia1 | 0.0003 | 6.7499 |
| AF067063 | 0.0004 | 6.6841 |
| 2510049J12Rik | 0.0007 | 6.6436 |
| Gli3 | 0 | 6.6261 |
| Lypd6b | 0.0019 | 6.6065 |
| Gdpd5 | 0.0008 | 6.5954 |
| Gpx3 | 0 | 6.5628 |
| Cd83 | 0.0002 | 6.5397 |
| Syn2 | 0.0021 | 6.5391 |
| Aif1l | 0 | 6.5372 |
| Fam151a | 0.0062 | 6.5305 |
| Alcam | 0.0003 | 6.5038 |
| Arsb | 0.0004 | 6.485 |
| Zfp202 | 0 | 6.4578 |
| EG210155 | 0.0035 | 6.4255 |
| Hspa12b | 0 | 6.3948 |
| Wnt9b | 0.0098 | 6.3891 |
| Opn3 | 0.0003 | 6.3734 |
| Fbn1 | 0.0014 | 6.3723 |
| Slit3 | 0 | 6.3687 |
| Gpr124 | 0.0006 | 6.3273 |
| Ptplad2 | 0.0002 | 6.3198 |
| Snai1 | 0 | 6.3078 |
| Otor | 0.0021 | 6.2845 |
| Tnnt3 | 0 | 6.2664 |
| Filip1l | 0.0007 | 6.2459 |
| Cdon | 0 | 6.2127 |
| Eraf | 0.0053 | 6.2103 |
| Heg1 | 0.0017 | 6.1999 |
| Nudt18 | 0.0041 | 6.1982 |
| Smpd3 | 0.0034 | 6.1868 |
| Samd5 | 0.0047 | 6.1543 |
| Tie1 | 0.0009 | 6.0885 |
| Timp2 | 0 | 6.0852 |
| Pdgfra | 0.0003 | 6.0824 |
| AI314831 | 0.0007 | 6.0476 |
| Fat3 | 0 | 6.0361 |
| Farp1 | 0 | 6.0255 |
| 3830417A13Rik | 0.0023 | 5.9374 |
| 2600010E01Rik | 0.0001 | 5.9177 |
| Tbx15 | 0.0009 | 5.9147 |
| Kcna5 | 0.0006 | 5.9047 |
| Adcy6 | 0.0001 | 5.8914 |
| Ndn | 0.0016 | 5.8913 |
| Slc24a2 | 0.0068 | 5.8628 |
| 2610109H07Rik | 0.0054 | 5.8486 |
| Spaca1 | 0 | 5.8182 |
| Clic6 | 0 | 5.8107 |
| Cyp4f15 | 0.0015 | 5.7848 |
| Metrn | 0.0016 | 5.7773 |
| Hist1h4i | 0 | 5.7345 |
| 2700045P11Rik | 0.0001 | 5.6814 |
| Mcoln3 | 0.0002 | 5.6228 |
| Fam123a | 0 | 5.6182 |
| Igtp | 0 | 5.6048 |
| Creg1 | 0.0091 | 5.5639 |
| Tmem173 | 0.0001 | 5.5482 |
| Larp6 | 0.0003 | 5.5423 |
| Crym | 0 | 5.535 |
| Plekho1 | 0.0002 | 5.5333 |
| Col6a2 | 0.0033 | 5.5215 |
| Cnn2 | 0 | 5.4987 |
| Batf3 | 0.0008 | 5.4921 |
| Tpm2 | 0.0018 | 5.481 |
| Hist3h2a | 0 | 5.4217 |
| Cda | 0.0001 | 5.416 |
| Hist1h1c | 0 | 5.4124 |
| Col4a2 | 0.001 | 5.3953 |
| Fam101b | 0.0033 | 5.3642 |
| Ak3 | 0 | 5.3503 |
| Cdkn1c | 0.0075 | 5.3457 |
| Col24a1 | 0.0033 | 5.3257 |
| Gata6 | 0 | 5.3155 |
| Krtdap | 0.0023 | 5.3067 |
| Krt19 | 0 | 5.2906 |
| Shisa4 | 0.0001 | 5.2904 |
| Ifi27l2a | 0.0001 | 5.2819 |
| Whsc1 | 0.0005 | 5.2793 |
| Dmrta2 | 0.0004 | 5.277 |
| Cdgap | 0.0012 | 5.1861 |
| Gjb6 | 0.0072 | 5.1775 |
| Arap3 | 0.0001 | 5.1724 |
| Psd3 | 0.0007 | 5.1698 |
| Cmtm8 | 0 | 5.157 |
| Hmx1 | 0.0003 | 5.1495 |
| Ikzf2 | 0.0009 | 5.1459 |
| Ltbr | 0.0032 | 5.1414 |
| Cd74 | 0.0001 | 5.1282 |
| Nuak1 | 0.0022 | 5.1148 |
| Gm447 | 0.0044 | 5.1001 |
| Tnf | 0.0028 | 5.0936 |
| Dlx2 | 0.002 | 5.0657 |
| Flt1 | 0 | 5.053 |
| Tgfbr2 | 0.0009 | 5.0421 |
| Gfra1 | 0.0002 | 5.0215 |
| Afp | 0.0018 | 5.0186 |
| Nrk | 0.0099 | 4.9984 |
| Ttc28 | 0.0014 | 4.9976 |
| 40969 | 0 | 4.9719 |
| Gjb2 | 0.0007 | 4.934 |
| Hs3st3a1 | 0.0043 | 4.9332 |
| Ccl2 | 0.0034 | 4.929 |
| Cap2 | 0.0012 | 4.9219 |
| B630019K06Rik | 0.0004 | 4.8914 |
| Plcb1 | 0.0013 | 4.8859 |
| Baiap2l1 | 0 | 4.8827 |
| AI646023 | 0.0024 | 4.8713 |
| Gyg | 0 | 4.8698 |
| Tmem178 | 0 | 4.8656 |
| Nbl1 | 0 | 4.8616 |
| Cldn10 | 0.0033 | 4.8552 |
| Sall2 | 0 | 4.8529 |
| Cldn11 | 0.0012 | 4.8387 |
| Emilin2 | 0.0011 | 4.8191 |
| Slc35f1 | 0.0001 | 4.8042 |
| E130112L23Rik | 0.0001 | 4.7969 |
| Ms4a4d | 0.0001 | 4.7771 |
| Setbp1 | 0.0004 | 4.7545 |
| Speer5-ps1 | 0.0012 | 4.7505 |
| 5033430I15Rik | 0 | 4.7366 |
| Lpp | 0 | 4.7219 |
| Gata2 | 0.0009 | 4.7185 |
| B2m | 0.0022 | 4.6922 |
| 1110032E23Rik | 0.0002 | 4.6365 |
| Pdk3 | 0.0001 | 4.6348 |
| 1700052K11Rik | 0.0002 | 4.6317 |
| Tnfaip2 | 0.0017 | 4.6291 |
| Tmem59l | 0.0002 | 4.6266 |
| Cx3cl1 | 0.0003 | 4.6118 |
| Glt25d2 | 0.0073 | 4.6112 |
| Pcdh7 | 0.0027 | 4.6028 |
| 4930415F15Rik | 0.0001 | 4.5762 |
|  | 0.007 | 4.5762 |
| Dpysl2 | 0.0007 | 4.5556 |
| Tbxa2r | 0.0005 | 4.5523 |
| 41156 | 0.0002 | 4.5512 |
| Vasn | 0.0006 | 4.541 |
| Lrrk1 | 0.0003 | 4.5363 |
| EG628779 | 0.0008 | 4.456 |
| Slc6a17 | 0.0049 | 4.4488 |
| Car3 | 0.0015 | 4.4323 |
| Fezf2 | 0.01 | 4.4256 |
| Mfsd7c | 0.0023 | 4.4125 |
| Gulp1 | 0.0001 | 4.3959 |
| Capn6 | 0.0074 | 4.3905 |
| Bves | 0.0007 | 4.3798 |
| Metrnl | 0.0008 | 4.3612 |
| Cyba | 0.0025 | 4.3524 |
| 1700016G05Rik | 0.0051 | 4.3305 |
| Olfr1145 | 0.0068 | 4.3104 |
| Mbp | 0.0005 | 4.2977 |
| Prkcq | 0.0006 | 4.2974 |
| 6330403K07Rik | 0.0001 | 4.283 |
| Gbp2 | 0.0019 | 4.2818 |
| Pth1r | 0.0046 | 4.2558 |
| Irg1 | 0.0006 | 4.2502 |
| Abtb2 | 0.0003 | 4.244 |
| Rasl10b | 0.0001 | 4.223 |
| Aldh1a1 | 0.0066 | 4.2212 |
| Rrad | 0.0006 | 4.2033 |
| Glipr1 | 0.0035 | 4.1854 |
| Thbd | 0.0023 | 4.1847 |
| Socs5 | 0.0027 | 4.1838 |
| Gpx8 | 0.0001 | 4.1768 |
| Ehd2 | 0.0004 | 4.1703 |
| Slc22a4 | 0.0022 | 4.1595 |
| Tmtc2 | 0.0008 | 4.1571 |
| LOC100046622 | 0.0002 | 4.1567 |
| Dkk1 | 0.0087 | 4.1504 |
| Tmem162 | 0.0058 | 4.1335 |
| Asphd2 | 0 | 4.1301 |
| Ccdc88b | 0.0019 | 4.1278 |
| Tinagl1 | 0.0003 | 4.1211 |
| Letmd1 | 0.0011 | 4.1107 |
| Hivep2 | 0.0014 | 4.1015 |
| Mapkapk2 | 0 | 4.0903 |
| Cacnb2 | 0.0038 | 4.0901 |
| Stx11 | 0.0061 | 4.0827 |
| Myl1 | 0.0003 | 4.0825 |
| Cstad | 0.0076 | 4.079 |
| Hoxd13 | 0.0014 | 4.0732 |
| Tnfaip8l2 | 0.0003 | 4.0721 |
| 1300007F04Rik | 0.0003 | 4.0694 |
| Rasl12 | 0 | 4.0613 |
| 9130213B05Rik | 0 | 4.0585 |
| Emilin1 | 0.0006 | 4.0568 |
| Mst1r | 0 | 4.0566 |
| Galnt14 | 0.0001 | 4.0525 |
| Antxr1 | 0.0001 | 4.0406 |
| Slfn2 | 0.0043 | 4.0404 |
| Gdap1 | 0.0039 | 4.0373 |
| Olfr774 | 0.0006 | 4.0369 |
| Cxcr4 | 0.0034 | 4.036 |
| Ugcgl2 | 0.004 | 4.0349 |
| Edil3 | 0.0029 | 4.0348 |
| Ctdspl | 0 | 4.0094 |
| 4833427G06Rik | 0.0023 | 4.002 |
| Gatm | 0.0002 | 3.9974 |
| Jam3 | 0.0008 | 3.9877 |
| Serp2 | 0 | 3.973 |
| C030003D03Rik | 0.0063 | 3.967 |
| Endod1 | 0 | 3.9547 |
| Copz2 | 0.0001 | 3.9481 |
| Ppm1e | 0.0032 | 3.9447 |
| Sorcs2 | 0 | 3.9297 |
| Csdc2 | 0.0008 | 3.9147 |
| Zfpm2 | 0.0001 | 3.9132 |
| Cpd | 0 | 3.9131 |
| Fam181b | 0 | 3.8996 |
| Sct | 0.0002 | 3.8956 |
| 4931406H21Rik | 0.0099 | 3.8942 |
| Lta | 0 | 3.8938 |
| Ica1l | 0.0048 | 3.8877 |
| Fign | 0.0002 | 3.8658 |
| 6330409D20Rik | 0.0031 | 3.8397 |
| Rgl1 | 0.0006 | 3.8392 |
| Ror1 | 0.0017 | 3.837 |
| Ubash3b | 0.0006 | 3.8367 |
| Grik5 | 0.0019 | 3.835 |
| Pcdh20 | 0.0081 | 3.8275 |
| Sst | 0.0004 | 3.8246 |
| Timp3 | 0.0001 | 3.8229 |
| Sesn3 | 0.0011 | 3.8211 |
| Rnd3 | 0.0004 | 3.8147 |
| Whrn | 0 | 3.808 |
| Sh3bgrl | 0 | 3.7971 |
| Klf14 | 0.0048 | 3.7856 |
| Bmi1 | 0 | 3.7848 |
| Tesk1 | 0.0001 | 3.7838 |
| Mxra8 | 0.0024 | 3.771 |
| Tgm2 | 0.0001 | 3.764 |
| 1700008I05Rik | 0.0008 | 3.7609 |
| Lrrn1 | 0.003 | 3.7604 |
| Dock11 | 0.0001 | 3.7561 |
| Lonrf3 | 0 | 3.7534 |
| Refbp2 | 0 | 3.7412 |
| Maged1 | 0 | 3.7259 |
| Slc40a1 | 0.0003 | 3.725 |
| AI132487 | 0 | 3.7191 |
| Gng10 | 0.0002 | 3.7045 |
| Fzd7 | 0.0002 | 3.6965 |
| Ano10 | 0.0002 | 3.6931 |
| Fam20c | 0.0002 | 3.6788 |
| Reep3 | 0.0001 | 3.6739 |
| Sdc3 | 0.0002 | 3.6728 |
| F9 | 0.0038 | 3.6715 |
| Krt7 | 0.0019 | 3.6679 |
| Mmp15 | 0.0009 | 3.6575 |
| 4631416L12Rik | 0.0023 | 3.6513 |
| Rhou | 0 | 3.6509 |
| Gm88 | 0.0095 | 3.6469 |
| Senp7 | 0.0025 | 3.6469 |
| Rab6b | 0.0001 | 3.6341 |
| Osbpl6 | 0.0007 | 3.6278 |
| Gpr162 | 0.0002 | 3.595 |
| Fbln7 | 0.0002 | 3.593 |
| Setd7 | 0.0002 | 3.5888 |
| Ptprz1 | 0 | 3.5844 |
| Mapk12 | 0.0005 | 3.5801 |
| Cd5l | 0.0095 | 3.5773 |
| Cpxm2 | 0.0008 | 3.5747 |
| Smo | 0.0001 | 3.5701 |
| Pgcp | 0.0002 | 3.5663 |
| Irf8 | 0.0003 | 3.5632 |
| Ncf4 | 0.0044 | 3.561 |
| Nrp2 | 0.0011 | 3.5553 |
| Six1 | 0.0025 | 3.5539 |
| Mt2 | 0.0029 | 3.5458 |
| Tug1 | 0.006 | 3.5419 |
| Agbl4 | 0.0058 | 3.5335 |
| Zfp318 | 0.0047 | 3.5309 |
| Fam55d | 0.0026 | 3.5288 |
| Padi1 | 0.0016 | 3.5274 |
| Alx3 | 0.0002 | 3.5219 |
| Zfp768 | 0.0008 | 3.5164 |
| Gbp1 | 0.0013 | 3.5031 |
| P2ry5 | 0 | 3.5005 |
| Reep5 | 0.0022 | 3.4912 |
| Sox7 | 0.0009 | 3.4834 |
| Lbx2 | 0.0007 | 3.4781 |
| Zeb2 | 0 | 3.469 |
| H2-T23 | 0 | 3.4556 |
| 9130008F23Rik | 0.0004 | 3.4545 |
| Slc43a3 | 0 | 3.4542 |
| Nexn | 0 | 3.4538 |
| Accn1 | 0.0001 | 3.4536 |
| Ttyh3 | 0.0001 | 3.4099 |
| 8430427H17Rik | 0.0006 | 3.4016 |
| Gdpd1 | 0.0001 | 3.3915 |
| Cugbp2 | 0.0002 | 3.3894 |
| Med12l | 0.0028 | 3.3789 |
| 4930426D05Rik | 0.0035 | 3.3774 |
| Fam13a | 0.0055 | 3.3751 |
| Emp3 | 0 | 3.3674 |
| Neurog2 | 0.0079 | 3.3654 |
| 4930506M07Rik | 0.0001 | 3.3606 |
| Fam120b | 0.0006 | 3.3576 |
| Cuedc1 | 0.0013 | 3.3535 |
| Lor | 0.0001 | 3.345 |
| H2-K1 | 0 | 3.3448 |
| Gsg1l | 0.0031 | 3.3363 |
| Pcbp4 | 0.0038 | 3.3356 |
| St3gal5 | 0.0005 | 3.3277 |
| Pax1 | 0.0015 | 3.3261 |
| Atp6v1c2 | 0.0023 | 3.3186 |
| Ppp4r4 | 0.0016 | 3.3159 |
| Gpr37l1 | 0.0069 | 3.2962 |
| Olfr738 | 0.0004 | 3.2943 |
| Amotl2 | 0.0005 | 3.2909 |
| Amigo3 | 0.0033 | 3.2803 |
| Ppm1l | 0 | 3.2766 |
| Pon3 | 0.0011 | 3.2764 |
| Yaf2 | 0.0037 | 3.2674 |
| Phlda3 | 0.0002 | 3.2649 |
| Tmem140 | 0.0006 | 3.2632 |
| Hist1h1e | 0.0025 | 3.2629 |
| Irgm1 | 0 | 3.2606 |
| Nr4a2 | 0.0035 | 3.2592 |
| Fbxl16 | 0.003 | 3.2589 |
| Adcyap1r1 | 0.0025 | 3.2584 |
| Rab32 | 0.0002 | 3.2511 |
| Pctp | 0.0006 | 3.2488 |
| Gdf11 | 0.0022 | 3.2426 |
| 1190003M12Rik | 0.0018 | 3.2304 |
| Pygl | 0.0003 | 3.229 |
| Mx2 | 0.0035 | 3.2279 |
| Srgap3 | 0 | 3.2265 |
| Ropn1l | 0 | 3.2098 |
| Rgs16 | 0.0007 | 3.2085 |
| Smpd1 | 0.0025 | 3.2056 |
| OTTMUSG00000015204 | 0.0095 | 3.2033 |
| Tmcc3 | 0.0005 | 3.1999 |
| Pcdh8 | 0.0018 | 3.1967 |
| Arhgef5 | 0.0027 | 3.1896 |
| Stox2 | 0 | 3.1892 |
| Mfsd2 | 0.0001 | 3.1889 |
| Slc4a3 | 0.0018 | 3.1856 |
| Chi3l3 | 0.0004 | 3.1785 |
| Crybg3 | 0.0008 | 3.1741 |
| Lrp3 | 0 | 3.173 |
| Klhdc8b | 0.0025 | 3.1528 |
| Oaf | 0.0012 | 3.1457 |
| Tmem18 | 0 | 3.1403 |
| Rin1 | 0.0088 | 3.1398 |
| AA986860 | 0.0057 | 3.1389 |
| Tmed3 | 0 | 3.1374 |
| Cpne8 | 0.0014 | 3.1364 |
| 8030474K03Rik | 0 | 3.1342 |
| Bag2 | 0.0039 | 3.1292 |
| B4galt4 | 0 | 3.1229 |
| Slc9a6 | 0.0061 | 3.1224 |
| Rcsd1 | 0.0021 | 3.12 |
| Chst11 | 0.0021 | 3.1155 |
| Sh3rf1 | 0.0009 | 3.1025 |
| Zmynd8 | 0.0032 | 3.0991 |
| Rnf217 | 0.001 | 3.0936 |
| Coch | 0.0072 | 3.0862 |
| Col5a1 | 0 | 3.0837 |
| Rnf103 | 0.0046 | 3.0779 |
| Rgs8 | 0.0005 | 3.0776 |
| LOC100048439 | 0 | 3.0734 |
| Kif26b | 0.008 | 3.0637 |
| Eif2c4 | 0.0017 | 3.0563 |
| Galntl1 | 0 | 3.0462 |
| Syt13 | 0.0002 | 3.0454 |
| P4ha2 | 0.0004 | 3.0454 |
| Efha1 | 0.0003 | 3.0443 |
| Tmem88 | 0.0009 | 3.0201 |
| Pcsk5 | 0.004 | 3.0158 |
| Rbms1 | 0.0091 | 3.0134 |
| Tnfrsf1a | 0 | 3.0044 |
| Ccdc153 | 0.0025 | 3.0021 |
| Bmp2k | 0.0001 | 3.002 |
| Tlx1 | 0.0013 | 2.999 |
| Efs | 0 | 2.9909 |
| Pcdh19 | 0 | 2.9872 |
| Limd1 | 0.0001 | 2.9823 |
| Wnt11 | 0 | 2.9772 |
| Clip3 | 0.0002 | 2.972 |
| Ppp1r3d | 0.0033 | 2.9707 |
| Zg16 | 0.007 | 2.9647 |
| Lgals3bp | 0.0001 | 2.9625 |
| Nrxn1 | 0.0081 | 2.9535 |
| Itga8 | 0.0074 | 2.9511 |
| Atrnl1 | 0.001 | 2.9506 |
| Rasl11b | 0.0006 | 2.9493 |
| Gpr176 | 0.0045 | 2.9443 |
| Rtn4rl1 | 0.0072 | 2.9409 |
| Tuft1 | 0.0031 | 2.9406 |
| Slamf9 | 0.0001 | 2.9402 |
| Sort1 | 0 | 2.9393 |
| Dusp6 | 0 | 2.9345 |
| Gstm5 | 0 | 2.9341 |
| Nt5m | 0 | 2.9322 |
| Lxn | 0 | 2.9246 |
| Notch2 | 0 | 2.9162 |
| 2310045A20Rik | 0.0068 | 2.9143 |
| C1r | 0.0076 | 2.9119 |
| Btg2 | 0.0001 | 2.9073 |
| Mmp23 | 0.0002 | 2.9057 |
| LOC100047469 | 0.0008 | 2.9031 |
| Kif1a | 0.0004 | 2.8958 |
| Lrp2 | 0.0081 | 2.8914 |
| Fbxo10 | 0 | 2.8845 |
| Sparcl1 | 0.0039 | 2.8829 |
| Pcbd1 | 0.0034 | 2.8776 |
| Rab15 | 0.0004 | 2.8674 |
| Matn2 | 0.0037 | 2.8639 |
| Stard13 | 0.0078 | 2.8538 |
| 4632433K11Rik | 0.0026 | 2.8523 |
| Afap1 | 0 | 2.8494 |
| Stxbp4 | 0.0059 | 2.8467 |
| Cldn9 | 0.0058 | 2.8435 |
| Cbfa2t3 | 0.0047 | 2.8431 |
| 1700017B05Rik | 0.0002 | 2.8409 |
| 1700056E22Rik | 0.0007 | 2.826 |
| Adam11 | 0 | 2.8184 |
| Usp29 | 0.0045 | 2.8133 |
| Bicc1 | 0.0015 | 2.8126 |
| Ankrd57 | 0.0075 | 2.8111 |
| Ankrd13b | 0.0004 | 2.8105 |
| Rnpepl1 | 0.0001 | 2.8082 |
| Slc10a6 | 0.0091 | 2.8062 |
| Fbxo4 | 0.0001 | 2.8034 |
| Bend7 | 0.0089 | 2.8034 |
| Olfm2 | 0.0075 | 2.8024 |
| Cd47 | 0.001 | 2.793 |
| Dbx2 | 0 | 2.7868 |
| Gcg | 0.004 | 2.7844 |
| Spef1 | 0 | 2.7777 |
| Gstm2 | 0.0002 | 2.7767 |
| Selm | 0.001 | 2.7731 |
| Klra5 | 0.0053 | 2.7701 |
| Mboat1 | 0.0004 | 2.7696 |
| Fgd3 | 0.0093 | 2.7647 |
| 100043189 | 0 | 2.7598 |
| Cgrrf1 | 0.0021 | 2.7574 |
| Tha1 | 0.0043 | 2.7513 |
| Reep2 | 0.002 | 2.7504 |
| Naglu | 0.0001 | 2.7475 |
| Aph1b | 0.0031 | 2.7372 |
| Vamp8 | 0 | 2.7358 |
| Sdk2 | 0 | 2.731 |
| Fbxo28 | 0.0004 | 2.7306 |
| Oasl2 | 0.0035 | 2.7245 |
| Nfkb2 | 0.0024 | 2.7193 |
| Paqr4 | 0.0003 | 2.7106 |
| Rbm11 | 0.0033 | 2.7002 |
| Tspyl4 | 0.0055 | 2.6996 |
| Arf3 | 0 | 2.6991 |
| Actl7a | 0.0057 | 2.6985 |
| Emp2 | 0.0058 | 2.6975 |
| Nrbp2 | 0.0001 | 2.6955 |
| Plscr3 | 0.0003 | 2.6937 |
| Man2a2 | 0.0017 | 2.6891 |
| Gng2 | 0.0005 | 2.6865 |
| Chst7 | 0.0074 | 2.6825 |
| Zfp787 | 0.0012 | 2.6777 |
| Khdrbs3 | 0.0002 | 2.6711 |
| Olfr1143 | 0.0019 | 2.6686 |
| Tmem37 | 0 | 2.6656 |
| Gpc1 | 0.0021 | 2.663 |
| Creb3 | 0.0001 | 2.6604 |
| Bcl6 | 0.0003 | 2.6602 |
| Ap3b2 | 0.0006 | 2.6578 |
| Lamp2 | 0.0005 | 2.6569 |
| Plagl2 | 0.0012 | 2.6552 |
| Serping1 | 0.0029 | 2.6534 |
| Fam57a | 0.0001 | 2.6483 |
| 2810022L02Rik | 0.0073 | 2.6482 |
| Art5 | 0.0052 | 2.6432 |
| Iqub | 0.0066 | 2.6428 |
| Lhx5 | 0.0059 | 2.6378 |
| 1700010N08Rik | 0.002 | 2.6361 |
| Hspa1l | 0.003 | 2.6274 |
| Srf | 0.0073 | 2.6258 |
| Prokr1 | 0.0003 | 2.6239 |
| Olfr414 | 0.0018 | 2.6218 |
| 1810031K17Rik | 0.0006 | 2.6198 |
| Nepn | 0.0056 | 2.618 |
| Stard10 | 0.0096 | 2.6117 |
| Wars2 | 0.0004 | 2.6113 |
| Disp1 | 0.0053 | 2.607 |
| Hk3 | 0.007 | 2.5982 |
| Ifi27l1 | 0.0003 | 2.5938 |
| Rspo1 | 0 | 2.592 |
| Ccl17 | 0.0064 | 2.5899 |
| Zswim6 | 0.0012 | 2.5831 |
| Mgat4b | 0.0021 | 2.5806 |
| Peg12 | 0.0016 | 2.5686 |
| Bend5 | 0.0003 | 2.568 |
| Ly96 | 0.0004 | 2.563 |
| LOC676546 | 0.0018 | 2.55 |
| Rtp4 | 0.0094 | 2.5459 |
| Cldn6 | 0.0007 | 2.5449 |
| Smad3 | 0.003 | 2.544 |
| Arih1 | 0.0049 | 2.5425 |
| 6720460F02Rik | 0.0007 | 2.5422 |
| H2-Oa | 0.0003 | 2.5421 |
| Cyp3a11 | 0.003 | 2.5371 |
| Klhl38 | 0.0075 | 2.5303 |
| EG433230 | 0.0009 | 2.5291 |
| Dusp10 | 0.0025 | 2.5258 |
| Pcsk9 | 0.0012 | 2.5176 |
| 4930564B18Rik | 0.0015 | 2.517 |
| Ppp2r2c | 0.0008 | 2.5113 |
| Tmem119 | 0.0004 | 2.5023 |
| Efnb2 | 0 | 2.5007 |
| Gng11 | 0 | 2.5 |
| H2-Q5 | 0.0009 | 2.4993 |
| Svop | 0.0011 | 2.4858 |
| Tgfbi | 0.0023 | 2.4854 |
| ENSMUSG00000074747 | 0.0001 | 2.4783 |
| Sox11 | 0.0017 | 2.4782 |
| 2310022B05Rik | 0.0031 | 2.4757 |
| Wipf1 | 0 | 2.4703 |
| 4632428N05Rik | 0.0092 | 2.4671 |
| Rgmb | 0.008 | 2.4577 |
| Purb | 0 | 2.4563 |
| Nppa | 0.0006 | 2.4555 |
| Prrg3 | 0.008 | 2.4466 |
| Ncam1 | 0 | 2.4464 |
| Cotl1 | 0.0002 | 2.4407 |
| Rev3l | 0.0002 | 2.4396 |
| Asb13 | 0 | 2.4293 |
| Zfp287 | 0 | 2.429 |
| Nr2f6 | 0 | 2.4289 |
| Ccdc150 | 0.0013 | 2.4254 |
| Sorbs3 | 0.0081 | 2.4248 |
| Tubb6 | 0 | 2.4225 |
| Glis2 | 0.0007 | 2.4222 |
| Hkdc1 | 0.0003 | 2.4207 |
| Purg | 0.0006 | 2.4191 |
| Wbp1 | 0 | 2.4183 |
| Slc4a7 | 0.0001 | 2.4153 |
| Hmgcll1 | 0.0067 | 2.4091 |
| Cckbr | 0.0052 | 2.4067 |
| Cxcr7 | 0.0001 | 2.4065 |
| 1700025G04Rik | 0.0043 | 2.4019 |
| Zbtb10 | 0.0081 | 2.3987 |
| Fbxw8 | 0.0007 | 2.3936 |
| Crip3 | 0.0007 | 2.392 |
| Serinc5 | 0 | 2.3848 |
| Krt5 | 0.0031 | 2.3845 |
| Commd3 | 0.0013 | 2.3841 |
| Erbb2 | 0.001 | 2.3709 |
| Ddc | 0.0009 | 2.3666 |
| Serf1 | 0.0052 | 2.3627 |
| Katnal2 | 0.0001 | 2.3613 |
| Cyp4v3 | 0.0002 | 2.3584 |
| Khk | 0 | 2.3569 |
| Spag4 | 0 | 2.353 |
| Sfxn4 | 0.0006 | 2.3504 |
| Gm9 | 0.0002 | 2.3463 |
| St3gal6 | 0.0029 | 2.3452 |
|  | 0.0009 | 2.3447 |
| Spon2 | 0.0004 | 2.3434 |
| Ehd3 | 0.0023 | 2.3433 |
| Cyb561d2 | 0.0002 | 2.3432 |
| Apln | 0.0013 | 2.3412 |
| Trim68 | 0.0011 | 2.3342 |
| Snx19 | 0.0042 | 2.3314 |
| Arl2bp | 0.0004 | 2.3262 |
| Lemd2 | 0.0005 | 2.3244 |
| Prkcdbp | 0.0005 | 2.321 |
| Loxl2 | 0.0001 | 2.3207 |
| Klhl30 | 0.0048 | 2.3191 |
| Bcl9l | 0.0064 | 2.316 |
| Mpzl1 | 0 | 2.3122 |
| Atp6v0e2 | 0.0032 | 2.2982 |
| Npnt | 0.0076 | 2.2971 |
| Egfr | 0.0071 | 2.2945 |
| Surf4 | 0.0022 | 2.2937 |
| Ccng1 | 0.0098 | 2.293 |
| Mknk2 | 0.0017 | 2.2903 |
| Sepp1 | 0 | 2.2902 |
| Kbtbd5 | 0.0003 | 2.2873 |
| Snn | 0.0021 | 2.2873 |
| Card10 | 0.0014 | 2.2839 |
| Ddn | 0.0068 | 2.2826 |
| Micall1 | 0 | 2.2822 |
| Phf13 | 0.0012 | 2.2794 |
| Cd151 | 0.0015 | 2.2756 |
| Cxadr | 0.0079 | 2.274 |
| Pbx2 | 0.0004 | 2.2729 |
| Rhoq | 0.0073 | 2.27 |
| Bmpr1a | 0.0062 | 2.2666 |
| Sdc2 | 0.0016 | 2.266 |
| E130119H09Rik | 0.0002 | 2.2658 |
| Ghr | 0.0095 | 2.2649 |
| Atp8a2 | 0.0037 | 2.2611 |
| Anxa5 | 0.0014 | 2.2557 |
| Zdhhc7 | 0.0003 | 2.2539 |
| Efna4 | 0.0019 | 2.2521 |
| B4galt2 | 0.0082 | 2.2519 |
| Ptgr1 | 0.0002 | 2.2499 |
| B3galt1 | 0.0053 | 2.2499 |
| Tbpl1 | 0.0001 | 2.2494 |
| Mecp2 | 0.0001 | 2.246 |
| Dlk2 | 0.0011 | 2.2454 |
| Scamp1 | 0.0022 | 2.2451 |
| Prkg2 | 0.0024 | 2.2435 |
| 1700001K19Rik | 0.0044 | 2.2422 |
| Adcy7 | 0.0002 | 2.2417 |
| 2610035D17Rik | 0.0003 | 2.2315 |
| Chrnb1 | 0.0016 | 2.2312 |
| Tmem47 | 0.0017 | 2.2311 |
| 4930544O15Rik | 0.0022 | 2.2271 |
| Stom | 0.0004 | 2.227 |
| B4galnt1 | 0.0044 | 2.2263 |
| Six5 | 0.0016 | 2.2247 |
| Slc3a1 | 0.0099 | 2.2215 |
| Psmb8 | 0.0024 | 2.216 |
| Map3k7ip2 | 0.0001 | 2.2144 |
| Plxnb1 | 0.005 | 2.2124 |
| Adprhl1 | 0.001 | 2.2104 |
| D430019H16Rik | 0 | 2.2101 |
| Zfp36l1 | 0.0051 | 2.208 |
| Mmp2 | 0.0001 | 2.2065 |
| Ust | 0.0054 | 2.2062 |
| Car1 | 0.0038 | 2.206 |
| Slc16a12 | 0.0039 | 2.2045 |
| Zfp536 | 0.0015 | 2.2027 |
| Smug1 | 0.0002 | 2.2026 |
| Tmem9 | 0.0004 | 2.2018 |
| Kctd17 | 0 | 2.1977 |
| Arhgef12 | 0.002 | 2.197 |
| Map1lc3a | 0.0098 | 2.1969 |
| Golm1 | 0.0007 | 2.1907 |
| Gatsl2 | 0.0003 | 2.1888 |
| 6530418L21Rik | 0.0088 | 2.1886 |
| Hist1h2ba | 0.0002 | 2.1821 |
| 1700001E04Rik | 0.003 | 2.182 |
| Nfe2 | 0.0042 | 2.1811 |
| Spert | 0.001 | 2.175 |
| Glce | 0 | 2.1737 |
| Abca5 | 0.0023 | 2.1734 |
| 2310021H06Rik | 0.0001 | 2.1694 |
| Pkd2 | 0.0026 | 2.168 |
| Yipf3 | 0.0001 | 2.1672 |
| Tmem30a | 0.0001 | 2.1667 |
| Nsg2 | 0.0051 | 2.1655 |
| Megf9 | 0.0071 | 2.1648 |
| LOC100047857 | 0.0004 | 2.1632 |
| Gpd1 | 0.0039 | 2.162 |
| Ldoc1l | 0.0001 | 2.1593 |
| Dgat2 | 0.0058 | 2.1586 |
| Smpdl3a | 0.0069 | 2.1566 |
| Pdlim2 | 0 | 2.1544 |
| Fbxl8 | 0.0004 | 2.1519 |
| Lfng | 0.0027 | 2.151 |
| Rfxap | 0.0029 | 2.147 |
| Btbd17 | 0.0001 | 2.142 |
| Mogs | 0 | 2.1412 |
| Barhl1 | 0.0061 | 2.1403 |
| E2f6 | 0.0002 | 2.1384 |
| P2rx3 | 0.0002 | 2.1378 |
| Lmo4 | 0 | 2.1377 |
| Tmprss8 | 0.0016 | 2.1372 |
| Leprel2 | 0.0029 | 2.1363 |
| 6820408C15Rik | 0.0038 | 2.1351 |
| Klf6 | 0.0001 | 2.1348 |
| 1110032A03Rik | 0.0016 | 2.1331 |
| Aig1 | 0.005 | 2.133 |
| Kif1c | 0.0025 | 2.1297 |
| Ntn3 | 0.0011 | 2.1255 |
| Ctsh | 0.0007 | 2.1244 |
| Vmo1 | 0.0008 | 2.1237 |
| Tmem150 | 0.0039 | 2.1229 |
| Tubb2a | 0.0022 | 2.1206 |
| Prl3b1 | 0.006 | 2.1205 |
| Mapre2 | 0.0018 | 2.1183 |
| Phlppl | 0.0014 | 2.1162 |
| Ankrd42 | 0.0069 | 2.1143 |
| Cdkl1 | 0.0027 | 2.1131 |
| Itm2c | 0.0001 | 2.1117 |
| Rnd1 | 0 | 2.11 |
| Pax7 | 0.003 | 2.1075 |
| Met | 0.001 | 2.1064 |
| Ube2e2 | 0.0001 | 2.105 |
| Drd4 | 0.0001 | 2.1025 |
| Rbx1 | 0.0003 | 2.1022 |
| Mex3a | 0 | 2.102 |
| Ubtd2 | 0.0022 | 2.099 |
| Rraga | 0.0014 | 2.0985 |
| 1700012B09Rik | 0.0048 | 2.0983 |
| St6gal1 | 0.0007 | 2.0961 |
| Tirap | 0 | 2.095 |
| Tnnc1 | 0.006 | 2.094 |
| Ube2j1 | 0.002 | 2.0938 |
| Panx1 | 0.0028 | 2.0896 |
| Klhl26 | 0 | 2.0855 |
| Dtnbp1 | 0.0022 | 2.0853 |
| Snx26 | 0.0098 | 2.0844 |
| 1700022A21Rik | 0.0003 | 2.0786 |
| Mtap2 | 0.0011 | 2.0778 |
| Adamtsl5 | 0.0018 | 2.0752 |
| Guca1a | 0.0013 | 2.0738 |
| Dnajc9 | 0 | 2.073 |
| Sar1a | 0.0003 | 2.0689 |
| Cables1 | 0.0002 | 2.0681 |
| Acr | 0.0093 | 2.0658 |
| Prox2 | 0.0061 | 2.0643 |
| EG629689 | 0.0011 | 2.0622 |
| Zfp385a | 0.0005 | 2.062 |
| Suox | 0.0001 | 2.0599 |
| Igfbp4 | 0.0078 | 2.0596 |
| Olfr1395 | 0.004 | 2.0565 |
| 1500015O10Rik | 0.0045 | 2.0547 |
| Lrp1 | 0.0005 | 2.0534 |
| Tchh | 0.0005 | 2.0495 |
| Rnf24 | 0.0054 | 2.0483 |
| Fabp2 | 0.0057 | 2.0458 |
| Snx21 | 0 | 2.0435 |
| Il4 | 0.005 | 2.0401 |
| Eif1b | 0 | 2.0366 |
| 2410022M11Rik | 0 | 2.0358 |
| Ddah2 | 0.0015 | 2.0347 |
| F2r | 0.0001 | 2.0327 |
| Des | 0 | 2.025 |
| Bace1 | 0.0015 | 2.0239 |
| Psd | 0.0047 | 2.0222 |
| Magea7 | 0.0027 | 2.0204 |
| Dok1 | 0.0016 | 2.0177 |
| Eif5a2 | 0.0002 | 2.0146 |
| Chst12 | 0.0002 | 2.0124 |
| Qk | 0.0083 | 2.0113 |
| Hist1h3a | 0.0007 | 2.0094 |
| Ubxn4 | 0.0006 | 2.0093 |
| Hapln3 | 0.0014 | 2.0074 |
| Cd52 | 0.0056 | 2.0033 |
| Stx17 | 0.0004 | 2.0028 |
| Tpcn1 | 0.0023 | 2.0011 |
| Sla | 0.0088 | 2.0005 |
