# Supplementary material for: Retinoic Acid Induces Embryonic Stem Cell Differentiation by Altering Both Encoding RNA and microRNA Expression
Source: PLoS One. 2015 Jul 10;10(7):e0132566. doi: 10.1371/journal.pone.0132566 (PMC4498831; doi:10.1371/journal.pone.0132566)
Supplement: S3 Table — Fold change values were provided in comparison with J1 mESCs treated by DMSO standard. (DOC) [file pone.0132566.s004.doc]

**Table S3 Significantly down-regulated microRNA in RA treated J1 mESCs. (FC<0.4, p < 0.01)**

Fold change values were provided in comparison with J1 mESCs treated by DMSO-std.

| miR-name | DMSO-std | RA-std | fold change | p-value |
| --- | --- | --- | --- | --- |
| mmu-let-7a-5p | 1810.089 | 492.1244 | 0.271878595 | 0 |
| mmu-let-7b-5p | 90.6714 | 30.7126 | 0.338724229 | 5.9175E-131 |
| mmu-let-7d-5p | 147.6921 | 35.3582 | 0.239404816 | 9.49949098e-311 |
| mmu-let-7e-5p | 946.8825 | 326.3279 | 0.344633997 | 0 |
| mmu-let-7f-5p | 2775.731 | 886.7426 | 0.31946274 | 0 |
| mmu-let-7g-5p | 857.483 | 171.4744 | 0.199974111 | 0 |
| mmu-let-7i-5p | 46.3691 | 14.8143 | 0.319486468 | 4.16205E-73 |
| mmu-miR-101a-3p | 1245.022 | 280.1299 | 0.224999905 | 0 |
| mmu-miR-101b-3p | 2609.597 | 774.7836 | 0.296897774 | 0 |
| mmu-miR-103-3p | 6143.078 | 2243.93 | 0.365277835 | 0 |
| mmu-miR-106b-3p | 128.4025 | 37.2681 | 0.290244348 | 6.0061E-223 |
| mmu-miR-106b-5p | 127.3957 | 47.7981 | 0.375193981 | 2.6565E-158 |
| mmu-miR-107-3p | 1743.794 | 553.7044 | 0.317528506 | 0 |
| mmu-miR-1186 | 16.4279 | 3.8197 | 0.232512981 | 4.93214E-37 |
| mmu-miR-1188-5p | 18.7596 | 2.9422 | 0.156837033 | 2.03834E-55 |
| mmu-miR-1197-3p | 5.5113 | 1.9615 | 0.355905139 | 7.61446E-09 |
| mmu-miR-1198-5p | 3.7095 | 0.8775 | 0.236554792 | 2.49571E-09 |
| mmu-miR-124-3p | 255.1623 | 35.5131 | 0.139178476 | 0 |
| mmu-miR-124-5p | 2.2787 | 0.3097 | 0.135910827 | 1.95232E-08 |
| mmu-miR-125a-3p | 2.3317 | 0.8259 | 0.354205085 | 0.000177351 |
| mmu-miR-125a-5p | 41.5997 | 15.3305 | 0.368524293 | 2.38095E-54 |
| mmu-miR-126-3p | 1.2718 | 0.1032 | 0.081144834 | 4.15574E-06 |
| mmu-miR-126-5p | 2.3847 | 0.7226 | 0.303015055 | 2.73139E-05 |
| mmu-miR-128-3p | 975.9758 | 273.368 | 0.280097109 | 0 |
| mmu-miR-129-1-3p | 3.4976 | 0.6194 | 0.177092863 | 9.82158E-11 |
| mmu-miR-1306-3p | 21.8332 | 5.4199 | 0.24824121 | 5.77681E-46 |
| mmu-miR-1306-5p | 9.9097 | 3.8197 | 0.385450619 | 2.77924E-13 |
| mmu-miR-130a-3p | 639.7869 | 239.1454 | 0.373789148 | 0 |
| mmu-miR-130b-3p | 251.9827 | 63.1286 | 0.250527516 | 0 |
| mmu-miR-130b-5p | 7.949 | 1.4969 | 0.188312996 | 8.34024E-22 |
| mmu-miR-139-3p | 8.9029 | 2.2196 | 0.249312022 | 1.38193E-19 |
| mmu-miR-140-3p | 7632.982 | 2147.095 | 0.281291788 | 0 |
| mmu-miR-140-5p | 1.3778 | 0.2581 | 0.187327623 | 8.40108E-05 |
| mmu-miR-141-3p | 1.0069 | 0.2065 | 0.205084914 | 0.001241774 |
| mmu-miR-142-5p | 15.315 | 3.2003 | 0.208965067 | 1.06971E-37 |
| mmu-miR-143-3p | 100.7401 | 35.6163 | 0.353546404 | 5.351E-137 |
| mmu-miR-143-5p | 8.4789 | 1.755 | 0.206984397 | 1.06947E-21 |
| mmu-miR-146b-5p | 106.4103 | 38.5069 | 0.361871925 | 7.2572E-140 |
| mmu-miR-148a-3p | 34.4456 | 12.8528 | 0.373133289 | 1.7471E-44 |
| mmu-miR-148b-3p | 77.1051 | 24.9314 | 0.323343073 | 1.4761E-118 |
| mmu-miR-148b-5p | 4.2395 | 0.9291 | 0.219153202 | 4.34994E-11 |
| mmu-miR-149-3p | 13.2483 | 1.2388 | 0.093506337 | 3.59538E-50 |
| mmu-miR-150-3p | 3.4446 | 0.1549 | 0.044968937 | 8.05331E-17 |
| mmu-miR-150-5p | 12.3474 | 2.6841 | 0.217381797 | 8.68737E-30 |
| mmu-miR-155-5p | 1.9607 | 0.1549 | 0.079002397 | 6.57728E-09 |
| mmu-miR-16-1-3p | 25.7017 | 8.0524 | 0.313302234 | 2.89966E-42 |
| mmu-miR-16-2-3p | 2.6497 | 0.8775 | 0.331169565 | 2.79926E-05 |
| mmu-miR-17-3p | 90.2474 | 30.0932 | 0.333452266 | 4.4541E-133 |
| mmu-miR-17-5p | 115.7372 | 39.6425 | 0.34252168 | 3.9052E-164 |
| mmu-miR-181c-3p | 36.3003 | 5.4715 | 0.150728782 | 4.1965E-108 |
| mmu-miR-181c-5p | 138.3652 | 27.5123 | 0.198838292 | 0 |
| mmu-miR-181d-3p | 1.9078 | 0.1032 | 0.054093721 | 1.78483E-09 |
| mmu-miR-181d-5p | 2381.462 | 335.361 | 0.140821508 | 0 |
| mmu-miR-182-3p | 6.5712 | 0.3613 | 0.054982347 | 9.58503E-30 |
| mmu-miR-182-5p | 1005.228 | 203.9937 | 0.202932767 | 0 |
| mmu-miR-183-3p | 40.5398 | 8.4653 | 0.208814548 | 4.3931E-97 |
| mmu-miR-183-5p | 181.9257 | 38.2488 | 0.210244072 | 0 |
| mmu-miR-1839-5p | 918.3192 | 302.5321 | 0.329441114 | 0 |
| mmu-miR-185-5p | 963.6814 | 253.3403 | 0.262888025 | 0 |
| mmu-miR-186-3p | 2.4377 | 0.5678 | 0.232924479 | 1.25024E-06 |
| mmu-miR-187-3p | 21.0383 | 3.8713 | 0.184012016 | 4.63237E-56 |
| mmu-miR-187-5p | 7.3661 | 0.671 | 0.09109298 | 8.55134E-29 |
| mmu-miR-18a-3p | 1.9607 | 0.7743 | 0.394909982 | 0.001588916 |
| mmu-miR-18b-5p | 15.209 | 5.7812 | 0.380117036 | 5.33053E-20 |
| mmu-miR-191-3p | 1.6958 | 0.2581 | 0.152199552 | 2.92345E-06 |
| mmu-miR-191-5p | 2471.603 | 772.0994 | 0.312388128 | 0 |
| mmu-miR-192-5p | 581.1235 | 119.2888 | 0.205272718 | 0 |
| mmu-miR-1929-5p | 2.6497 | 0.9807 | 0.370117373 | 0.000106922 |
| mmu-miR-1930-5p | 2.2257 | 0.6194 | 0.278294469 | 2.16921E-05 |
| mmu-miR-1934-3p | 8.9029 | 1.084 | 0.121758079 | 9.02715E-31 |
| mmu-miR-1935 | 57.9216 | 2.0647 | 0.03564646 | 7.2147E-274 |
| mmu-miR-193-5p | 1.1659 | 0.2581 | 0.221374045 | 0.000713956 |
| mmu-miR-1938 | 1.7488 | 0.5162 | 0.295173833 | 0.00027944 |
| mmu-miR-193b-5p | 7.419 | 0.8259 | 0.11132228 | 6.91871E-27 |
| mmu-miR-1943-5p | 2.5967 | 0.2581 | 0.099395387 | 1.17322E-10 |
| mmu-miR-194-5p | 5.0344 | 1.0324 | 0.205069125 | 1.53856E-13 |
| mmu-miR-1947-5p | 4.7164 | 0.9291 | 0.19699347 | 4.07203E-13 |
| mmu-miR-195-5p | 15.686 | 6.1941 | 0.394880784 | 1.90708E-19 |
| mmu-miR-1964-3p | 1.9078 | 0.5678 | 0.297620296 | 0.000155765 |
| mmu-miR-1965 | 126.6538 | 11.8205 | 0.093329217 | 0 |
| mmu-miR-1968-5p | 16.4809 | 1.9615 | 0.119016558 | 2.9379E-56 |
| mmu-miR-196a-1-3p | 10.3337 | 1.6002 | 0.15485257 | 1.89514E-31 |
| mmu-miR-196a-5p | 43.5604 | 7.9491 | 0.18248455 | 6.9808E-115 |
| mmu-miR-1981-5p | 225.6451 | 59.4637 | 0.263527548 | 0 |
| mmu-miR-19a-3p | 1.2718 | 0.4129 | 0.324657965 | 0.003594005 |
| mmu-miR-200a-3p | 48.5948 | 17.2404 | 0.3547787 | 1.01151E-66 |
| mmu-miR-200b-3p | 39.109 | 13.6787 | 0.349758368 | 4.13216E-55 |
| mmu-miR-200b-5p | 10.3337 | 2.3744 | 0.229772493 | 4.22667E-24 |
| mmu-miR-200c-3p | 20.6144 | 5.6263 | 0.272930572 | 9.85154E-40 |
| mmu-miR-205-5p | 7.1541 | 1.5485 | 0.216449309 | 6.14992E-18 |
| mmu-miR-20a-5p | 55.5899 | 20.4407 | 0.367705286 | 2.88225E-72 |
| mmu-miR-20b-3p | 1.9078 | 0.1549 | 0.081192997 | 1.23793E-08 |
| mmu-miR-210-5p | 8.002 | 1.9615 | 0.245126219 | 5.19727E-18 |
| mmu-miR-23a-3p | 256.4341 | 65.0901 | 0.253827787 | 0 |
| mmu-miR-23a-5p | 6.0412 | 0.6194 | 0.102529299 | 7.16827E-23 |
| mmu-miR-23b-3p | 127.2367 | 23.641 | 0.18580331 | 0 |
| mmu-miR-23b-5p | 48.5948 | 4.8005 | 0.09878629 | 5.4699E-176 |
| mmu-miR-24-3p | 179.488 | 41.191 | 0.229491665 | 0 |
| mmu-miR-25-3p | 5232.443 | 1329.572 | 0.254101583 | 0 |
| mmu-miR-25-5p | 880.2171 | 138.3358 | 0.157161 | 0 |
| mmu-miR-27a-3p | 194.3791 | 36.6487 | 0.188542389 | 0 |
| mmu-miR-27b-3p | 243.1328 | 45.785 | 0.188312724 | 0 |
| mmu-miR-27b-5p | 49.6546 | 5.0585 | 0.101873743 | 1.3115E-177 |
| mmu-miR-290-3p | 18.1767 | 0.9807 | 0.053953688 | 3.36156E-80 |
| mmu-miR-290-5p | 10977.33 | 1603.818 | 0.146102697 | 0 |
| mmu-miR-291a-3p | 481.4962 | 163.2156 | 0.338975883 | 0 |
| mmu-miR-291a-5p | 1459.592 | 266.2964 | 0.182445804 | 0 |
| mmu-miR-291b-3p | 52.9402 | 15.7434 | 0.297380819 | 1.47429E-90 |
| mmu-miR-291b-5p | 238.5754 | 55.8505 | 0.234099995 | 0 |
| mmu-miR-292-3p | 1359.488 | 369.1707 | 0.271551356 | 0 |
| mmu-miR-292-5p | 595.6966 | 87.8535 | 0.147480277 | 0 |
| mmu-miR-293-3p | 11548.6 | 2134.759 | 0.184850037 | 0 |
| mmu-miR-293-5p | 741.0569 | 179.0106 | 0.241561208 | 0 |
| mmu-miR-294-3p | 2896.026 | 886.1748 | 0.305996891 | 0 |
| mmu-miR-294-5p | 392.8916 | 76.5492 | 0.19483542 | 0 |
| mmu-miR-295-3p | 2236.843 | 445.3586 | 0.199101412 | 0 |
| mmu-miR-295-5p | 348.4303 | 55.4892 | 0.159254806 | 0 |
| mmu-miR-296-3p | 139.3191 | 17.7049 | 0.127081642 | 0 |
| mmu-miR-296-5p | 72.9186 | 26.9961 | 0.370222412 | 2.82586E-93 |
| mmu-miR-298-5p | 846.2484 | 203.4775 | 0.24044654 | 0 |
| mmu-miR-29a-3p | 304.9759 | 111.0816 | 0.364230747 | 0 |
| mmu-miR-300-3p | 10.2807 | 3.4068 | 0.331378213 | 1.01571E-16 |
| mmu-miR-301a-5p | 62.002 | 22.8151 | 0.367973613 | 2.6293E-80 |
| mmu-miR-301b-5p | 1.7488 | 0.2581 | 0.147586917 | 1.64738E-06 |
| mmu-miR-302a-3p | 1.3778 | 0.4129 | 0.29968065 | 0.001445573 |
| mmu-miR-302a-5p | 9.0618 | 1.4453 | 0.159493698 | 2.64597E-27 |
| mmu-miR-302b-3p | 1.4308 | 0.5162 | 0.360777187 | 0.003964096 |
| mmu-miR-3057-5p | 3.1796 | 0.3613 | 0.113630646 | 3.59926E-12 |
| mmu-miR-3062-5p | 2.9676 | 0.6194 | 0.208720851 | 2.0456E-08 |
| mmu-miR-3064-5p | 3.8685 | 1.084 | 0.280211968 | 2.07996E-08 |
| mmu-miR-3066-5p | 12.6124 | 4.0262 | 0.319225523 | 4.34556E-21 |
| mmu-miR-3068-3p | 1.6428 | 0.5678 | 0.345629413 | 0.001430666 |
| mmu-miR-3068-5p | 1.0599 | 0.4129 | 0.389565054 | 0.020038691 |
| mmu-miR-3078-5p | 1.9607 | 0.3097 | 0.157953792 | 6.14113E-07 |
| mmu-miR-3079-5p | 1.2188 | 0.0516 | 0.042336725 | 1.14168E-06 |
| mmu-miR-3086-5p | 1.1659 | 0.2065 | 0.17711639 | 0.000240379 |
| mmu-miR-3095-3p | 83.4643 | 24.312 | 0.291286214 | 4.9315E-145 |
| mmu-miR-30c-1-3p | 6.7301 | 1.755 | 0.260768785 | 1.41845E-14 |
| mmu-miR-30c-2-3p | 29.7822 | 11.4592 | 0.38476674 | 5.89682E-37 |
| mmu-miR-30c-5p | 31.054 | 11.7172 | 0.377316933 | 1.29961E-39 |
| mmu-miR-30e-3p | 11.7645 | 4.3875 | 0.372944025 | 3.08438E-16 |
| mmu-miR-3102-5p.2-5p | 7.1011 | 2.839 | 0.399797216 | 2.02705E-09 |
| mmu-miR-320-3p | 3850.115 | 1162.692 | 0.301988764 | 0 |
| mmu-miR-323-3p | 1378.406 | 442.3132 | 0.320887414 | 0 |
| mmu-miR-323-5p | 34.1276 | 10.5817 | 0.310062823 | 2.99546E-56 |
| mmu-miR-324-3p | 1.7488 | 0.5678 | 0.324679781 | 0.000600372 |
| mmu-miR-32-5p | 12.5594 | 3.6132 | 0.287688901 | 1.52327E-23 |
| mmu-miR-328-5p | 1.4838 | 0.4646 | 0.313114974 | 0.001258292 |
| mmu-miR-329-5p | 19.2365 | 6.6587 | 0.346149248 | 2.46948E-28 |
| mmu-miR-330-3p | 27.9274 | 10.4784 | 0.375201414 | 4.32396E-36 |
| mmu-miR-331-3p | 6.8891 | 2.0131 | 0.292215239 | 2.30458E-13 |
| mmu-miR-341-3p | 238.2044 | 67.0516 | 0.281487663 | 0 |
| mmu-miR-342-5p | 16.4809 | 6.1425 | 0.372704161 | 3.66762E-22 |
| mmu-miR-345-3p | 45.3622 | 14.5046 | 0.319750806 | 1.7392E-71 |
| mmu-miR-3470a | 20.1374 | 7.5878 | 0.376801374 | 2.59553E-26 |
| mmu-miR-3470b | 9.9097 | 2.7357 | 0.276062848 | 1.09593E-19 |
| mmu-miR-3474 | 2.4907 | 0.8259 | 0.331593527 | 4.99232E-05 |
| mmu-miR-34a-3p | 5.6703 | 1.2388 | 0.218471686 | 2.07711E-14 |
| mmu-miR-361-3p | 1.0599 | 0.1549 | 0.146145862 | 0.000217502 |
| mmu-miR-361-5p | 10.0157 | 3.1487 | 0.314376429 | 2.36764E-17 |
| mmu-miR-363-3p | 147.2681 | 45.9399 | 0.311947393 | 7.2822E-235 |
| mmu-miR-363-5p | 974.333 | 307.6939 | 0.315799526 | 0 |
| mmu-miR-365-1-5p | 2.3317 | 0.7226 | 0.309902646 | 4.27833E-05 |
| mmu-miR-365-2-5p | 23.37 | 7.433 | 0.318057339 | 6.73224E-38 |
| mmu-miR-377-5p | 14.5731 | 5.5231 | 0.378992801 | 2.61396E-19 |
| mmu-miR-378-3p | 264.2771 | 44.8043 | 0.169535309 | 0 |
| mmu-miR-381-3p | 1.7488 | 0.671 | 0.383691676 | 0.002302843 |
| mmu-miR-382-3p | 317.7472 | 119.1339 | 0.374932965 | 0 |
| mmu-miR-382-5p | 847.4673 | 328.1861 | 0.387255177 | 0 |
| mmu-miR-409-3p | 197.8766 | 79.0785 | 0.399635431 | 2.8511E-221 |
| mmu-miR-410-5p | 8.7969 | 2.3228 | 0.264047563 | 2.12887E-18 |
| mmu-miR-423-3p | 73.6606 | 23.0216 | 0.312536146 | 3.4649E-118 |
| mmu-miR-423-5p | 4282.487 | 1127.488 | 0.263278862 | 0 |
| mmu-miR-425-3p | 8.3729 | 2.6325 | 0.314407195 | 9.63877E-15 |
| mmu-miR-425-5p | 10.9166 | 4.3359 | 0.397184104 | 7.27731E-14 |
| mmu-miR-431-3p | 27.3445 | 10.6333 | 0.388864306 | 1.61176E-33 |
| mmu-miR-433-5p | 25.3308 | 7.3297 | 0.2893592 | 1.39901E-45 |
| mmu-miR-485-3p | 233.2231 | 89.5053 | 0.38377545 | 3.8474E-278 |
| mmu-miR-494-5p | 1.0599 | 0.2581 | 0.243513539 | 0.002003024 |
| mmu-miR-497-5p | 21.0383 | 8.2072 | 0.390107565 | 4.92278E-26 |
| mmu-miR-505-5p | 16.6399 | 3.2003 | 0.192326877 | 2.36179E-43 |
| mmu-miR-5099 | 1.1659 | 0.1549 | 0.132858736 | 6.72567E-05 |
| mmu-miR-5105 | 114.5713 | 20.8536 | 0.182014169 | 1.3464E-299 |
| mmu-miR-5113 | 1.1129 | 0.3097 | 0.278281965 | 0.002982865 |
| mmu-miR-5117-3p | 2.3317 | 0.3097 | 0.132821547 | 1.08844E-08 |
| mmu-miR-5128 | 21.2503 | 4.3875 | 0.206467673 | 3.75837E-52 |
| mmu-miR-539-5p | 6.7831 | 2.2196 | 0.327225016 | 1.10603E-11 |
| mmu-miR-540-3p | 35.6114 | 9.0331 | 0.253657536 | 2.00257E-72 |
| mmu-miR-540-5p | 12.7714 | 4.7488 | 0.371830809 | 1.42457E-17 |
| mmu-miR-541-3p | 3.0206 | 0.671 | 0.222141296 | 3.42464E-08 |
| mmu-miR-542-3p | 4.9814 | 1.9099 | 0.383406271 | 2.10703E-07 |
| mmu-miR-543-3p | 553.514 | 157.5892 | 0.284706801 | 0 |
| mmu-miR-582-3p | 2.0137 | 0.7226 | 0.358841932 | 0.00056793 |
| mmu-miR-664-5p | 13.6193 | 2.5293 | 0.185714391 | 1.24334E-36 |
| mmu-miR-665-3p | 70.534 | 26.3251 | 0.373225677 | 3.33757E-89 |
| mmu-miR-666-5p | 19.5545 | 5.3683 | 0.274530158 | 1.48763E-37 |
| mmu-miR-667-5p | 2.4377 | 0.9291 | 0.381137958 | 0.000282775 |
| mmu-miR-668-3p | 8.2139 | 1.9099 | 0.232520484 | 2.8259E-19 |
| mmu-miR-672-5p | 762.8371 | 97.2996 | 0.127549643 | 0 |
| mmu-miR-673-5p | 4.2924 | 1.4969 | 0.348732643 | 2.38968E-07 |
| mmu-miR-674-5p | 194.856 | 47.4884 | 0.243710227 | 0 |
| mmu-miR-676-5p | 4.6104 | 1.3421 | 0.291102725 | 1.99097E-09 |
| mmu-miR-708-3p | 26.0727 | 4.2327 | 0.16234222 | 7.8771E-75 |
| mmu-miR-708-5p | 237.4625 | 27.5639 | 0.116076854 | 0 |
| mmu-miR-744-5p | 1005.864 | 329.2185 | 0.327299218 | 0 |
| mmu-miR-758-5p | 16.2159 | 6.1941 | 0.381976948 | 4.51769E-21 |
| mmu-miR-760-3p | 20.8263 | 6.7619 | 0.324680812 | 4.04693E-33 |
| mmu-miR-7a-5p | 145.8903 | 37.681 | 0.258283107 | 7.7261E-286 |
| mmu-miR-877-5p | 85.3191 | 8.5686 | 0.100430032 | 1.3685E-305 |
| mmu-miR-92a-1-5p | 77.688 | 17.4984 | 0.225239419 | 2.3167E-173 |
| mmu-miR-92a-2-5p | 280.758 | 44.7527 | 0.159399554 | 0 |
| mmu-miR-92a-3p | 1577.608 | 523.6629 | 0.331934824 | 0 |
| mmu-miR-92b-3p | 288.0181 | 113.5592 | 0.394277998 | 0 |
| mmu-miR-92b-5p | 101.164 | 32.2095 | 0.318388952 | 5.7581E-158 |
| mmu-miR-96-3p | 1.7488 | 0.1549 | 0.088575023 | 8.15858E-08 |
| mmu-miR-96-5p | 123.5801 | 20.5955 | 0.166657091 | 0 |
