# Supplementary material for: Retinoic Acid Induces Embryonic Stem Cell Differentiation by Altering Both Encoding RNA and microRNA Expression
Source: PLoS One. 2015 Jul 10;10(7):e0132566. doi: 10.1371/journal.pone.0132566 (PMC4498831; doi:10.1371/journal.pone.0132566)
Supplement: S4 Table — Fold change values were provided in comparison with J1 mESCs treated by DMSO-std. (DOC) [file pone.0132566.s005.doc]

**Table S4 Significantly up-regulated microRNA in RA treated J1 mESCs. (FC>1.5, p < 0.01)**

Fold change values were provided in comparison with J1 mESCs treated by DMSO-std.

| miR-name | DMSO-std | RA-std | fold change | p-value |
| --- | --- | --- | --- | --- |
| mmu-miR-100-5p | 0.318 | 1.6518 | 5.19433964 | 2.04314E-05 |
| mmu-miR-10a-3p | 0.159 | 7.9491 | 49.99433974 | 2.62087E-41 |
| mmu-miR-10a-5p | 55.3249 | 2154.27 | 38.93852499 | 0 |
| mmu-miR-10b-5p | 0.7949 | 3.0455 | 3.831299531 | 2.87401E-07 |
| mmu-miR-1194 | 1.0599 | 7.2265 | 6.818096033 | 6.35428E-23 |
| mmu-miR-135a-2-3p | 0.212 | 2.9938 | 14.12169815 | 2.83818E-13 |
| mmu-miR-138-5p | 7.3661 | 26.067 | 3.538779007 | 8.31192E-48 |
| mmu-miR-181a-5p | 52.5163 | 173.9005 | 3.311362377 | 1.8115E-286 |
| mmu-miR-188-5p | 0.5299 | 1.5485 | 2.92224948 | 0.001925244 |
| mmu-miR-216b-5p | 0.01 | 1.5485 | 154.8499997 | 1.39622E-09 |
| mmu-miR-217-5p | 0.01 | 12.8528 | 1285.279999 | 2.89536E-74 |
| mmu-miR-219-2-3p | 9.5388 | 151.4983 | 15.88232271 | 0 |
| mmu-miR-219-5p | 2.7556 | 18.9954 | 6.893380733 | 3.61268E-58 |
| mmu-miR-224-5p | 0.212 | 1.1356 | 5.356603758 | 0.0003999 |
| mmu-miR-344d-3p | 0.106 | 1.084 | 10.22641512 | 4.66852E-05 |
| mmu-miR-465a-3p | 6.2002 | 17.9114 | 2.888842299 | 2.25574E-26 |
| mmu-miR-465b-3p | 6.2002 | 17.9114 | 2.888842299 | 2.25574E-26 |
| mmu-miR-465c-3p | 6.2002 | 17.8598 | 2.88051999 | 3.33745E-26 |
| mmu-miR-471-5p | 0.318 | 1.9615 | 6.168238973 | 8.36513E-07 |
| mmu-miR-483-5p | 0.5829 | 3.2519 | 5.578830001 | 6.23205E-10 |
| mmu-miR-615-3p | 0.01 | 1.1872 | 118.7200003 | 1.631E-07 |
| mmu-miR-615-5p | 0.01 | 3.1487 | 314.8700005 | 9.74713E-19 |
| mmu-miR-741-3p | 0.9009 | 2.7874 | 3.094017099 | 1.34763E-05 |
| mmu-miR-741-5p | 0.265 | 1.1356 | 4.285283021 | 0.001160153 |
| mmu-miR-743a-3p | 0.212 | 1.084 | 5.113207559 | 0.000677821 |
| mmu-miR-743b-3p | 0.9539 | 3.4068 | 3.571443554 | 1.60492E-07 |
| mmu-miR-871-3p | 0.265 | 2.1163 | 7.986037741 | 3.99509E-08 |
| mmu-miR-881-3p | 1.7488 | 5.7296 | 3.276303756 | 8.33194E-11 |
| mmu-miR-883b-5p | 0.4239 | 1.9099 | 4.505543757 | 1.37518E-05 |
| mmu-miR-99a-5p | 0.212 | 5.7296 | 27.02641502 | 7.32402E-28 |
