# Supplementary material for: Retinoic Acid Induces Embryonic Stem Cell Differentiation by Altering Both Encoding RNA and microRNA Expression
Source: PLoS One. 2015 Jul 10;10(7):e0132566. doi: 10.1371/journal.pone.0132566 (PMC4498831; doi:10.1371/journal.pone.0132566)
Supplement: S5 Table — All primers used for detecting mRNA expression levels by real-time PCR. (DOC) [file pone.0132566.s006.doc]

**Table S5 Real-time PCR primers.**

All primers used for detecting mRNA expression levels by real-time PCR.

| Gene name | Forward primer | Reverse primer | Amplicon Size |
| --- | --- | --- | --- |
| Brachyury | GCTTCAAGGAGCTAACTAACGAG | CCAGCAAGAAAGAGTACATGGC | 117 |
| Dnmt3b | CGTTAATGGGAACTTCAGTGACC | CTGCGTGTAATTCAGAAGGCT | 169 |
| Dnmt3l | CACCCCTTGTTTGAGGGAGG | ATGGTGCAGTAACTCTGGTGT | 101 |
| Esrrb | CACCCACCCATGCTAGTCTT | ACCCTCAAACTCCTGGTCCT | 150 |
| GAPDH | GTGTTCCTACCCCCAATGTGT | ATTGTCATACCAGGAAATGAGCTT | 248 |
| Gata6 | TTGCTCCGGTAACAGCAGTG | GTGGTCGCTTGTGTAGAAGGA | 105 |
| Hdac2 | GGAGGAGGCTACACAATCCG | TCTGGAGTGTTCTGGTTTGTCA | 173 |
| Hdac8 | ACTATTGCCGGAGATCCAATGT | CCTCCTAAAATCAGAGTTGCCAG | 107 |
| Hoxb1 | GCCCCAACCTCTTTTCCCC | GACAGGATACCCCGAGTTTTG | 117 |
| Hoxb2 | ATTCGCCTTTTCTACCGGACC | GGGCTATCGAGAGAACCCTG | 100 |
| Hoxb5 | CCTTCTCGGGGCGTTATCC | CCTGAAGCGGGGTTCCTTG | 236 |
| Klf4 | GGCGAGTCTGACATGGCTG | GCTGGACGCAGTGTCTTCTC | 95 |
| Lefty1 | ACTCAGTATGTGGCCCTGCTA | AACCTGCCTGCCACCTCT | 92 |
| Lefty2 | CACAAGTTGGTCCGTTTCG | GGTACCTCGGGGTCACAAT | 128 |
| Nanog | CACCCACCCATGCTAGTCTT | ACCCTCAAACTCCTGGTCCT | 150 |
| Nestin | CCCTGAAGTCGAGGAGCTG | CTGCTGCACCTCTAAGCGA | 166 |
| Ntf3 | GGAGTTTGCCGGAAGACTCTC | GGGTGCTCTGGTAATTTTCCTTA | 117 |
| Oct4 | AGAGGGAACCTCCTCTGAGC | TTCTAGCTCCTTCTGCAGGG | 115 |
| Pax6 | TACCAGTGTCTACCAGCCAAT | TGCACGAGTATGAGGAGGTCT | 194 |
| Sox7 | CAAACAAACACCCTGTGACACCCA | ATACACGTGTCCAAGGGCAGACAA | 139 |
| Sox17 | ACGCTAGCTCAGCGGTCTACTATT | AGGGATTTCCTTAGCGCTTCCAGG | 113 |
| Tet1 | ACACAGTGGTGCTAATGCAG | AGCATGAACGGGAGAATCGG | 112 |
| Tet2 | AGAGAAGACAATCGAGAAGTCGG | CCTTCCGTACTCCCAAACTCAT | 104 |
| Tet3 | TGCGATTGTGTCGAACAAATAGT | TCCATACCGATCCTCCATGAG | 111 |
| Tgfb1 | GTGTGGAGCAACATGTGGAACTCTA | CGCTGAATCGAAAGCCCTGTA | 174 |
| Tgfb2 | TCGACATGGATCAGTTTATGCG | CCCTGGTACTGTTGTAGATGGA | 147 |
| Hdac7 | GAACTCTTGAGCCCTTGGACA | GGTGTGCTGCTACTACTGGG | 251 |
