# Supplementary material for: Retinoic Acid Induces Embryonic Stem Cell Differentiation by Altering Both Encoding RNA and microRNA Expression
Source: PLoS One. 2015 Jul 10;10(7):e0132566. doi: 10.1371/journal.pone.0132566 (PMC4498831; doi:10.1371/journal.pone.0132566)
Supplement: S6 Table — List of all primers used for detecting miRNA expression levels by real-time PCR. (DOC) [file pone.0132566.s007.doc]

**Table S6 MicroRNA Real-time PCR primers.**

List of all primers used for detecting microRNA expression levels by real-time PCR.

| Forward Primer name* | Sequence |
| --- | --- |
| mmu-miR-200b-3p | GGGTAATACTGCCTGGTAATGATGA |
| mmu-miR-200c-3p | TAATACTGCCGGGTAATGATGGA |
| mmu-miR-193b-3p | CGGGGTTTTGAGGGCG |
| mmu-miR-200a-3p | GGGTAACACTGTCTGGTAACGATGT |
| mmu-miR-141-3p | GGGTAACACTGTCTGGTAAAGATGG |
| mmu-miR-130-3p | GGACTCTTTCCCTGTTGCACTACT |
| mmu-miR-302a-3p | TAAGTGCTTCCATGTTTTG |
| mmu-miR-449a-5p | GGGTGGCAGTGTATTGT |
| mmu-miR-10a-5p | TACCCTGTAGATCCGAATTTGTG |
| mmu-miR-470-5p | TTCTTGGACTGGCACTGGTGA |
| mmu-miR-181b-5p | AACATTCATTGCTGTCGGTGGGT |
| mmu-miR-135-5p | TATGGCTTTTTATTCCTATGTGA |

*The reverse primer is obtained from miScript II RT Kit 10× miScript Universal Primer (Qiagen)
