# Supplementary material for: Retinoic Acid Induces Embryonic Stem Cell Differentiation by Altering Both Encoding RNA and microRNA Expression
Source: PLoS One. 2015 Jul 10;10(7):e0132566. doi: 10.1371/journal.pone.0132566 (PMC4498831; doi:10.1371/journal.pone.0132566)
Supplement: S7 Table — All primers used for plasmid construction. (DOC) [file pone.0132566.s008.doc]

**Table S7 Sequences of miR-200b and miR-200c primer.**

All primers used for plasmid construct.

| Primer | Sequence |
| --- | --- |
| Mir-200a- EcoR1-R | CGGAATTCCTGTTCATGGCATCAGGT |
| Mir-200a-BamH1-R | CGGGATCCAGTAATAGACAAGGGTCAAAGT |
| Mir-200b-EcoR1-F | CGGAATTCCAGGGCTTTCTGCTGTTG |
| Mir-200b-BamH1-R | CGGGATCCCATATCGCCAAGGCTAACT |
| Mir-200c-EcoR1-F | CGGAATTCGGAAGTGTCCCAAATGACG |
| Mir-200c-BamH1-R | CGGGATCCTTATGTGTGGGAGGAATGGGTTGCT |
