# Supplementary material for: Retinoic Acid Induces Embryonic Stem Cell Differentiation by Altering Both Encoding RNA and microRNA Expression
Source: PLoS One. 2015 Jul 10;10(7):e0132566. doi: 10.1371/journal.pone.0132566 (PMC4498831; doi:10.1371/journal.pone.0132566)
Supplement: S8 Table — Fold change values were provided in comparison with J1 mESCs treated by DMSO. A p value was calculated by three individual samples. (DOC) [file pone.0132566.s009.doc]

**Table S8 HDACs expression level in J1 mESCs during RA treatment for 24h.**

Fold change values were provided in comparison with J1 mESCs treated by DMSO. P-value was calculated by three individuals samples.

| Gene Symbol Name | P-value | Fold Change |
| --- | --- | --- |
| Hdac2 | 0.001112 | 0.443861044 |
| Hdac11 | 0.598165 | 0.953787645 |
| Hdac4 | 0.02546 | 0.590897582 |
| Hdac8 | 0.000971 | 0.407217086 |
| Hdac10 | 0.300306 | 0.894795558 |
| Hdac9 | 0.000313 | 1.872878097 |
| Hdac5 | 0.003225 | 1.288422714 |
| Hdac3 | 0.144619 | 1.252674858 |
| Hdac6 | 0.005445 | 0.805367969 |
| Hdac7 | 1.37E-05 | 4.518042995 |
| Hdac1 | 0.150327 | 0.876666485 |
